# Tick-borne encephalitis virus subtypes emerged through rapid vector switches rather than gradual evolution

**DOI:** 10.1002/ece3.1301

**Published:** 2014-10-24

**Authors:** Sergey Y Kovalev, Tatyana A Mukhacheva

**Affiliations:** Laboratory of Molecular Genetics, Department of Biology, Ural Federal UniversityLenin Avenue 51, Yekaterinburg, 620000, Russia

**Keywords:** Clusteron, molecular clock, quantum evolution, tick hybrids, tick-borne encephalitis virus, vector switch

## Abstract

Tick-borne encephalitis is the most important human arthropod-borne virus disease in Europe and Russia, with an annual incidence of about 13 thousand people. Tick-borne encephalitis virus (TBEV) is distributed in the natural foci of forest and taiga zones of Eurasia, from the Pacific to the Atlantic coast. Currently, there are three mutually exclusive hypotheses about the origin and distribution of TBEV subtypes, although they are based on the same assumption of gradual evolution. Recently, we have described the structure of TBEV populations in terms of a clusteron approach, a clusteron being a structural unit of viral population [Kovalev and Mukhacheva (2013) *Infect. Genet. Evol*., 14, 22–28]. This approach allowed us to investigate questions of TBEV evolution in a new way and to propose a hypothesis of quantum evolution due to a vector switch. We also consider a possible mechanism for this switch occurring in interspecific hybrids of ticks. It is necessarily accompanied by a rapid accumulation of mutations in the virus genome, which is contrary to the generally accepted view of gradual evolution in assessing the ages of TBEV populations. The proposed hypothesis could explain and predict not only the formation of new subtypes, but also the emergence of new vector-borne viruses.

## Introduction

Tick-borne encephalitis (TBE) is a natural focal transmissible infection, widespread in Eurasia from Western Europe to northern Japan. TBE is the most important arthropod-borne virus disease in Europe and Russia with an annual incidence of about 13 thousand people (Suss 2011). The causative agent, a tick-borne encephalitis virus (TBEV), belongs to the genus *Flavivirus* of the family *Flaviviridae* and forms a TBE complex, which includes Louping ill virus, Langat virus, Powassan virus, Omsk hemorrhagic fever virus, and Kyasanur Forest disease virus. The epidemiology of TBE is closely related to the ecology and biology of ixodid ticks. TBEV circulation in natural foci requires that ticks act as vector and virus reservoir, with vertebrate hosts serving as the blood source and making possible cofeeding TBEV transmission between ticks (Labuda et al. 1993).

The TBEV genome is a positive single-stranded RNA molecule, approximately 11,000 bases in length, which has a single reading frame encoding a polyprotein (Chambers et al. 1990). The only demonstrated mechanism of genetic variation in TBEV is a mutation process via single nucleotide substitutions. Recombination, although detected computationally (Bertrand et al. 2012; Fajs et al. 2012; Norberg et al. 2013), has not been shown experimentally for tick-borne flaviviruses and is not discussed in this study. The rate of mutations in RNA viruses is high and estimated to be about 10^−2^–10^−5^ nucleotide substitutions per site per year (Holmes 2009).

Phylogenetic analysis revealed three subtypes of TBEV, with 15.2–16.4% and 6.2–6.9% differences on nucleotide and amino acid level, respectively (Kozlova et al. 2013). European subtype (TBEV-Eu) is widely distributed in Europe and the European part of Russia (Ecker et al. 1999; Lundkvist et al. 2001; Haglund et al. 2003) while Far Eastern (TBEV-FE) and Siberian (TBEV-Sib) subtypes are spread from Japan and the Far East of Russia to the Baltic countries (Lundkvist et al. 2001; Mickiene et al. 2001; Jaaskelainen et al. 2006). Each TBEV subtype is characterized by a specific amino acid signature of the E protein that is used for classification purposes (Ecker et al. 1999). Besides three conventional subtypes, 178–79 and “886–84 group” strains are proposed to fourth and fifth TBEV subtypes, respectively (Demina et al. 2010, 2012; Kozlova et al. 2013).

Evolution of TBEV as a key member of the TBE complex viruses is always of great scientific interest. There are three main hypotheses for the origin and spread of TBEV. The first hypothesis, based on the analysis of the E gene sequences, proposed that TBE complex viruses had appeared in the Far East around 2500 years ago and spread from East to West Eurasia in a clinal way (Zanotto et al. 1995). Recently, however, this hypothesis was called into question. In particular, analysis of the E gene sequences allowed one research group to conclude that TBEV had originated from Europe 2400–3200 years ago, and distributed from West to East Eurasia, that is, in the opposite direction (Subbotina and Loktev 2012). Another research group proposed a compromise scenario: analysis of complete genome sequences led the authors to suggest Western Siberia as the center of TBEV origin 1800–4900 years ago, with bidirectional distribution to West and East Eurasia (Heinze et al. 2012). So, there are several alternative points of view, all based on the same concept of gradual evolution that assumes a chronologically constant (or nearly constant) rate for both short-term and long-term evolutionary changes.

It is well known that TBEV subtypes exhibit a restricted vector range, TBEV-Eu being transmitted by *Ixodes ricinus* whereas TBEV-FE and TBEV-Sib subtypes are both adapted to *Ixodes persulcatus* ticks. Distribution areas of these ticks overlap with the sympatric zone which occupies a significant part of the East European Plain. Given the essential role of the vector in the flavivirus evolution (Alekseev 1993; Gaunt et al. 2001; Votyakov et al. 2002) and the high rate of mutation in the genome of RNA viruses, it seems logical to explain the emergence of new virus subtypes through rapid vector switches (quantum shifts) rather than gradual evolution. The difference between these methodological approaches is as follows: quantum evolution suggests a drastic shift to the new adaptive zone, that is, new vector species, with a rapid change in the genetic and phenotypic characteristics and without intermediate stages (Simpson 1944), while classical gradualism presents the long-term evolution as a linear accumulation of mutations followed by the natural selection.

Recently, a new approach, based on the clusteron as a basic unit of population structure, has been proposed for the study of TBEV populations (Kovalev and Mukhacheva 2013). A clusteron consists of strains with identical amino acid sequences of the E glycoprotein fragment, as a rule phylogeographically close and having a certain type of territorial distribution. It was shown that clusteron composition, size, and age could solve questions regarding the evolution, origin, and distribution of natural TBE foci (Kovalev and Mukhacheva 2014).

In this study, on the basis of the clusteron structure of all three subtypes of TBEV and general assumptions about the evolution of RNA viruses, a hypothesis of quantum evolution of TBEV through vector switches is proposed, as well as possible mechanisms for the emergence of new vector-borne viruses.

## Materials and Methods

The study involved 1104 nucleotide sequences of the E gene fragments of all three TBEV subtypes deposited in GenBank: 693 TBEV-Sib, 365 TBEV-Eu, and 146 TBEV-FE. Among them, 491 sequences were determined by the authors during 10 years of studies on the epidemiology of TBEV in Russia. Information about individual virus strains is available in Table S1.

Phylogenetic analysis was carried out based on the nucleotide sequences of E gene fragment (from 311 to 762 nt without primer annealing sites) and the deduced amino acid sequence (from 104 to 254 aa). Alignment, phylogenetic analysis, and tree construction were performed using Mega v.5.0 (Tamura et al. 2011). The evolutionary distances were estimated by Maximum Likelihood using the 2-parameter model of Kimura (Kimura 1980). The phylogenetic network was constructed using Phylogenetic Network Software v. 4.6.1.0 (http://fluxus-engineering.com), using the Median-joining algorithm (Bandelt et al. 1999).

Strains and isolates were grouped in clusterons, sharing the same amino acid sequence of the E protein fragment and being phylogenetically related, according to the approach proposed earlier (Kovalev and Mukhacheva 2013). The minimum number of strains in a clusteron was three for TBEV-Sib and two for TBEV-FE and TBEV-Eu, as only a small number of sequences of these two subtypes were available. Single strains or groups of two identical strains for TBEV-Sib, and single strains for TBEV-FE and TBEV-Eu were named as unique. The clusteron name consists of two characters, the first is the number of the subtype (1-TBEV-FE, 2-TBEV-Eu, and 3-TBEV-Sib), and the second is a letter attributed to a specific amino acid signature. The designations of strains belonging to one clusteron, but different phylogenetic lineages, were complemented by a superscript (*3A*^*2*^*, 3C*^*2*^*, 3F*^*2*^*, 3L*^*2*^) (Kovalev and Mukhacheva 2013).

Evolutionary ages of clusterons were calculated based on the previously determined rate of nucleotide substitution, 1.56 ± 0.29 × 10^−4^ synonymous substitutions per site per year (Kovalev et al. 2009).

## Results

**Figure 1:**
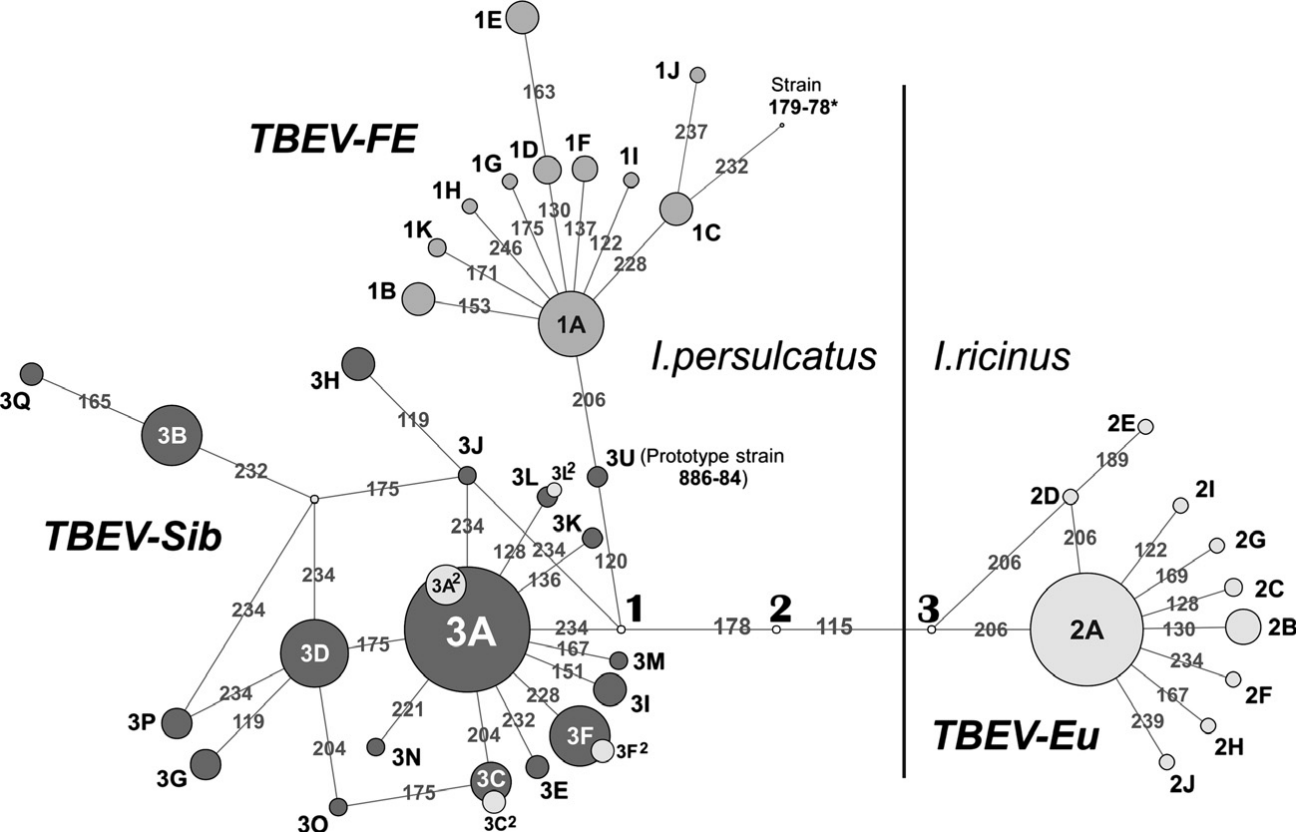
Phylogenetic network of TBEV clusterons constructed on the basis of the sequences of the E protein fragment for all three subtypes. Three domains corresponding to the TBEV subtypes as well as relationships between all clusterons are shown. Clusterons are designated as described in the text and transition points by numbers in bold (see Results). *Sequence of the unique strain 179-78, being proposed as the fourth TBEV subtype, is included in the analysis to show its relationships with clusterons.

**Figure 2:**
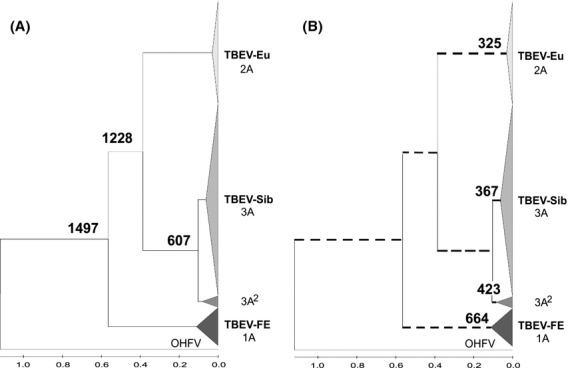
Two approaches to the calculation of the evolutionary age of TBEV. (A) Assuming the hypothesis of gradual evolution (genetic distances are calculated between “clusteron-founders” and putative common ancestor). The age of a branch corresponds to the age of the ancestor. (B) Assuming the hypothesis of quantum evolution resulting in a dramatic change of genetic features and nonlinear accumulation of mutations (genetic distances are calculated within “clusteron-founders”). TBEV subtypes or lineages are likely to emerge not from the putative ancestor but rather directly from the existing viral forms.

The TBEV population, represented by the sequences of 1104 strains of all three subtypes, was subjected to the clusteron approach and visualized in the form of a clusteron structure (Fig. 1). As has been shown for the TBEV-Sib, such a structure could reflect both phenotypic and phylogenetic relationships between clusterons (Kovalev and Mukhacheva 2013). The TBEV clusteron structure, presented as a phylogenetic network, is subdivided into three domains, corresponding to the subtypes, that is, TBEV-FE, TBEV-Eu, and TBEV-Sib (Fig. 1). Each subtype has the same pattern consisting of “clusteron-founder” and its derivatives. “Clusteron-founders” are the greatest in number (*1A, 2A, 3A*) and are encountered by substantially smaller “clusteron-derivatives” of first, second, etc. levels, differing from the “founder” by one, two or more amino acid substitutions (these and other terms used for the further discussion are defined in Table 1, for easier reading). The clusteron structure of TBEV-FE and TBEV-Eu turned out to be relatively simple with only one “clusteron-founder”: *1A* and *2A*, respectively. The structure of the TBEV-Sib is more complex and consists of three subdomains corresponding to three phylogenetic lineages. Indeed, the “clusteron-founder” *3A* forms a subdomain corresponding to the Asian phylogenetic lineage, while clusterons *3A*^*2*^ and *3D* belong to South-Siberian and East European (Baltic) lineages, respectively (Kovalev and Mukhacheva 2013) (Fig. 1, Table 2). The surprising thing is that clusteron structures of Asian and South-Siberian phylogenetic lineages are similar, that is, clusterons *3A* and *3A*^*2*^, *3C* and *3C*^*2*^, *3F* and *3F*^*2*^, *3L* and *3L*^*2*^, despite being phylogenetically different (Fig. 2 for clusterons *3A* and *3A*^*2*^), had the same amino acid signatures (Fig. 1).

**Table 1:** Definition of the terms used in this study

Term	Definition
Vector switch	Change of main arthropod vector by a virus acquiring the ability to circulate in natural populations of the new vector for a long time
Quantum shift	Drastic shift to the new adaptive zone with a rapid change in the genetic and phenotypic characteristics accompanied by the nonlinear accumulation of mutations
Quantum evolution	Step-like pattern of evolution comprising rapid genetic and phenotypic changes followed by long periods when the species evolves very little
Clusteron	A group of TBEV strains with identical amino acid sequences of the E glycoprotein fragment, as a rule phylogeographically close, and having a certain type of territorial distribution
Clusteron structure	Quantitative and qualitative composition of clusterons, visualized as a phylogenetic network
Clusteron-founder	The greatest (in number of strains) clusteron with maximum fitness
Clusteron-derivatives	Clusterons differing from the “clusteron-founder” by one or several amino acid substitutions
Transition point	An amino acid sequence containing deleterious mutations that are normally purged from the virus population

**Table 2:** Evolutionary ages of the major TBEV clusterons

Clusteron (number of strains)*	The number of synonymous substitutions	The maximum genetic distance (nucleotide substitutions)	Evolutionary age (years)
TBEV-FE			
1A (64)	103	47	664 (560–815)
1B (9)	18	18	254 (214–312)
1C (4)	46	38	536 (452–659)
1D (3)	0	0	Recently
1E (5)	1	1	Recently
1F (3)	0	0	Recently
1H (8)	1	1	Recently
TBEV-Sib			
Asian group			
3A (300)	138	26	367 (309–451)
3C (13)	33	21	296 (250–364)
3F (40)	57	21	296 (250–364)
3J (4)	22	21	296 (250–364)
3H (13)	33	18	254 (214–312)
3K (8)	22	16	226 (190–277)
3N (3)	19	17	240 (202–294)
3V (3)	13	12	169 (143–208)
3E (5)	14	14	197 (166–242)
3M (4)	15	14	197 (166–242)
3I (11)	3	3	42 (36–52)
South-Siberian group			
3A^2^ (19)	51	30	423 (357–520)
Eastern-European group			
3D (58)	71	22	310 (262–381)
3G (9)	12	12	169 (143–208)
3P (8)	15	11	155 (131–191)
3B (36)	6	3	42 (36–52)
3Q (5)	3	2	28 (23–34)
3O (4)	1	1	14 (12–17)
Buryat–Mongolian group			
3U (4)	6	6	85 (71–104)
TBEV-Eu			
2A (164)	103	23	325 (273–398)
2B (23)	24	14	197 (166–242)
2C (7)	17	14	197 (166–242)
2F (9)	15	13	184 (155–225)

* Clusterons fewer in number than three strains are not shown.

Spatiotemporal analysis of the TBEV clusteron structure allowed us to make a number of observations. Firstly, the age hierarchy among clusterons within a subtype was observed, that is, “clusteron-founders” are always older than their derivative clusterons (Table 2).

Secondly, there is an age hierarchy among the “clusteron-founders” themselves and their age changes as *1A* > (*3A*^*2*^*>3A>3D*) > *2A*, corresponding to the rule “the more to the west, the younger the clusteron is”. The age of the oldest “clusteron-founder” *1A* TBEV-FE is over 650 years, the youngest, *2A* TBEV-Eu, about 300 years, and the ages of the TBEV-Sib *3A*^*2*^*, 3A, 3D* are of intermediate values, that is, 423, 367, and 310 years, respectively (Table 2).

Thirdly, it was shown that the “clusteron-founders” of TBEV subtypes differ in several amino acid substitutions. Thus, the clusteron *1A* TBEV-FE could be linked in the phylogenetic network with *3A* (*3A*^*2*^*)* TBEV-Sib via the clusteron *3U* and *transition point 1*. Similarly, clusterons *1A* and *3A* (*3A*^*2*^*)* are linked with the clusteron 2A TBEV-Eu via five and four transitions, respectively, by means of *transition points 1, 2,* and *3*. Except for the clusteron *3U*, represented by strains of Buryat–Mongolian phylogenetic lineage (prototype strain 884-84) (Table S1), no strain with an amino acid sequence matching putative *transition points* was found (Fig. 1).

Evolutionary ages were calculated for each clusteron individually, considering them as emerging one from another, without common ancestors (see Discussion). For comparison, ages of the same clusterons were estimated based on the conventional method, assuming a common ancestral node for any two clusterons and the constant rate of mutations. The difference of the two approaches to the calculation is given in Fig. 2 and discussed below.

## Discussion

Gradual evolution as a basic concept for the evolution of tick-borne flaviviruses has resulted in several contradictory evolutionary scenarios. It would be more appropriate to consider alternative concepts of speciation. In the present paper, the clusteron approach was used to hypothesize the emergence of new viruses by means of quantum shifts through vector switches.

### The structure and age of the viral population

The structure of TBEV consists of a “clusteron-founder”, the greatest set of strains with the maximum fitness, derivative clusterons, characterized by a lower fitness, and unique isolates (Fig. 1). As the derivative clusterons are always younger compared to the “clusteron-founder” (Table 2), they were not used to calculate evolutionary ages. This is also true for the unique strains generated as a spectrum of mutants by a viral RNA-dependent RNA polymerase. On these grounds, it can be assumed that the real age of a TBEV subtype corresponds to the age of its “clusteron-founder”.

Considering the age hierarchy of clusterons (*1A* > (*3A*^*2*^*; 3A*) > *2A*) and following the rule “the more to the west, the younger the clusteron is”, it can be concluded that the age of TBEV corresponds to the age of the oldest “clusteron-founder”, that is, *1A* TBEV-FE, being about 660 years (Table 2). This is confirmed by the results of Japanese scientists who have shown TBEV-FE to have been introduced to Japan only during the last 260–430 years (Hayasaka et al. 1999; Suzuki 2007). Apparently, TBEV-FE in Japan has been transmitted by migratory birds between Russia and Japan at least three times during several hundred years (Suzuki 2007). It is unlikely that TBEV-FE, although having emerged on the mainland thousands of years ago, could spread to the nearby Japanese Islands only in the last few centuries.

The emergence of primary foci of TBEV in the Far East confirms the previously proposed hypothesis about the clinal distribution of TBEV from East to West Eurasia (Zanotto et al. 1995). However, the question about age estimation differences, that is, 660 versus 2500 years, necessarily arises.

### The origins of TBEV-Eu and TBEV-Sib

**Figure 3:**
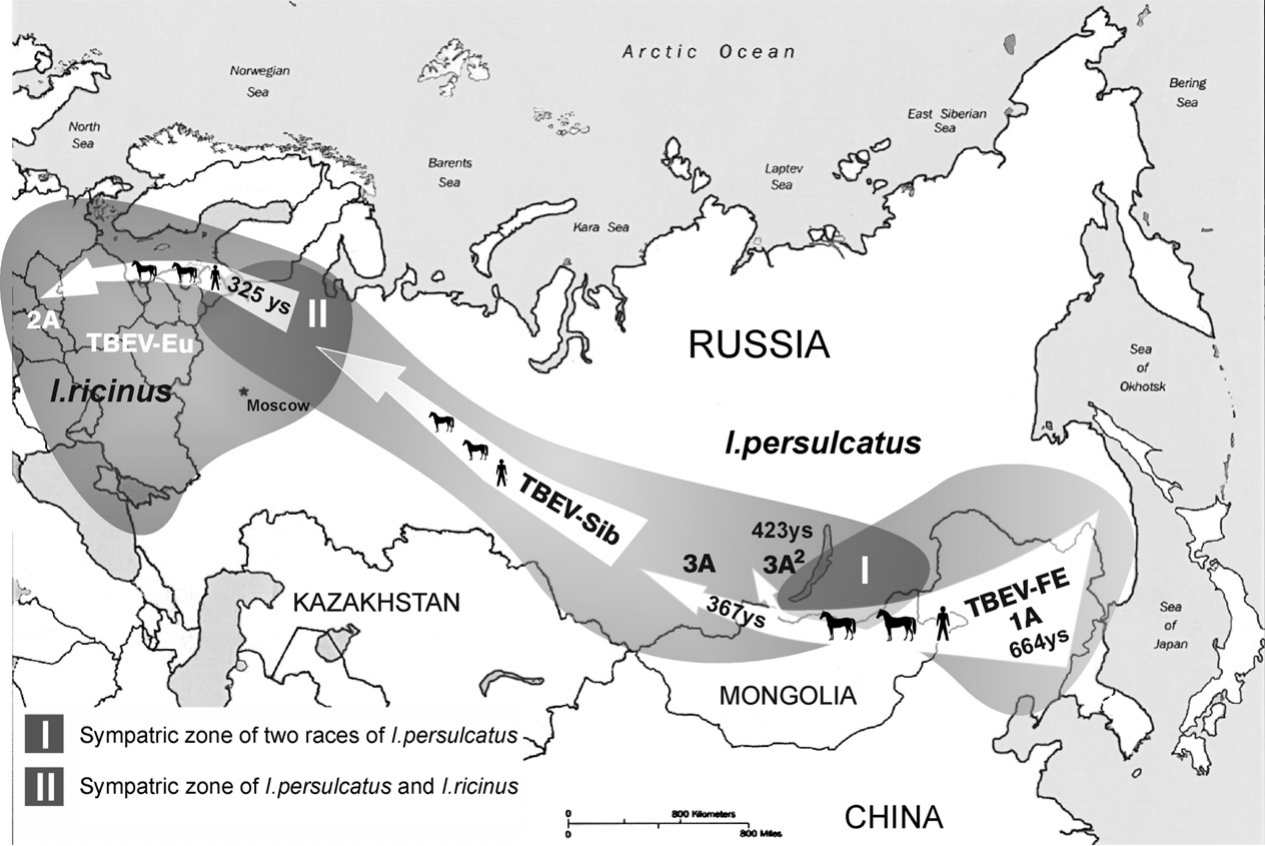
A plausible scenario for TBEV evolution and its spread over Eurasia. The proposed route of the virus distribution, associated with anthropogenic factors, the ages of subtypes, and sympatric zones are shown.

The age of TBEV-Eu, about 300 years (Table 2), is almost identical to the time of appearance of TBEV-Sib strains in northwest Russia as a result of colonization of Western Siberia by Europeans in the early XVII century (Fig. 3) (Kovalev et al. 2009). It is important that the northwestern part of Russia is a sympatric zone where the distribution areas of two ticks, *I. persulcatus* and *I. ricinus,* overlap (Fig. 3, zone II). Therefore, considering the great genetic distance (15.2% on nucleotide level) between TBEV-Sib and TBEV-Eu, we could hypothesize that TBEV-Eu must have emerged from TBEV-Sib very quickly by means of genetic shift as a result of adaptation to a new arthropod vector, *I. ricinus*. This assumption corresponds to the theory of quantum evolution, according to which speciation occurs explosively in a short period of time (Simpson 1944).

One of the most important questions in TBEV evolution is the origin of TBEV-Sib as the most widely distributed subtype. According to the clusteron structure and the rule “the more to the west, the younger the clusteron is”, it can be assumed that TBEV-Sib originated from TBEV-FE by means of a vector switch as well (Fig. 1). At first glance, as the vector of TBEV-Sib and TBEV-FE is the same tick, *I. persulcatus*, it is difficult to justify the emergence of TBEV-Sib in the same way as TBEV-Eu. However, significant geographic variation in morphometric parameters was shown between *I. persulcatus* collected in Karelia, Altai, Sayan, Tien Shan Mountains, and the Far East (Filippova 1985). Such variability suggests an intraspecific structure of *I. persulcatus*, consisting of at least two subpopulations or races – western (area from Europe to Baikal Lake) and eastern (Far East). Their sympatric zone is presumably located in Buryatia, Northern Mongolia, the Trans-Baikal Territory and the Irkutsk region (Fig. 3, zone I). So, TBEV-Sib might emerge from the TBEV-FE as the result of a quantum shift from the eastern race of *I. persulcatus* to the western one.

The complex clusteron structure of TBEV-Sib (Fig. 1) allowed us to assume that there were, in fact, several quantum shifts resulting in the formation of different phylogenetic lineages. Indeed, this fact could explain the formation of South-Siberian and Asian lineages, which are genetically different without being geographically isolated and share the same phenotype (their “clusteron-founders” *3A*^*2*^ and *3A* are identical in amino acid level). The first quantum shift was estimated to take place about 420 years ago and lead to the formation of the South-Siberian phylogenetic lineage (clusteron *3A*^*2*^), and the second, about 370 years ago, resulted in the formation of the Asian lineage (clusteron *3A*) (Figs. 1, 2B, Table 2). The same environmental conditions, that is, the same tick species and even tick race, probably constrained the genetic variation of the E gene and imposed a certain phenotype on two lineages of independent origin.

Based on the above assumptions, the evolution of TBEV, which lasted no more than 700 years, can be presented as a process of the successive emergence of subtypes as a result of quantum evolution (Figs. 2, 3). The driving force for the spread of the virus through Eurasia was apparently the human factor (Kovalev et al. 2009). Thus, the strains of the older subtype TBEV-FE came to the Trans-Baikal region from the primary foci of the Far East via trade routes between Manchuria and the nations inhabiting Western Siberia and the Trans-Baikal region in the Middle Ages (Chi 1932; Franke and Twitchett 1994). These strains, after the switch to a new race of the vector *I. persulcatus*, gave rise to a new Siberian subtype about 420 years ago. Further spread of the virus through the Urals, northwest Russia, and the Baltic countries was associated with the colonization of Siberia by Europeans in the XVII century (Kovalev et al. 2009). The contact of the Siberian subtype with a new tick species *I. ricinus* within the sympatric zone triggered the second step of the quantum evolution of TBEV, resulting in the emergence of the European subtype (about 300 years ago) (Fig. 3). Once emerged, TBEV-Eu spread rapidly through the range of *I. ricinus*, helped by the high population density and well-developed network of roads.

### The mechanism of quantum evolution

As we have shown, a quantum shift may be caused by an adaptation to a new vector species. However, the reproduction of the virus in a nonspecific vector was shown to be inefficient in laboratory conditions (Růžek et al. 2008). Moreover, many field observations provided evidence that TBEV-Sib could be found in *I. ricinus* as well as TBEV-Eu in *I. persulcatus* and other ixodid ticks (Gritsun et al. 2003; Kim et al. 2009; Jaaskelainen et al. 2011)*,* but it did not lead to the emergence of new virus variants.

In this case, the most plausible evolutionary scenario is based on the phenomenon of the formation of hybrids between closely related species of *Ixodes* ticks. Previously, it was shown that the European tick *I. ricinus* and the Asian tick *I. persulcatus* could form first generation hybrids in laboratory conditions. They were completely sterile when crossed with each other and with parent species. Reproductive isolation is apparently due to genetic incompatibility, as no morphological barriers to cross-species mating have been identified (Balashov et al. 1998). It is logical to assume that the hybrids are formed with a certain frequency in nature. For example, hybrids between two tick species, *Dermacentor andersoni* and *D. variabilis*, were detected in sympatric populations from northwestern North America (Araya-Anchetta et al. 2013). The formation of new TBEV subtypes could be facilitated in tick hybrids because of the probable simultaneous presence of two allelic variants of the virus-specific receptor in their outer cell membranes.

It can be assumed that such an adaptation could occur gradually through the selection of adapted viral variants among a wide range of defective (mutant) viruses generated due to the lack of proofreading activity of RNA-dependent RNA polymerase. In our opinion, it is unlikely because of the properties of the secondary and tertiary structure of the flaviviral genomic RNA, which is sensitive to mutations. It means that even single nucleotide substitutions can destabilize viral RNA as a whole and affect the formation of viral particles. This phenomenon has been shown for certain sequence motifs whose mutations regulated virus growth kinetics (Mandl et al. 1998; Tuplin et al. 2011). Analysis of the full-length coding sequences of TBEV has revealed that nucleotide substitutions are not random, and mutations in one region may depend on distantly located ones (Tjulko and Yakimenko 2012). These correlations constrain genome change and allow either single substitutions or drastic change involving the entire RNA molecule at once. Moreover, in normal conditions, there are deleterious mutations that are purged from the virus population and prevented from being fixed. We have called them *transition points* (Fig. 1). They could be potentially beneficial while adapting to a new vector. The number of *transition points* depends on the evolutionary distance of vectors. In the case of TBEV-FE/TBEV-Sib transition, there is only one point, as vectors are races of the same tick species. TBEV-Sib/TBEV-Eu transition is characterized by three points because of the *I. persulcatus*/*I. ricinus* interspecific barrier. *Transition points* prevent gradual accumulation of mutations and are an essential condition of quantum evolution.

**Figure 4:**
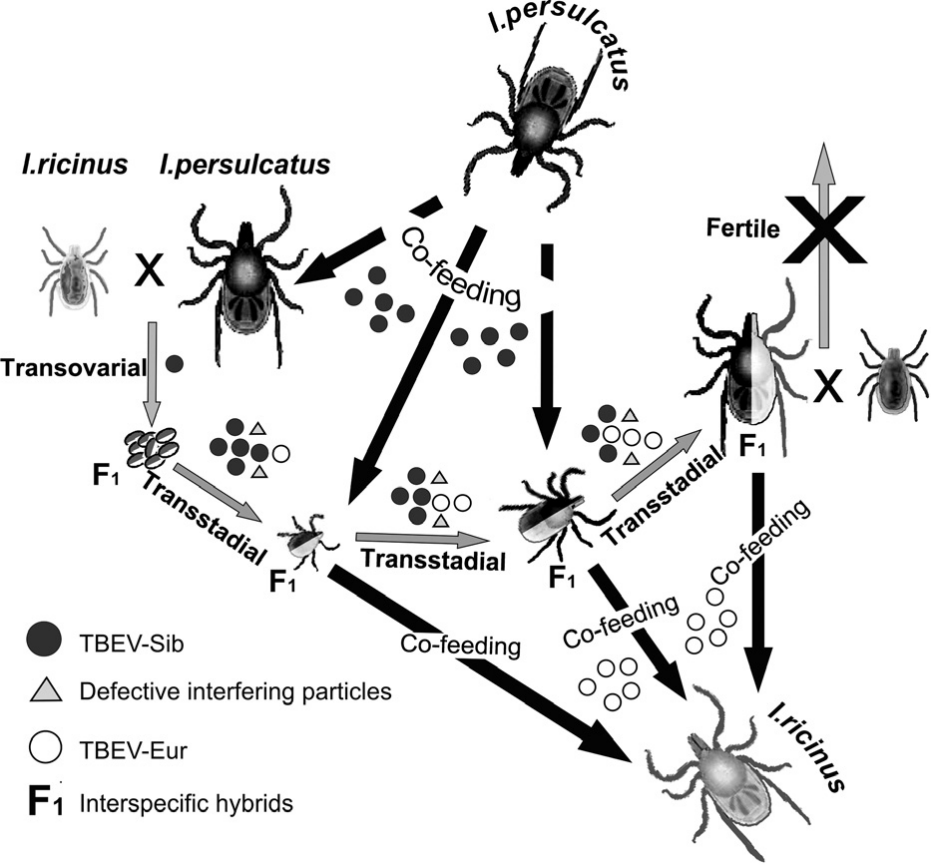
A hypothesized mechanism for the emergence of a new TBEV subtype (TBEV-Eu) in terms of quantum evolution, based on the formation of tick hybrids in the sympatric zone of *Ixodes persulcatus* and *Ixodes ricinus* in northwestern Russia.

Mutations in E gene, which emerge as a result of adaptation to a new vector, could destabilize spatial organization of the genomic RNA. Although such viral particles are usually defective and unable to self-replicate, they can be maintained in a virus population due to the phenomenon of complementation (Moreno et al. 1997; Aaskov et al. 2006). Complementation, facilitated in hybrid cells, allows the virus to overcome transition points and to adapt to an alternative receptor. Thus, one receptor variant, originating from *I. persulcatus*, is able to effectively bind viral E protein of TBEV-Sib and allows virus to enter the cell, while another one, originating from *I. ricinus*, does not have this ability and complicates penetration of the virus. A pool of new viruses, capable of self-replication, accumulates as a result of selection. Subsequently, such viruses, in the case of transmission to *I. ricinus* ticks by means of cofeeding, can begin efficiently circulating in the population of a new vector (Fig. 4).

Emergence of TBEV-Sib from TBEV-FE could be explained as well by means of interracial hybrids of *I. persulcatus*. They should be fertile and therefore occur in the sympatric zone with high frequency. In this case, some transitional forms of TBEV circulating for a long time in *I. persulcatus* interracial hybrids should be found. Actually, such an intermediate virus could be clusteron *3U* (Fig. 1), as the strains of this clusteron (prototype strain 886-84) are found only in the Trans-Baikal region. Moreover, the unique strain 178–79, found in the same area, is also likely to be a transition form. Genetic features of these strains are specific enough to allow some researchers to propose them as fifth and fourth TBEV subtypes, respectively (Demina et al. 2010, 2012; Kozlova et al. 2013) (Table S1). Proceeding from the above, it could be hypothesized that new variants of TBEV have to be found in this area.

Different genetic features of geographically separated *I. persulcatus* populations could explain the reason for the disappearance of the TBEV-FE strains isolated in the Urals and the European part of the former USSR although originating from the Far East (Kovalev et al. 2010). On one hand, TBEV-FE strains could not circulate for a long time in the western race of *I. persulcatus*. On the other hand, ticks of the eastern race of *I. persulcatus,* being unintentionally introduced in small numbers with game animals, could not maintain populations of interracial hybrids and give the chance for new virus variants to emerge.

### Estimation of evolutionary ages

Generally, the evolution of TBEV can be represented alternatively as a gradual evolution or a succession of quantum shifts with periods of gradual evolution. The difference of the two approaches to calculation of evolutionary ages is given in Fig. 2. The first one, based on the hypothesis of gradual evolution, results in a conventional phylogenetic tree with branches whose length corresponds to the genetic distance between existent virus variants and their common ancestor (Fig. 2A). In this case, the age of TBEV is about 1500 years which, in our opinion, tends to be an overestimate. The formal age calculation does not always work well, completely ignoring fundamental biological assumptions, peculiarities of the evolutionary process, co-evolution of viruses, and their vectors and hosts, etc. It can be applied to a gradual stage with a constant rate of mutations. However, adaptation to a new vector results in unpredictable evolutionary changes. For this stage, applying the conventional methods based on constant mutation rate is inappropriate, even if using sophisticated computational approaches and software such as the Bayesian method (Drummond and Rambaut 2007), which is very popular nowadays. The second approach, assuming quantum shifts and periods of gradual evolution, is hard to represent as a tree because the length of branches is only relative due to nonlinear accumulation of mutations (Fig. 2B). The age could be estimated for every group of strains, or their sequences, assuming that their ancestor belongs to the same clade as their descendants. In this case, the age of TBEV is estimated as not exceeding 700 years and returns us to the scenario described above. Such an approach could remove some contradictions indicated in the literature. For example, it was shown that even if the *I. persulcatus –* and *I. ricinus*-borne TBEV strains had evolved independently for about 3000 years, rapid radiation of TBEV-Eu occurred only 300 years ago (Uzcategui et al. 2012). In our opinion, it could be easily explained if TBEV-Eu not only underwent rapid radiation approximately 300 years ago, but also emerged in this time period.

This, the key point of the proposed hypothesis is the idea that new viruses, or subtypes, emerge in a short period of time as a result of vector switches associated with a high acceleration of molecular clock (quantum shift). This hypothesis could be experimentally verified. Indeed, if it is true, it would be very possible to detect the emergence of new viruses, or transitional forms, in interspecific hybrids of ticks or in their mixed cell cultures.

The hypothesis seems to be universal and could explain and predict the formation not only of new subtypes, but also new species of vector-borne viruses, which certainly would be of great practical importance. Understanding the mechanisms of evolutionary processes in natural foci will help to efficiently monitor and control tick-borne encephalitis and other vector-borne viral infections.

## Data Accessibility

Sequence data obtained by the authors have been deposited in GenBank (accessions numbers GU339055, GU444122-GU444286, HM008973-HM008985, JX315719-JX316000, and KC886324-KC886352). Supplementary Table S1 also contains GenBank accession numbers and locality information for all sequences included in the analysis.

## Supporting Information

**Table S1.** Clusterons and unique TBEV strains used in the present study.
