# Supplementary material for: Tick-borne encephalitis virus subtypes emerged through rapid vector switches rather than gradual evolution
Source: Ecol Evol. 2014 Oct 24;4(22):4307–16. doi: 10.1002/ece3.1301 (PMC4267869; doi:10.1002/ece3.1301)
Supplement: Supplementary file 1 — Table S1. Clusterons and unique TBEV strains used in the present study. [file ece30004-4307-SD1.doc]

**Table S1. Clusterons and unique TBEV strains used in the present study**.

| **Strain** | **GenBank**  **Acc. No.** | **Clusteron** | **Year of isolation** | **Source of isolate** | **Geographical origin** | **References** |
| --- | --- | --- | --- | --- | --- | --- |
| Dalnegorsk | FJ402886 | 1A | 1973 | Human brain | Dalnegorsk, Primorsky krai, Russia | Belikov et al. (2008) |
| DXAL-16 | EU089978 | 1A | ? | mouse brain | North-eastern China | Chen et al. (2007) |
| DXAL-18 | EU089979 | 1A | ? | mouse brain | North-eastern China | Chen et al. (2007) |
| Glubinnoe/2004 | DQ862460 | 1A | 2004 | Human brain | Primorsky krai, Russia | Ternovoi et al. (2006) |
| Kavalerovo | FJ402885 | 1A | 1985 | Human brain | Kavalerovo, Primorsky krai, Russia | Belikov et al. (2008) |
| KH98-10 | AB022297 | 1A | 1998 | I. persulcatus | Khabarovsk, Far-Eastern Russia | Hayasaka et al. (1999) |
| KH98-2 | AB022295 | 1A | 1998 | I. persulcatus | Khabarovsk, Far-Eastern Russia | Hayasaka et al. (1999) |
| KH99-m9 | AB049346 | 1A | 1999 | I. persulcatus | Khabarovsk, Far-Eastern Russia | Hayasaka et al. (2001) |
| Kik629/97 | AB237187 | 1A | 1997 | C. rufocanus | Hokkaido, Japan | Hayasaka et al. (1999) |
| Kiparis-94 | JQ825146 | 1A | 1994 | Human blood | Primorsky krai, Russia | Belikov et al. (2012) |
| Kita987/99 | AB237192 | 1A | 1999 | C. rufocanus | Hokkaido, Japan | Yoshizumi et al. (2005) |
| Miz416/97 | AB237184 | 1A | 1997 | C. rufocanus | Hokkaido, Japan | Yoshizumi et al. (2005) |
| Miz660/97 | AB237188 | 1A | 1997 | C. rufocanus | Hokkaido, Japan | Yoshizumi et al. (2005) |
| Oh698/97 | AB237190 | 1A | 1997 | C. rufocanus | Hokkaido, Japan | Yoshizumi et al. (2005) |
| Oh701/97 | AB237191 | 1A | 1997 | C. rufocanus | Hokkaido, Japan | Yoshizumi et al. (2005) |
| O-I-1 | AB022292 | 1A | 1996 | I. ovatus | Japan | Hayasaka et al. (1999) |
| Oshima 08-As | AB753012 | 1A | 2008 | Apodemus speciosus | Japan:Hokkaido, Hokuto | Yoshii et al. (2012) |
| Oshima 3-6 | AB022291 | 1A | ? | Dog blood | Hokkaido, Japan | Hayasaka et al. (1999) |
| Oshima 5-10 | AB001026 | 1A | 1993 | Cerebrospinal fluid | Hokkaido, Japan | Takashimaet al. (1997) |
| Oshima 5-10 | AB062063 | 1A | 1995 | Dog blood | Oshima, Japan | Hayasaka et al. (1999) |
| Oshima C-1 | AB022294 | 1A | 1995 | C. rufocanus | Oshima, Japan | Hayasaka et al. (1999) |
| P-202 | DQ667009 | 1A | 1997 | Human blood | Primorsky krai, Russia | Leonova et al. (2007) |
| P-69 | DQ667007 | 1A | 2000 | Human blood | Primorsky krai, Russia | Leonova et al. (2007) |
| P-73 | DQ667008 | 1A | 1973 | Human brain | Primorsky krai, Russia | Leonova et al. (2007) |
| Primorye-18 | GQ228395 | 1A | 1997 | Human blood | Primorsky krai, Russia | Belikov et al. (2009) |
| Primorye-183 | JQ825153 | 1A | 1991 | Human blood | Primorsky krai, Russia | Belikov et al. (2012) |
| Primorye-202 | JQ825157 | 1A | 1997 | Human blood | Primorsky krai, Russia | Belikov et al. (2012) |
| Primorye-208 | JQ825158 | 1A | 1991 | Human blood | Primorsky krai, Russia | Belikov et al. (2012) |
| Primorye-212 | EU816450 | 1A | 1991 | Human blood | Primorsky krai, Russia | Belikov et al. (2008) |
| Primorye-2239 | HM859895 | 1A | 1985 | ? | Primorsky krai,Russia | Chausov et al. (2010) |
| Primorye-253 | EU816451 | 1A | 1991 | Human blood | Primorsky krai, Russia | Belikov et al. (2008) |
| Primorye-320 | JQ825160 | 1A | 1999 | Human blood | Primorsky krai, Russia | Belikov et al. (2012) |
| Primorye-332 | AY169390 | 1A | 1991 | Human blood | Primorsky krai, Russia | Kulakova et al. (2002) |
| Primorye-437 | JQ825162 | 1A | 1999 | Human blood | Russia: Russian Far East | Belikov et al. (2012) |
| Primorye-69 | EU816453 | 1A | 2000 | Human blood | Primorsky krai, Russia | Belikov et al. (2008) |
| Primorye-739 | JQ825156 | 1A | 1992 | Human blood | Primorsky krai, Russia | Belikov et al. (2012) |
| Primorye-82 | JQ825148 | 1A | 1982 | Human blood | Primorsky krai, Russia | Belikov et al. (2012) |
| Primorye-86 | EU816455 | 1A | 1986 | Human brain | Primorsky krai, Russia | Belikov et al. (2008) |
| Primorye-87 | JQ825149 | 1A | 1987 | Human brain | Primorsky krai, Russia | Belikov et al. (2012) |
| Primorye-89 | FJ906622 | 1A | 1987 | Human brain | Primorsky krai, Russia | Belikov et al. (2009) |
| Primorye-895 | JQ825145 | 1A | 2000 | Human blood | Primorsky krai, Russia | Belikov et al. (2012) |
| Primorye-91 | JQ825150 | 1A | 1991 | Human brain | Primorsky krai, Russia | Belikov et al. (2012) |
| Primorye-92 | HQ201303 | 1A | 1992 | brain of patient | Primorsky krai, Russia | Belikov et al. (2010) |
| Shkotovo-94 | JQ825147 | 1A | 1994 | Human blood | Primorsky krai, Russia | Belikov et al. (2012) |
| SofjinKGG | GU121963 | 1A | 1937 | Human brain | Primorsky krai, Russia | Kozlovskaya et al. (2009) |
| Spassk-72 | JQ825151 | 1A | 1972 | Human brain | Primorsky krai, Russia | Belikov et al. (2012) |
| Svetlogorie | GU121642 | 1A | 2008 | Human brain | Svetlogorie, Primorsky krai, Russia | Belikov et al. (2010) |
| T-blood | AF091019 | 1A | 1939 | Human blood | Perm, Russia | Ecker et al. (1999) |
| Ural-Antipov | FJ214115 | 1A | 1942 | Human blood | Ural region, Russia | Karan et al. (2008) |
| Ural-Belyaeva | FJ214117 | 1A | 1943 | Human blood | Ural region, Russia | Karan et al. (2008) |
| Ural-Ivanova | FJ214116 | 1A | 1942 | Human blood | Ural region, Russia | Karan et al. (2008) |
| Ural-Nina | FJ214119 | 1A | 1943 | blood of patient | Ural region, Russia | Karan et al. (2008) |
| Ural-Ponomarev | FJ214118 | 1A | 1942 | Human blood | Ural region, Russia | Karan et al. (2008) |
| Ural-Troynik | FJ214112 | 1A | 1941 | blood of patient | Ural region, Russia | Karan et al. (2008) |
| Ural-Yulya | FJ214113 | 1A | 1943 | liquor of patient | Ural region, Russia | Karan et al. (2008) |
| VL99-m11 | AB049345 | 1A | 1999 | I. persulcatus | Vladivostok, Russia | Hayasaka et al. (2001) |
| Volkhov-Khromov | FJ214114 | 1A | 1943 | blood of patient | Volkhov, northwestern region, Russia | Karan et al. (2008) |
| Xinjiang-01 | JX534167 | 1A | 2012 | I.persulcatus | China, Xinjiang, Northewestern China | Liu et al. (2012) |
| Zabaikalye 30-00 | KC422667 | 1A | 2000 | Human brain | Zabaikalye, Russia | Adelshin et al. (2012) |
| Zabaikalye 50-03 | KC422666 | 1A | 2003 | Tamias sibiricus | Zabaikalye, Russia | Adelshin et al. (2012) |
| Zabaikalye 64-00 | KC422664 | 1A | 2000 | Microtus maximowiczii ungurensis | Zabaikalye, Russia | Adelshin et al. (2012) |
| Zabaikalye 67-99 | KC422665 | 1A | 1999 | D. silvarum | Zabaikalye, Russia | Adelshin et al. (2012) |
| Zabaikalye-1-98 | JX968560 | 1A | 1998 | Human blood | Zabaikalye, Russia | Adelshin et al. (2012) |
| Zabaikalye-1-98 | JX968560 | 1A | 1998 | Homo sapiens | Russia, Zabaikalye | Adelshin et al. (2012) |
| 80k | GU121965 | 1B | 1970 | Human blood | Sverdlovsk region, Urals, Russia | Kozlovskaya et al. (2009) |
| Ekb4072-1966 | HM008979 | 1B | 1966 | Human blood | Russia, Sverdlovsk region | Kovalev et al. (2010) |
| Ekb4072-1966 | HM008979 | 1B | 1966 | Human blood | Sverdlovsk region, Russia | Kovalev et al. (2010) |
| Irkutsk-48-06 | FJ214157 | 1B | 2006 | Microtus oeconomus | Irkutsk, East Siberia, Russia | Karan et al. (2008) |
| Kemerovo-Phateev | FJ214132 | 1B | 1954 | blood of patient | Kemerovo, West Siberia, Russia | Karan et al. (2008) |
| Kemerovo-YuB-40-67 | FJ214133 | 1B | 1967 | Human blood | Kemerovo, West Siberia, Russia | Karan et al. (2008) |
| Khabarovsk-Obor-4 | FJ214111 | 1B | 1937 | Human brain | Khabarovsk, Far East, Russia | Karan et al. (2008) |
| Novosibirsk-11416 | FJ214158 | 1B | 1991 | ? | Novosibirsk, West Siberia, Russia | Karan et al. (2008) |
| Sofjin-Chumakov | KC806252 | 1B | 1937 | Human brain | Primorsky krai, Russia | Vorovich et al. (2013) |
| Sofjin-HO | AB062064 | 1B | ? |  | Khabarovskiy kray, Far-East, Russia | Goto et al. (2001) |
| SofjinKSY | JF819648 | 1B | 1937 | Human blood | Primorsky krai, Russia | Kovalev et al. (2012) |
| 205 | DQ989336 | 1C | 1973 | I. persulcatus | Primorsky krai, Russia | Safronov et al. (1990) |
| 205 | JX498939 | 1C | 1973 | I. persulcatus | Primorsky krai, Russia | Chausov et al. (2012) |
| 205KGG | GU121964 | 1C | 1973 | I. persulcatus | Khabarovskiy kray, Far-East, Russia | Kozlovskaya et al. (2009) |
| D1283 | AB049347 | 1C | 1998 | Human brain | Khabarovsk, Far-Eastern Russia | Hayasaka et al. (2001) |
| PK-36 | GU121967 | 1C | 1982 | I. persulcatus | Primorskiy kray, Far-East, Russia | Kozlovskaya et al. (2009) |
| Primorye-196 | JQ825155 | 1C | 2000 | Human blood | Russia: Russian Far East | Belikov et al. (2012) |
| Primorye-52 | JQ825154 | 1C | 1999 | Human blood | Primorsky krai, Russia | Belikov et al. (2012) |
| Ekb100-2007 | HM008973 | 1D | 2007 | I.persulcatus | Sverdlovsk region, Russia | Kovalev et al. (2010) |
| Ekb100-2007 | HM008973 | 1D | 2007 | I.persulcatus | Russia, Sverdlovsk region | Kovalev et al. (2010) |
| Ekb101-2007 | HM008974 | 1D | 2007 | I.persulcatus | Sverdlovsk region, Russia | Kovalev et al. (2010) |
| Ekb101-2007 | HM008974 | 1D | 2007 | I.persulcatus | Russia, Sverdlovsk region | Kovalev et al. (2010) |
| N132 | AF091013 | 1D | 1979 | I. persulcatus | Vladivostok, Russia | Ecker et al. (1999) |
| Ekb187-1969 | HM008982 | 1E | 1969 | I.persulcatus | Russia, Sverdlovsk region | Kovalev et al. (2010) |
| Ekb187-1969 | HM008982 | 1E | 1969 | I.persulcatus | Sverdlovsk region, Russia | Kovalev et al. (2010) |
| Ekb4100-1978 | HM008981 | 1E | 1978 | Human blood | Russia, Sverdlovsk region | Kovalev et al. (2010) |
| Ekb4100-1978 | HM008981 | 1E | 1978 | Human blood | Sverdlovsk region, Russia | Kovalev et al. (2010) |
| Ekb5127-1985 | HM008983 | 1E | 1985 | I.persulcatus | Sverdlovsk region, Russia | Kovalev et al. (2010) |
| Ekb5127-1985 | HM008983 | 1E | 1985 | I.persulcatus | Russia, Sverdlovsk region | Kovalev et al. (2010) |
| Ekb5308-1978 | HM008984 | 1E | 1978 | Human blood | Sverdlovsk region, Russia | Kovalev et al. (2010) |
| Ekb5308-1978 | HM008984 | 1E | 1978 | Human blood | Russia, Sverdlovsk region | Kovalev et al. (2010) |
| Ekb54-1968 | HM008985 | 1E | 1968 | Human blood | Russia, Sverdlovsk region | Kovalev et al. (2010) |
| Ekb54-1968 | HM008985 | 1E | 1968 | Human blood | Sverdlovsk region, Russia | Kovalev et al. (2010) |
| Ekb183-1973 | HM008976 | 1F | 1973 | Human blood | Sverdlovsk region, Russia | Kovalev et al. (2010) |
| Ekb183-1973 | HM008976 | 1F | 1973 | Human blood | Russia, Sverdlovsk region | Kovalev et al. (2010) |
| Ekb295-1975 | HM008977 | 1F | 1975 | I.persulcatus | Sverdlovsk region, Russia | Kovalev et al. (2010) |
| Ekb295-1975 | HM008977 | 1F | 1975 | I.persulcatus | Russia, Sverdlovsk region | Kovalev et al. (2010) |
| Ekb73-1978 | HM008978 | 1F | 1978 | I.persulcatus | Russia, Sverdlovsk region | Kovalev et al. (2010) |
| Ekb73-1978 | HM008978 | 1F | 1978 | I.persulcatus | Sverdlovsk region, Russia | Kovalev et al. (2010) |
| Kam586/97 | AB237185 | 1G | 1997 | C. rufocanus | Hokkaido, Japan | Yoshizumi et al. (2005) |
| Kam588/97 | AB237186 | 1G | 1997 | C. rufocanus | Hokkaido, Japan | Yoshizumi et al. (2005) |
| Crimea | AF091008 | 1H | 1987 | I. ricinus | Crimea, Ukraine Крым | Ecker et al. (1999) |
| Primorye-270 | EU816452 | 1H | 1991 | Human blood | Primorsky krai, Russia | Belikov et al. (2008) |
| Primorye-274 | JQ825159 | 1H | 1999 | Human blood | Primorsky krai, Russia | Belikov et al. (2012) |
| Primorye-345 | JQ825161 | 1H | 1999 | Human blood | Primorsky krai, Russia | Belikov et al. (2012) |
| Primorye-75 | JQ825152 | 1H | 1999 | Human blood | Primorsky krai, Russia | Belikov et al. (2012) |
| Primorye-750 | JQ825163 | 1H | 1998 | Human blood | Primorsky krai, Russia | Belikov et al. (2012) |
| Primorye-823 | JQ825164 | 1H | 2000 | Human blood | Primorsky krai, Russia | Belikov et al. (2012) |
| Primorye-828 | JQ825144 | 1H | 1998 | Human blood | Primorsky krai, Russia | Belikov et al. (2012) |
| DXAL5 | AY178833 | 1I | ? | Unknown | North-eastern China | Chen et al. (2002) |
| MDJ-02 | JF316707 | 1I | 2010 | Human blood | China: Mudanjiang | Zhang et al. (2011) |
| Ekaterinburg-Bersenev | FJ214121 | 1J | 1960 | blood of patient | Ekaterinburg, Ural, Russia | Karan et al. (2008) |
| Ekaterinburg-Vinokurov | FJ214120 | 1J | 1959 | blood of patient | Ekaterinburg, Ural, Russia | Karan et al. (2008) |
| Est2546 | DQ393779 | 1K | 1996 | A. agrarius | Estonia | Golovljova et al. (2004) |
| Sofjin | JX498940 | 1K | ? |  |  | Chausov et al. (2012) |
| Sofjin | X07755 | 1K | 1937 | Human brain | Primorsky krai, Russia | Yamshchikov and Pletnev (1988) |
| Sofjin-Ru | JN229223 | 1K | ? | ? | ? | Morozov et al. (2011) |
| 178-79 | EF469661 | 1Unique | 1979 | mouse brain | Irkutsk region, Russia | Karan et al. (1999) |
| DV 936k | GU125722 | 1Unique | 1975 | ticks H. concina | Primorskiy kray, Far-East, Russia | Kozlovskaya et al. (2009) |
| DXAL-12 | EU089977 | 1Unique | ? | mouse brain | North-eastern China | Chen et al. (2007) |
| DXAL-13 | EU089976 | 1Unique | ? | mouse brain | North-eastern China | Chen et al. (2007) |
| DXAL-21 | EU089980 | 1Unique | ? | mouse brain | North-eastern China | Chen et al. (2007) |
| Ekb162-1977 | HM008975 | 1Unique | 1977 | I.persulcatus | Sverdlovsk region, Russia | Kovalev et al. (2010) |
| Ekb162-1977 | HM008975 | 1Unique | 1977 | I.persulcatus | Russia, Sverdlovsk region | Kovalev et al. (2010) |
| Ekb5118-1985 | HM008980 | 1Unique | 1985 | Human blood | Russia, Sverdlovsk region | Kovalev et al. (2010) |
| Ekb5118-1985 | HM008980 | 1Unique | 1985 | Human blood | Sverdlovsk region, Russia | Kovalev et al. (2010) |
| Irkutsk-1861 | JN003205 | 1Unique | 2008 | Homo sapiens | Russia | Kulakova et al. (2011) |
| KH98-5 | AB022296 | 1Unique | 1998 | I. persulcatus | Khabarovsk, Far-Eastern Russia | Hayasaka et al. (1999) |
| MDJ-01 | AY217093 | 1Unique | ? | ? | China | Ma et al. (2003) |
| MDJ-03 | JF316708 | 1Unique | 2010 | Human blood | China: Mudanjiang | Zhang et al. (2011) |
| Oh696/97 | AB237189 | 1Unique | 1997 | C. rufocanus | Hokkaido, Japan | Yoshizumi et al. (2005) |
| Oshima 5-11 | AB022290 | 1Unique | 1995 | Dog blood | Oshima, Japan | Hayasaka et al. (1999) |
| Oshima A-1 | AB022293 | 1Unique | 1995 | A. speciosus | Oshima, Japan | Hayasaka et al. (1999) |
| Primorye-1153 | HQ901366 | 1Unique | 2009 | Human blood | Russia | Chausov et al. (2011) |
| Primorye-501 | HQ901367 | 1Unique | 2010 | Human blood | Russia | Chausov et al. (2011) |
| Primorye-633 | HM859894 | 1Unique | 1978 | ? | Russia | Chausov et al. (2010) |
| Primorye-90 | FJ997899 | 1Unique | 1990 | Human brain | Primorsky krai, Russia | Belikov et al. (2009) |
| Primorye-94 | EU816454 | 1Unique | 1994 | Human blood | Primorsky krai, Russia | Belikov et al. (2008) |
| RK1424 | AF091016 | 1Unique | 1977 | I. persulcatus | Latvia | Ecker et al. (1999) |
| Senzhang | AY174188 | 1Unique | 1953 | Human brain | China | Si et al. (2002) |
| Senzhang | AY182009 | 1Unique | 1953 | Human brain | China | Ma et al. (2002) |
| TBEV-MN-2008 | HM133639 | 1Unique | 2008 | brain tissue | Mongolia | Khasnatinov et al. (2010) |
| 1015 | JF501441 | 2A | 2002 | I. ricinus | Czech Republic: Borovany, South Bohemia | Weidmann et al. (2011) |
| 120 | XXXXXXX* | 2A | 1988 | D.marginatus | Ukraine | Yurchenko et al. (2013) |
| 150 | XXXXXXX* | 2A | 1990 | D.marginatus | Ukraine | Yurchenko et al. (2013) |
| 166 | EF113079 | 2A | 2006 | I. hexagonus | Czech Republic | Ruzek et al. (2006) |
| 200 | JF501437 | 2A | 2000 | I. ricinus | Czech Republic: Borovany, South Bohemia | Weidmann et al. (2011) |
| 235 | EF113081 | 2A | 2006 | Unknown | Czech Republic | Ruzek et al. (2006) |
| 263 | TEU27491 | 2A | 1987 | I. ricinus | Czech Republic | Wallner et al. (1995) |
| 274 | EF113083 | 2A | 2006 | Unknown | Czech Republic | Ruzek et al. (2006) |
| 290 | XXXXXXX* | 2A | 1990 | H.marginatum | Ukraine | Yurchenko et al. (2013) |
| 340 | JF501404 | 2A | 1967 | I. ricinus | Czech Republic: Potepli, Central Bohemia | Weidmann et al. (2011) |
| 39 | JF501403 | 2A | 1966 | I. ricinus | Czech Republic: Potepli, Central Bohemia | Weidmann et al. (2011) |
| 398 | JF501400 | 2A | 1963 | I. ricinus | Czech Republic: Potepli, Central Bohemia | Weidmann et al. (2011) |
| 399 | JF501401 | 2A | 1963 | I. ricinus | Czech Republic: Potepli, Central Bohemia | Weidmann et al. (2011) |
| 40 | JF501402 | 2A | 1966 | I. ricinus | Czech Republic: Potepli, Central Bohemia | Weidmann et al. (2011) |
| 414 | JF501399 | 2A | 1954 | I. ricinus | Czech Republic: Potepli, Central Bohemia | Weidmann et al. (2011) |
| 433 | EF116596 | 2A | 2006 | Unknown | Czech Republic | Ruzek et al. (2006) |
| 465 | JF501405 | 2A | 1967 | squirrel | Czech Republic: Potepli, Central Bohemia | Weidmann et al. (2011) |
| 679 | JF501439 | 2A | 2001 | I. ricinus | Czech Republic: Borovany, South Bohemia | Weidmann et al. (2011) |
| 70 | XXXXXXX* | 2A | 1989 | D.marginatus | Ukraine | Yurchenko et al. (2013) |
| 76 | JF501410 | 2A | 1986 | I. ricinus | Czech Republic: Zdar Kaplice, South Bohemia | Weidmann et al. (2011) |
| 798 | JF501440 | 2A | 2001 | I. ricinus | Czech Republic: Rimov, South Bohemia | Weidmann et al. (2011) |
| 80 | XXXXXXX* | 2A | 1989 | I. ricinus | Ukraine | Yurchenko et al. (2013) |
| 84.2 | HM120875 | 2A | ? | acarid | Russia: Altay | Tikhomirov et al. (2010) |
| 85 | XXXXXXX* | 2A | 1989 | I. ricinus | Ukraine | Yurchenko et al. (2013) |
| 9001 | JF501407 | 2A | 1978 | I. ricinus | Czech Republic: Stechovice, Central Bohemia | Weidmann et al. (2011) |
| 9025 | JF501408 | 2A | 1978 | I. ricinus | Czech Republic: Stechovice, Central Bohemia | Weidmann et al. (2011) |
| A104 | KF151173 | 2A | 1990 | Apodemus flavicollis | Austria | Pfeffer et al. (2013) |
| AB_432_11 | KC154189 | 2A | 2011 | I. ricinus | Germany: South-East Germany, Asbach | Frey et al. (2012) |
| Absettarov | AF091005 | 2A | 1951 | Human blood | St. Petersburg region, Russia | Ecker et al. (1999) |
| AG Brittnau1 | HM468123 | 2A | 2009 | I. ricinus | Switzerland | Gaumann et al. (2010) |
| AG Brittnau3 | HM468125 | 2A | 2009 | I. ricinus | Switzerland | Gaumann et al. (2010) |
| AG Brittnau4 | HM468126 | 2A | 2009 | I. ricinus | Switzerland | Gaumann et al. (2010) |
| AG Brittnau5 | HM468127 | 2A | 2009 | I. ricinus | Switzerland | Gaumann et al. (2010) |
| AG Gipf-Oberfrick | HM468128 | 2A | 2009 | I. ricinus | Switzerland | Gaumann et al. (2010) |
| AG Zofingen1 | HM468129 | 2A | 2009 | I. ricinus | Switzerland | Gaumann et al. (2010) |
| AG Zofingen2 | HM468130 | 2A | 2009 | I. ricinus | Switzerland | Gaumann et al. (2010) |
| Als. I | AF091007 | 2A | 1975 | I. ricinus | Alsace, France | Ecker et al. (1999) |
| BE Belp | HM468131 | 2A | 2009 | I. ricinus | Switzerland | Gaumann et al. (2010) |
| BE Erlenbach i.S.1 | HM468132 | 2A | 2009 | I. ricinus | Switzerland | Gaumann et al. (2010) |
| BE Erlenbach i.S.2 | HM468133 | 2A | 2009 | I. ricinus | Switzerland | Gaumann et al. (2010) |
| BE Erlenbach i.S.3 | HM468134 | 2A | 2009 | I. ricinus | Switzerland | Gaumann et al. (2010) |
| BE Erlenbach i.S.4 | HM468135 | 2A | 2009 | I. ricinus | Switzerland | Gaumann et al. (2010) |
| BE Reichenbach i.K.1 | HM468136 | 2A | 2009 | I. ricinus | Switzerland | Gaumann et al. (2010) |
| BE Reichenbach i.K.2 | HM468137 | 2A | 2009 | I. ricinus | Switzerland | Gaumann et al. (2010) |
| BE Spiez Auwald | HM468138 | 2A | 2009 | I. ricinus | Switzerland | Gaumann et al. (2010) |
| BE Spiez Rustwald1 | HM468139 | 2A | 2009 | I. ricinus | Switzerland | Gaumann et al. (2010) |
| BE Spiez Rustwald2 | HM468140 | 2A | 2009 | I. ricinus | Switzerland | Gaumann et al. (2010) |
| BE Thun | HM468141 | 2A | 2009 | I. ricinus | Switzerland | Gaumann et al. (2010) |
| Bul_175_10 | KC154175 | 2A | 2010 | I. ricinus | Germany: South-East Germany, Burglengenfeld | Frey et al. (2012) |
| Bul_176_10 | KC154176 | 2A | 2010 | I. ricinus | Germany: South-East Germany, Burglengenfeld | Frey et al. (2012) |
| Bul_178_10 | KC154177 | 2A | 2010 | I. ricinus | Germany: South-East Germany, Burglengenfeld | Frey et al. (2012) |
| Bul_179_10 | KC154178 | 2A | 2010 | I. ricinus | Germany: South-East Germany, Burglengenfeld | Frey et al. (2012) |
| Bul_185_10 | KC154180 | 2A | 2010 | I. ricinus | Germany: South-East Germany, Burglengenfeld | Frey et al. (2012) |
| Bul_186_10 | KC154179 | 2A | 2010 | I. ricinus | Germany: South-East Germany, Burglengenfeld | Frey et al. (2012) |
| BUL393 | JF501443 | 2A | 2009 | I. ricinus | Germany: Burglengenfeld, South East Germany | Weidmann et al. (2011) |
| BUL399 | JF501444 | 2A | 2009 | I. ricinus | Germany: Burglengenfeld, South East Germany | Weidmann et al. (2011) |
| CZ-407 | JF501419 | 2A | 1988 | I. ricinus | Czech Republic: Libava, North Moravia | Weidmann et al. (2011) |
| Cz-408 | JF501420 | 2A | 1988 | I. ricinus | Czech Republic: Libava, North Moravia | Weidmann et al. (2011) |
| Est3051 | DQ393775 | 2A | 1996 | I. ricinus | Estonia | Golovljova et al. (2004) |
| Est3053 | DQ393777 | 2A | 1996 | I. ricinus | Estonia | Golovljova et al. (2004) |
| Est3509 | DQ393778 | 2A | 2001 | I. ricinus | Estonia | Golovljova et al. (2004) |
| FS572 | JF501445 | 2A | 2009 | I. ricinus | Germany: Fuerstenstein, South East Germany | Weidmann et al. (2011) |
| H-48 | JQ654655 | 2A | 2002 | Homo sapiens | Slovenia | Durmisi et al. (2012) |
| H-51 | JQ654658 | 2A | 2003 | Homo sapiens | Slovenia | Durmisi et al. (2012) |
| H-52 | JQ654659 | 2A | 2003 | Homo sapiens | Slovenia | Durmisi et al. (2012) |
| H-55 | JQ654662 | 2A | 2007 | Homo sapiens | Slovenia | Durmisi et al. (2012) |
| H-64 | JQ654660 | 2A | 2010 | Homo sapiens | Slovenia | Durmisi et al. (2012) |
| HB_171_11 | KC154185 | 2A | 2011 | I. ricinus | Germany: South-East Germany, Heselbach | Frey et al. (2012) |
| HB_184_11 | KC154195 | 2A | 2011 | I. ricinus | Germany: South-East Germany, Heselbach | Frey et al. (2012) |
| HM_11_11 | KC154182 | 2A | 2011 | I. ricinus | Germany: South-East Germany, Haselmuehl | Frey et al. (2012) |
| HM_148_10 | KC154173 | 2A | 2010 | I. ricinus | Germany: South-East Germany, Haselmuehl | Frey et al. (2012) |
| HM_153_10 | KC154174 | 2A | 2010 | I. ricinus | Germany: South-East Germany, Haselmuehl | Frey et al. (2012) |
| HM_17_11 | KC154183 | 2A | 2011 | I. ricinus | Germany: South-East Germany, Haselmuehl | Frey et al. (2012) |
| HM_219_10 | KC154181 | 2A | 2010 | I. ricinus | Germany: South-East Germany, Haselmuehl | Frey et al. (2012) |
| HM_305_11 | KC154186 | 2A | 2011 | I. ricinus | Germany: South-East Germany, Haselmuehl | Frey et al. (2012) |
| HM_329_11 | KC154187 | 2A | 2011 | I. ricinus | Germany: South-East Germany, Haselmuehl | Frey et al. (2012) |
| HM_377_11 | KC154188 | 2A | 2011 | I. ricinus | Germany: South-East Germany, Haselmuehl | Frey et al. (2012) |
| HM_91_11 | KC154184 | 2A | 2011 | I. ricinus | Germany: South-East Germany, Haselmuehl | Frey et al. (2012) |
| HM467 | JF501446 | 2A | 2009 | I. ricinus | Germany: Haselmuehl, South East Germany | Weidmann et al. (2011) |
| HM474 | JF501447 | 2A | 2009 | I. ricinus | Germany: Haselmuehl, South East Germany | Weidmann et al. (2011) |
| HM475 | JF501448 | 2A | 2009 | I. ricinus | Germany: Haselmuehl, South East Germany | Weidmann et al. (2011) |
| HM483 | JF501449 | 2A | 2009 | I. ricinus | Germany: Haselmuehl, South East Germany | Weidmann et al. (2011) |
| HM498 | JF501450 | 2A | 2009 | I. ricinus | Germany: Haselmuehl, South East Germany | Weidmann et al. (2011) |
| HM554 | JF501451 | 2A | 2009 | I. ricinus | Germany: Haselmuehl, South East Germany | Weidmann et al. (2011) |
| HM666 | JF501452 | 2A | 2009 | I. ricinus | Germany: Haselmuehl, South East Germany | Weidmann et al. (2011) |
| HM685 | JF501453 | 2A | 2009 | I. ricinus | Germany: Haselmuehl, South East Germany | Weidmann et al. (2011) |
| Irkutsk-118-71 | FJ214154 | 2A | 1971 | Citellus undulatus | Irkutsk, East Siberia, Russia | Karan et al. (2008) |
| Irkutsk-134-71 | FJ214155 | 2A | 1971 | Citellus undulatus | Irkutsk, East Siberia, Russia | Karan et al. (2008) |
| Iso 40 | AF091009 | 2A | 1975 | I. ricinus | Schaffhausen, Switzerland | Ecker et al. (1999) |
| Isosaari-5 | HM051169 | 2A | 2005 | Ixodes ricinus | Finland | Jaaskelainen et al. (2010) |
| Kumlinge 25-03 | GU183379 | 2A | 2003 | I. ricinus | Finland | Uzcategui et al. (2012) |
| Kumlinge A 52 | AY268437 | 2A | 1959 | I. ricinus | Kumlinge, Finland | Jaaskelainen et al. (2003) |
| Kumlinge_A52 | GU183380 | 2A | 1959 | I. ricinus | Finland | Uzcategui et al. (2012) |
| Latvia-11686 | AJ319582 | 2A | 2001 | Human blood | Latvia | Lundkvist et al. (2001) |
| Latvia-12718 | AJ319586 | 2A | 2001 | Human blood | Latvia | Lundkvist et al. (2001) |
| Latvia-9793 | AJ319585 | 2A | 2001 | Human blood | Latvia | Lundkvist et al. (2001) |
| LU Dagmarsellen1 | HM468142 | 2A | 2009 | I. ricinus | Switzerland | Gaumann et al. (2010) |
| LU Dagmarsellen2 | HM468143 | 2A | 2009 | I. ricinus | Switzerland | Gaumann et al. (2010) |
| LU Dagmarsellen3 | HM468144 | 2A | 2009 | I. ricinus | Switzerland | Gaumann et al. (2010) |
| ML_M2_A104_1990 | KC154199 | 2A | 1990 | Apodemus flavicollis | Austria: Kleinsemmering | Frey et al. (2012) |
| ML_M3_285_1990 | KC154200 | 2A | 1990 | I. ricinus | Slovakia: Malacky | Frey et al. (2012) |
| ML_M5_CG/223_1990 | KC154201 | 2A | 1990 | Myodes glareolus | Slovakia: Zahorska Ves | Frey et al. (2012) |
| ML_M9_114_1980 | KC154202 | 2A | 1980 | I. ricinus | Slovakia: Plastovce | Frey et al. (2012) |
| N256 | AF091014 | 2A | Unknown | I. ricinus | Minsk, Belarus | Ecker et al. (1999) |
| NW_2A111-120N_11 | KC154193 | 2A | 2011 | I. ricinus | Germany: South-East Germany, Neustadt/Waldnaab | Frey et al. (2012) |
| PA I | FJ360847 | 2A | 2006 | I. ricinus | Germany: Bavaria | Kupca et al. (2008) |
| PA II | FJ360848 | 2A | 2006 | I. ricinus | Germany: Bavaria | Kupca et al. (2008) |
| PA III | FJ360849 | 2A | 2006 | I. ricinus | Germany: Bavaria | Kupca et al. (2008) |
| PA_1R1-4N_11 | KC154191 | 2A | 2011 | I. ricinus | Germany: South-East Germany, Passau | Frey et al. (2012) |
| Pan | AF091015 | 2A | 1957 | Human blood | Moscow, Russia | Ecker et al. (1999) |
| pool 2223 | KC292219 | 2A | 2011 | tick | Germany: Bavaria | Klaus et al. (2012) |
| RO I | FJ360850 | 2A | 2006 | I. ricinus | Germany: Bavaria | Kupca et al. (2008) |
| Salem | EU106868 | 2A | ? | brain tissue Macaca sylvanus | Germany | Hotzel et al. (2007) |
| Salem | FJ572210 | 2A | ? | ? | Germany | Zoeller et al. (2008) |
| Saringe-2009 | KC469073 | 2A | 2009 | I. ricinus | Sweden | Lindblom et al. (2013) |
| Savran_160 | XXXXXXX* | 2A | 1989 | I. ricinus | Ukraine | Yurchenko et al. (2013) |
| Scharl | AF091017 | 2A | 1956 | Human brain | Lower Austria, Austria | Ecker et al. (1999) |
| SH Stein am Rhein | HM468149 | 2A | 2009 | I. ricinus | Switzerland | Gaumann et al. (2010) |
| Simo-2 | HQ228016 | 2A | 2009 | Myodes glareolus | Finland | Jaaskelainen et al. (2010) |
| Simo-38 | HQ228014 | 2A | 2009 | I. persulcatus | Finland | Jaaskelainen et al. (2010) |
| Simo-48 | HQ228015 | 2A | 2009 | I. persulcatus | Finland | Jaaskelainen et al. (2010) |
| Simo-5 | HQ228017 | 2A | 2009 | Myodes glareolus | Finland | Jaaskelainen et al. (2010) |
| Simo-7 | HQ228018 | 2A | 2009 | Myodes glareolus | Finland | Jaaskelainen et al. (2010) |
| Simo-9 | HQ228019 | 2A | 2009 | Myodes glareolus | Finland | Jaaskelainen et al. (2010) |
| SO Oensingen1 | HM468150 | 2A | 2009 | I. ricinus | Switzerland | Gaumann et al. (2010) |
| SO Oensingen2 | HM468151 | 2A | 2009 | I. ricinus | Switzerland | Gaumann et al. (2010) |
| SO Oensingen3 | HM468152 | 2A | 2009 | I. ricinus | Switzerland | Gaumann et al. (2010) |
| SZ Gersau | HM468155 | 2A | 2009 | I. ricinus | Switzerland | Gaumann et al. (2010) |
| T-133 | JF501412 | 2A | 1986 | I. ricinus | Czech Republic: Zdar Kaplice, South Bohemia | Weidmann et al. (2011) |
| T1354 | EU872052 | 2A | ? | ? | Germany | Hoffmann et al. (2008) |
| T1401 | EU872053 | 2A | ? | ? | Germany | Hoffmann et al. (2008) |
| T-155 | JF501413 | 2A | 1986 | I. ricinus | Czech Republic: Zdar Kaplice, South Bohemia | Weidmann et al. (2011) |
| T-177 | JF501411 | 2A | 1986 | I. ricinus | Czech Republic: Zdar Kaplice, South Bohemia | Weidmann et al. (2011) |
| T-239 | JF501417 | 2A | 1987 | I. ricinus | Czech Republic: Zdar Kaplice, South Bohemia | Weidmann et al. (2011) |
| T-321 | JF501418 | 2A | 1987 | I. ricinus | Czech Republic: Zdar Kaplice, South Bohemia | Weidmann et al. (2011) |
| T-717/III | JF501424 | 2A | 1996 | I. ricinus | Czech Republic: Chlum/Decina, North Bohemia | Weidmann et al. (2011) |
| T-718 | JF501425 | 2A | 1996 | I. ricinus | Czech Republic: Chlum/Decina, North Bohemia | Weidmann et al. (2011) |
| T-721 | JF501426 | 2A | 1996 | I. ricinus | Czech Republic: Chlum/Decina, North Bohemia | Weidmann et al. (2011) |
| T-721/III | JF501427 | 2A | 1996 | I. ricinus | Czech Republic: Chlum/Decina, North Bohemia | Weidmann et al. (2011) |
| T-730/I | JF501429 | 2A | 1997 | I. ricinus | Czech Republic: Nova Rice, South Moravia | Weidmann et al. (2011) |
| T-740 | JF501430 | 2A | 1997 | I. ricinus | Czech Republic: Neznabohy, North Bohemia | Weidmann et al. (2011) |
| T-742 | JF501431 | 2A | 1997 | I. ricinus | Czech Republic: Neznabohy, North Bohemia | Weidmann et al. (2011) |
| T-750/II | JF501432 | 2A | 1997 | I. ricinus | Czech Republic: Neznabohy, North Bohemia | Weidmann et al. (2011) |
| T-754/II | JF501433 | 2A | 1997 | I. ricinus | Czech Republic: Neznabohy, North Bohemia | Weidmann et al. (2011) |
| T-828 | JF501434 | 2A | 1999 | I. ricinus | Czech Republic: Usti nad Labem, North Bohemia | Weidmann et al. (2011) |
| T828/II | JF501435 | 2A | 1999 | I. ricinus | Czech Republic: Usti nad Labem, North Bohemia | Weidmann et al. (2011) |
| T-85 | JF501414 | 2A | 1986 | I. ricinus | Czech Republic: Zlata Koruna, South Bohemia | Weidmann et al. (2011) |
| TBE 4387 | X76607 | 2A | 1982 | Bank vole | Southern Slovakia | Labuda et al. (1994) |
| TG Frauenfeld | HM468157 | 2A | 2009 | I. ricinus | Switzerland | Gaumann et al. (2010) |
| TG Lommis1 | HM468158 | 2A | 2009 | I. ricinus | Switzerland | Gaumann et al. (2010) |
| TG Lommis3 | HM468160 | 2A | 2009 | I. ricinus | Switzerland | Gaumann et al. (2010) |
| Toro-2003 | DQ401139 | 2A | 2003 | I. ricinus | Sweden: Toro | Melik et al. (2007) |
| Toro-2003 | DQ401140 | 2A | 2003 | I. ricinus | Sweden: Toro | Johansson et al. (2009) |
| UR Schattdorf1 | HM468168 | 2A | 2009 | I. ricinus | Switzerland | Gaumann et al. (2010) |
| UR Schattdorf2 | HM468169 | 2A | 2009 | I. ricinus | Switzerland | Gaumann et al. (2010) |
| UR Silenen | HM468170 | 2A | 2009 | I. ricinus | Switzerland | Gaumann et al. (2010) |
| UR Sisikon1 | HM468171 | 2A | 2009 | I. ricinus | Switzerland | Gaumann et al. (2010) |
| UR Sisikon2 | HM468172 | 2A | 2009 | I. ricinus | Switzerland | Gaumann et al. (2010) |
| V-361 | JF501422 | 2A | 1989 | Apodemus sylvaticus | Czech Republic: Zdar Kaplice, South Bohemia | Weidmann et al. (2011) |
| V-364 | JF501423 | 2A | 1989 | Myodes glareolus | Czech Republic: Zdar Kaplice, South Bohemia | Weidmann et al. (2011) |
| VD Cudrefin | HM468173 | 2A | 2009 | I. ricinus | Switzerland | Gaumann et al. (2010) |
| VD Rances1 | HM468174 | 2A | 2009 | I. ricinus | Switzerland | Gaumann et al. (2010) |
| VD Rances2 | HM468175 | 2A | 2009 | I. ricinus | Switzerland | Gaumann et al. (2010) |
| VS Raron | HM468176 | 2A | 2009 | I. ricinus | Switzerland | Gaumann et al. (2010) |
| VS Salgesch | HM468177 | 2A | 2009 | I. ricinus | Switzerland | Gaumann et al. (2010) |
| ZG Steinhausen1 | HM468178 | 2A | 2009 | I. ricinus | Switzerland | Gaumann et al. (2010) |
| ZG Steinhausen2 | HM468179 | 2A | 2009 | I. ricinus | Switzerland | Gaumann et al. (2010) |
| ZH Elgg | HM468186 | 2A | 2009 | I. ricinus | Switzerland | Gaumann et al. (2010) |
| ZH Langnau a.A.1 | HM468187 | 2A | 2009 | I. ricinus | Switzerland | Gaumann et al. (2010) |
| ZH Langnau a.A.2 | HM468188 | 2A | 2009 | I. ricinus | Switzerland | Gaumann et al. (2010) |
| ZH Oberstammheim | HM468189 | 2A | 2009 | I. ricinus | Switzerland | Gaumann et al. (2010) |
| ZH Ruemlang | HM468190 | 2A | 2009 | I. ricinus | Switzerland | Gaumann et al. (2010) |
| ZH Unterengstringen | HM468192 | 2A | 2009 | I. ricinus | Switzerland | Gaumann et al. (2010) |
| ZH Zollikon1 | HM468193 | 2A | 2009 | I. ricinus | Switzerland | Gaumann et al. (2010) |
| ZH Zollikon2 | HM468194 | 2A | 2009 | I. ricinus | Switzerland | Gaumann et al. (2010) |
| H-47 | JQ654654 | 2B | 2000 | Homo sapiens | Slovenia | Durmisi et al. (2012) |
| H-49 | JQ654656 | 2B | 2003 | Homo sapiens | Slovenia | Durmisi et al. (2012) |
| H-54 | JQ654661 | 2B | 2006 | Homo sapiens | Slovenia | Durmisi et al. (2012) |
| H-56 | JQ654663 | 2B | 2007 | Homo sapiens | Slovenia | Durmisi et al. (2012) |
| H-57 | JQ654664 | 2B | 2009 | Homo sapiens | Slovenia | Durmisi et al. (2012) |
| H-61 | JQ654668 | 2B | 2009 | Homo sapiens | Slovenia | Durmisi et al. (2012) |
| R-33 | JQ654651 | 2B | 2007 | Apodemus | Slovenia | Durmisi et al. (2012) |
| T-15 | JQ654647 | 2B | 2006 | Ixodes ricinus | Slovenia | Durmisi et al. (2012) |
| T-20 | JQ654648 | 2B | 2006 | Ixodes ricinus | Slovenia | Durmisi et al. (2012) |
| T-23 | JQ654649 | 2B | 2007 | Ixodes ricinus | Slovenia | Durmisi et al. (2012) |
| T-8 | JQ654643 | 2B | 2005 | Ixodes ricinus | Slovenia | Durmisi et al. (2012) |
| TG Aadorf | HM468156 | 2B | 2009 | I. ricinus | Switzerland | Gaumann et al. (2010) |
| TG Thundorf | HM468161 | 2B | 2009 | I. ricinus | Switzerland | Gaumann et al. (2010) |
| TG Waengi1 | HM468162 | 2B | 2009 | I. ricinus | Switzerland | Gaumann et al. (2010) |
| TG Waengi2 | HM468163 | 2B | 2009 | I. ricinus | Switzerland | Gaumann et al. (2010) |
| TG Waengi3 | HM468164 | 2B | 2009 | I. ricinus | Switzerland | Gaumann et al. (2010) |
| TG Waengi4 | HM468165 | 2B | 2009 | I. ricinus | Switzerland | Gaumann et al. (2010) |
| TG Waengi5 | HM468166 | 2B | 2009 | I. ricinus | Switzerland | Gaumann et al. (2010) |
| TG Waengi6 | HM468167 | 2B | 2009 | I. ricinus | Switzerland | Gaumann et al. (2010) |
| ZH Aeugst a.A.1 | HM468182 | 2B | 2009 | I. ricinus | Switzerland | Gaumann et al. (2010) |
| ZH Aeugst a.A.2 | HM468183 | 2B | 2009 | I. ricinus | Switzerland | Gaumann et al. (2010) |
| ZH Bassersdorf1 | HM468184 | 2B | 2009 | I. ricinus | Switzerland | Gaumann et al. (2010) |
| ZH Bassersdorf2 | HM468185 | 2B | 2009 | I. ricinus | Switzerland | Gaumann et al. (2010) |
| AM I | FJ360845 | 2C | 2006 | I. ricinus | Germany: Bavaria | Kupca et al. (2008) |
| AM II | FJ360846 | 2C | 2006 | I. ricinus | Germany: Bavaria | Kupca et al. (2008) |
| AS_1B376-385N_11 | KC154192 | 2C | 2011 | I. ricinus | Germany: South-East Germany, Poppenricht | Frey et al. (2012) |
| AS33 | GQ266392 | 2C | 2005 | I. ricinus | Germany: Amberg | Kupca et al. (2009) |
| SG Moerschwil1 | HM468147 | 2C | 2009 | I. ricinus | Switzerland | Gaumann et al. (2010) |
| SG Moerschwil2 | HM468148 | 2C | 2009 | I. ricinus | Switzerland | Gaumann et al. (2010) |
| T-10 | JQ654645 | 2C | 2006 | Ixodes ricinus | Slovenia | Durmisi et al. (2012) |
| Lithuania-262 | AJ414703 | 2D | 2001 | Human blood | Lithuania | Mickiene et al. (2001) |
| ZH RuetiZH | HM468191 | 2D | 2009 | I. ricinus | Switzerland | Gaumann et al. (2010) |
| SZ Freienbach1 | HM468153 | 2E | 2009 | I. ricinus | Switzerland | Gaumann et al. (2010) |
| SZ Freienbach2 | HM468154 | 2E | 2009 | I. ricinus | Switzerland | Gaumann et al. (2010) |
| H-58 | JQ654665 | 2F | 2009 | Homo sapiens | Slovenia | Durmisi et al. (2012) |
| H-60 | JQ654667 | 2F | 2010 | Homo sapiens | Slovenia | Durmisi et al. (2012) |
| Ljub. I | AF091012 | 2F | 1993 | Human blood | Ljubljana, Slovenia | Ecker et al. (1999) |
| Ljubljana I | JQ654701 | 2F | 1992 | Human serum | Slovenia | Fajs et al. (2012) |
| OW Alpnach | HM468146 | 2F | 2009 | I. ricinus | Switzerland | Gaumann et al. (2010) |
| R-30 | JQ654650 | 2F | 2007 | Myodes glareolus | Slovenia | Durmisi et al. (2012) |
| R-37 | JQ654652 | 2F | 2008 | Myodes glareolus | Slovenia | Durmisi et al. (2012) |
| T-12 | JQ654646 | 2F | 2006 | Ixodes ricinus | Slovenia | Durmisi et al. (2012) |
| T-9 | JQ654644 | 2F | 2006 | Ixodes ricinus | Slovenia | Durmisi et al. (2012) |
| 280 | EF113085 | 2G | 2006 | Unknown | Czech Republic | Ruzek et al. (2006) |
| 282 | EF113087 | 2G | 2006 | Unknown | Czech Republic | Ruzek et al. (2006) |
| Neudoerfl | TEU27495 | 2H | 1971 | I. ricinus | Neudoerfl, Austria | Mandl et al. (1988) |
| TG Lommis2 | HM468159 | 2H | 2009 | I. ricinus | Switzerland | Gaumann et al. (2010) |
| 206 | JF501438 | 2I | 2000 | I. ricinus | Czech Republic: Borovany, South Bohemia | Weidmann et al. (2011) |
| KrM 93 | EU276109 | 2I | 2007 | Tick | South Korea | Yun et.al. (2007) |
| KrM 93 | HM535611 | 2I | 2006 | Apodemus agrarius | South Korea | Yun et al. (2010) |
| Latvia-8110 | AJ319583 | 2J | 2001 | Human blood | Latvia | Lundkvist et al. (2001) |
| Latvia-8369 | AJ319584 | 2J | 2001 | Human blood | Latvia | Lundkvist et al. (2001) |
| 1025 | JF501442 | 2Unique | 2002 | I. ricinus | Czech Republic: Borovany, South Bohemia | Weidmann et al. (2011) |
| 760 | JF501428 | 2Unique | 1997 | I. ricinus | Czech Republic: Neznabohy, North Bohemia | Weidmann et al. (2011) |
| 8641 | JF501406 | 2Unique | 1977 | I. ricinus | Czech Republic: Rabyne, Central Bohemia | Weidmann et al. (2011) |
| 9045 | JF501409 | 2Unique | 1978 | I. ricinus | Czech Republic: Celina, Central Bohemia | Weidmann et al. (2011) |
| AG Brittnau2 | HM468124 | 2Unique | 2009 | I. ricinus | Switzerland | Gaumann et al. (2010) |
| Est3476 | DQ393776 | 2Unique | 2000 | Human blood | Estonia | Golovljova et al. (2004) |
| Est3476 | GU183383 | 2Unique | 2000 | Human TBE case | Estonia | Uzcategui et al. (2012) |
| H-59 | JQ654666 | 2Unique | 2009 | Homo sapiens | Slovenia | Durmisi et al. (2012) |
| H-62 | JQ654657 | 2Unique | 2009 | Homo sapiens | Slovenia | Durmisi et al. (2012) |
| HA_2P1-10N_11 | KC154194 | 2Unique | 2011 | I. ricinus | Germany: South-East Germany, Hauzenberg | Frey et al. (2012) |
| HB_203_11 | KC154196 | 2Unique | 2011 | I. ricinus | Germany: South-East Germany, Heselbach | Frey et al. (2012) |
| HB_215_11 | KC154197 | 2Unique | 2011 | I. ricinus | Germany: South-East Germany, Heselbach | Frey et al. (2012) |
| HB_222_11 | KC154198 | 2Unique | 2011 | I. ricinus | Germany: South-East Germany, Heselbach | Frey et al. (2012) |
| Hypr | TEU39292 | 2Unique | 1953 | Human blood | Brno, Czech Republic | Wallneret al. (1996) |
| Joutseno | GU183381 | 2Unique | 1960 | mouse brain | Finland | Uzcategui et al. (2012) |
| K23 | AF091010 | 2Unique | 1975 | I. ricinus | Karlsruhe, Germany | Ecker et al. (1999) |
| K23 | AM600965 | 2Unique | 1975 | primary chicken embryo cells | Germany:Karlsruhe | Stadler (2007) |
| Kem I | AF091011 | 2Unique | 1952 | I. ricinus | Tatabanya, Hungary | Ecker et al. (1999) |
| KOR-07-046 | FJ972625 | 2Unique | 2007 | Haemaphysalis longicornis nymph | Jeju Island, South Korea | Chae (2009) |
| KrM 213 | EU276110 | 2Unique | 2007 | Tick | South Korea | Yun et.al. (2007) |
| KrM 213 | HM535610 | 2Unique | 2006 | Apodemus agrarius | South Korea | Yun et al. (2010) |
| KrM 215 | EU276111 | 2Unique | 2007 | Tick | South Korea | Yun et.al. (2007) |
| KrM 216 | EU276112 | 2Unique | ? | ? | South Korea | Kim et al. (2006) |
| KrM 219 | EU276113 | 2Unique | ? | ? | South Korea | Kim et al. (2006) |
| LU Ebikon | HM468145 | 2Unique | 2009 | I. ricinus | Switzerland | Gaumann et al. (2010) |
| PA_1R3M_11 | KC154190 | 2Unique | 2011 | I. ricinus | Germany: South-East Germany, Passau | Frey et al. (2012) |
| pool 2201 | KC292217 | 2Unique | 2011 | tick | Germany: Baden-Wuerttemberg | Klaus et al. (2012) |
| pool 2215 | KC292218 | 2Unique | 2011 | tick | Germany: Bavaria | Klaus et al. (2012) |
| R-42 | JQ654653 | 2Unique | 2007 | Apodemus | Slovenia | Durmisi et al. (2012) |
| Stara Ves | AF091018 | 2Unique | Unknown | Unknown | Stara Ves, Croatia | Ecker et al. (1999) |
| T-3 | JQ654642 | 2Unique | 2005 | Ixodes ricinus | Slovenia | Durmisi et al. (2012) |
| T-87 | JF501415 | 2Unique | 1986 | I. ricinus | Czech Republic: Zdar Kaplice, South Bohemia | Weidmann et al. (2011) |
| T-94 | JF501416 | 2Unique | 1986 | I. ricinus | Czech Republic: Hnevkovice, Central Bohemia | Weidmann et al. (2011) |
| V-352 | JF501421 | 2Unique | 1989 | Apodemus sylvaticus | Czech Republic: Zdar Kaplice, South Bohemia | Weidmann et al. (2011) |
| V540 | JF501436 | 2Unique | 1999 | I. ricinus | Czech Republic: Boletice, South Bohemia | Weidmann et al. (2011) |
| ZG Steinhausen3 | HM468180 | 2Unique | 2009 | I. ricinus | Switzerland | Gaumann et al. (2010) |
| ZG Steinhausen4 | HM468181 | 2Unique | 2009 | I. ricinus | Switzerland | Gaumann et al. (2010) |
| ZZ9 | AF091020 | 2Unique | 1985 | I. ricinus | Zell/Ziller, Austria | Ecker et al. (1999) |
| 101 | EU443259 | 3A | 1990 | I. persulcatus | Academgorodok, Novosibirsk, West Siberia, Russia | Tkachev et al. (2008) |
| 1047-10 | KC417478 | 3A | 2010 | I. persulcatus | Russia: Irkutsk region | Adelshin et al. (2012) |
| 1189 | EF469750 | 3A | 1995 | lab mouse brain | Academgorodok, Novosibirsk, West Siberia, Russia | Tkachev et al. (2007) |
| 1203 | JN936392 | 3A | 1996 | I. persulcatus | Novosibirsk region, Western Siberia, Russia | Tkachev et al. (2011) |
| 1386 | JN936395 | 3A | 1997 | I. persulcatus | Novosibirsk region, Western Siberia, Russia | Tkachev et al. (2011) |
| 1427 | EF469754 | 3A | 1998 | lab mouse brain | Novosibirsk region, West Siberia, Russia | Tkachev et al. (2007) |
| 1486 | EF469755 | 3A | 1999 | I. persulcatus | Academgorodok, Novosibirsk, West Siberia, Russia | Tkachev et al. (2007) |
| 1505 | EF469756 | 3A | 1999 | lab mouse brain | Academgorodok, Novosibirsk, West Siberia, Russia | Tkachev et al. (2007) |
| 1526 | EF469757 | 3A | 1999 | I. persulcatus | Academgorodok, Novosibirsk, West Siberia, Russia | Tkachev et al. (2007) |
| 1575 | EF469759 | 3A | 1999 | I. persulcatus | Academgorodok, Novosibirsk, West Siberia, Russia | Tkachev et al. (2007) |
| 1577 | EF469760 | 3A | 1999 | I. persulcatus | Academgorodok, Novosibirsk, West Siberia, Russia | Tkachev et al. (2007) |
| 1587 | EF469761 | 3A | 2000 | I. persulcatus | Academgorodok, Novosibirsk, West Siberia, Russia | Tkachev et al. (2007) |
| 1618 | EU443281 | 3A | 2000 | I. persulcatus | Academgorodok, Novosibirsk, West Siberia, Russia | Tkachev et al. (2008) |
| 1620 | EF469762 | 3A | 2000 | I. persulcatus | Academgorodok, Novosibirsk, West Siberia, Russia | Tkachev et al. (2007) |
| 1627 | EU443282 | 3A | 2000 | I. persulcatus | Academgorodok, Novosibirsk, West Siberia, Russia | Tkachev et al. (2008) |
| 1636 | EU443283 | 3A | 2000 | lab mouse brain | Academgorodok, Novosibirsk, West Siberia, Russia | Tkachev et al. (2008) |
| 1701 | JN936407 | 3A | 2001 | I. persulcatus | Novosibirsk region, Western Siberia, Russia | Tkachev et al. (2011) |
| 1725 | JN936408 | 3A | 2001 | I. persulcatus | Novosibirsk region, Western Siberia, Russia | Tkachev et al. (2011) |
| 1731 | JN936409 | 3A | 2001 | I. persulcatus | Novosibirsk region, Western Siberia, Russia | Tkachev et al. (2011) |
| 1751 | JN936411 | 3A | 2001 | I. persulcatus | Novosibirsk region, Western Siberia, Russia | Tkachev et al. (2011) |
| 1819 | JN936414 | 3A | 2002 | I. persulcatus | Novosibirsk region, Western Siberia, Russia | Tkachev et al. (2011) |
| 237 | EF470570 | 3A | 1988 | I. persulcatus | Academgorodok, Novosibirsk, West Siberia, Russia | Tkachev et al. (2007) |
| 2378 | GQ423567 | 3A | 2007 | lab mouse brain | South-Western Siberia, Novosibirsk, Russia | Morozova et al. (2009) |
| 2421 | GQ423572 | 3A | 2008 | lab mouse brain | South-Western Siberia, Novosibirsk, Russia | Morozova et al. (2009) |
| 2432 | GQ423569 | 3A | 2008 | I. persulcatus | South-Western Siberia, Novosibirsk, Russia | Morozova et al. (2009) |
| 2444 | GQ423571 | 3A | 2008 | lab mouse brain | South-Western Siberia, Novosibirsk, Russia | Morozova et al. (2009) |
| 2453 | GQ423573 | 3A | 2008 | lab mouse brain | South-Western Siberia, Novosibirsk, Russia | Morozova et al. (2009) |
| 2574 | GU060548 | 3A | 2009 | I. persulcatus | South-Western Siberia, Novosibirsk region, Russia | Morozova et al. (2009) |
| 2689 | JQ693478 | 3A | 2010 | ixodid tick | Novosibirsk, Western Siberia, Russia | Tikhomirov et al. (2012) |
| 3 | GU143820 | 3A | 2009 | I. persulcatus | Russia, Ural, Chelyabinsk region | Morozova et al. (2009) |
| 302 | EU443262 | 3A | 1988 | I. persulcatus | Academgorodok, Novosibirsk, West Siberia, Russia | Tkachev et al. (2008) |
| 396 | DQ394880 | 3A | 1981 | I. persulcatus | Academgorodok outskirts, Novosibirsk, West Siberia, Russia | Tkachev et al. (2006) |
| 396 | EF467841 | 3A | 1981 | I. persulcatus | Academgorodok, Novosibirsk, West Siberia, Russia | Tkachev et al. (2007) |
| 834-10 | KC417477 | 3A | 2010 | I. persulcatus | Russia: Irkutsk region | Adelshin et al. (2012) |
| 969 | EU443258 | 3A | 1994 | I. persulcatus | Academgorodok, Novosibirsk, West Siberia, Russia | Tkachev et al. (2008) |
| 983 | EU443265 | 3A | 1994 | I. persulcatus | Academgorodok, Novosibirsk, West Siberia, Russia | Tkachev et al. (2008) |
| Chl11018-2010 | JX315810 | 3A | 2010 | I.persulcatus | Russia, Chelyabinsk region | Kovalev et al. (2012) |
| Chl3247-2005 | GU444175 | 3A | 2005 | I.persulcatus | Russia, Chelyabinsk region | Kovalev et al. (2009) |
| Chl5570-2005 | GU444184 | 3A | 2005 | I.persulcatus | Russia, Chelyabinsk region | Kovalev et al. (2009) |
| Ekaterinburg-338-09 | GQ845429 | 3A | 2009 | I. persulcatus | Ural, Sverdlovsk region | Karan et al. (2009) |
| Ekaterinburg-35-8-06 | FJ214122 | 3A | 2006 | I. persulcatus | Ekaterinburg, Ural, Russia | Karan et al. (2008) |
| Ekaterinburg-37-3-06 | FJ214126 | 3A | 2006 | I. persulcatus | Ekaterinburg, Ural, Russia | Karan et al. (2008) |
| Ekaterinburg-421-09 | GQ845421 | 3A | 2009 | I. persulcatus | Ural, Sverdlovsk region, Serov city | Karan et al. (2009) |
| Ekaterinburg-439-09 | GQ845425 | 3A | 2009 | I. persulcatus | Ural, Sverdlovsk region | Karan et al. (2009) |
| Ekaterinburg-44-2-06 | FJ214124 | 3A | 2006 | I. persulcatus | Ekaterinburg, Ural, Russia | Karan et al. (2008) |
| Ekaterinburg-56-03 | FJ214127 | 3A | 2003 | I. persulcatus | Ekaterinburg, Ural, Russia | Karan et al. (2008) |
| Ekaterinburg-612-09 | GQ845420 | 3A | 2009 | I. persulcatus | Ural, Sverdlovsk region, Ekaterinburg, Chusovskoy lake | Karan et al. (2009) |
| Ekaterinburg-716-09 | GQ845428 | 3A | 2009 | I. persulcatus | Ural, Sverdlovsk region, Shalinsk, Shabri | Karan et al. (2009) |
| Ekaterinburg-859-09 | GQ845427 | 3A | 2009 | I. persulcatus | Ural, Sverdlovsk region, Nizhneserginsk, Ilmovka | Karan et al. (2009) |
| Ekb1031-2008 | GU444262 | 3A | 2008 | I.persulcatus | Russia: Ural, Sverdlovsk region, Verkhotur'e district | Kovalev et al. (2009) |
| Ekb104-2009 | JX315990 | 3A | 2009 | I.persulcatus | Russia: Alapaevsk, Sverdlovsk region | Kovalev et al. (2012) |
| Ekb106-2009 | JX315976 | 3A | 2009 | I.persulcatus | Russia: Krasnoturinsk, Sverdlovsk region | Kovalev et al. (2012) |
| Ekb1066-2009 | JX315987 | 3A | 2009 | I.persulcatus | Russia: Alapaevsk district, Sverdlovsk region | Kovalev et al. (2012) |
| Ekb1067-2009 | JX315988 | 3A | 2009 | I.persulcatus | Russia: Alapaevsk district, Sverdlovsk region | Kovalev et al. (2012) |
| Ekb108-2009 | JX315989 | 3A | 2009 | I.persulcatus | Russia: Yekaterinburg, Sverdlovsk region | Kovalev et al. (2012) |
| Ekb1087-2010 | JX315808 | 3A | 2010 | I.persulcatus | Russia: Sverdlovsk region + | Kovalev et al. (2012) |
| Ekb11008-2010 | JX315809 | 3A | 2010 | I.persulcatus | Russia: Yekaterinburg, Sverdlovsk region | Kovalev et al. (2012) |
| Ekb110-2008 | GU444259 | 3A | 2008 | I.persulcatus | Russia: Ural, Sverdlovsk region, Sysert' district | Kovalev et al. (2009) |
| Ekb11094-2010 | JX315811 | 3A | 2010 | I.persulcatus | Russia: Sverdlovsk region, Yekaterinburg | Kovalev et al. (2012) |
| Ekb11113-2010 | JX315812 | 3A | 2010 | I.persulcatus | Russia: Nizhnye Ser'gy, Sverdlovsk region | Kovalev et al. (2012) |
| Ekb11-2007 | GU444249 | 3A | 2007 | I.persulcatus | Russia: Ural, Sverdlovsk region, Nyzhnyi Tagil district | Kovalev et al. (2009) |
| Ekb11413-2010 | JX315813 | 3A | 2010 | I.persulcatus | Russia: Beloyarskdistrict, Sverdlovsk region | Kovalev et al. (2012) |
| Ekb1149-2008 | GU444269 | 3A | 2008 | I.persulcatus | Russia: Ural, Sverdlovsk region, Serov district | Kovalev et al. (2009) |
| Ekb1151-2008 | GU444270 | 3A | 2008 | I.persulcatus | Russia: Ural, Sverdlovsk region, Serov district | Kovalev et al. (2009) |
| Ekb1153-2008 | GU444271 | 3A | 2008 | I.persulcatus | Russia: Ural, Sverdlovsk region, Serov district | Kovalev et al. (2009) |
| Ekb1160-2008 | GU444272 | 3A | 2008 | I.persulcatus | Russia: Ural, Sverdlovsk region, Serov district | Kovalev et al. (2009) |
| Ekb1163-2008 | GU444273 | 3A | 2008 | I.persulcatus | Russia: Ural, Sverdlovsk region, Serov district | Kovalev et al. (2009) |
| Ekb11706-2010 | JX315814 | 3A | 2010 | I.persulcatus | Russia: Sverdlovsk region | Kovalev et al. (2012) |
| Ekb118-2008 | GU444260 | 3A | 2008 | I.persulcatus | Russia: Ural, Sverdlovsk region, Revda district | Kovalev et al. (2009) |
| Ekb1192-2007 | JX315776 | 3A | 2007 | I.persulcatus | Russia: Sukhoi Log district, Sverdlovsk region | Kovalev et al. (2012) |
| Ekb1194-2010 | JX315816 | 3A | 2010 | I.persulcatus | Russia: Sverdlovsk region | Kovalev et al. (2012) |
| Ekb1198-2010 | JX315817 | 3A | 2010 | I.persulcatus | Russia: Sverdlovsk region | Kovalev et al. (2012) |
| Ekb12046-2011 | JX315904 | 3A | 2011 | I.persulcatus | Russia: Yekaterinburg, Sverdlovsk region | Kovalev et al. (2012) |
| Ekb12-13-2006 | GU444197 | 3A | 2006 | I.persulcatus | Russia: Ural, Sverdlovsk region, Sysert' district | Kovalev et al. (2009) |
| Ekb12-16-2006 | GU444198 | 3A | 2006 | I.persulcatus | Russia: Ural, Sverdlovsk region, Sysert' district | Kovalev et al. (2009) |
| Ekb12-17-2006 | GU444199 | 3A | 2006 | I.persulcatus | Russia: Ural, Sverdlovsk region, Krasnoturinsk district | Kovalev et al. (2009) |
| Ekb1219-2010 | JX315818 | 3A | 2010 | I.persulcatus | Russia: Sverdlovsk region | Kovalev et al. (2012) |
| Ekb12-2007 | GU444250 | 3A | 2007 | I.persulcatus | Russia: Ural, Sverdlovsk region, Nyzhnyi Tagil district | Kovalev et al. (2009) |
| Ekb12301-2011 | JX315907 | 3A | 2011 | I.persulcatus | Russia: Verkhnyaya Pyshma district, Sverdlovsk region | Kovalev et al. (2012) |
| Ekb12466-2011 | JX315948 | 3A | 2011 | I.persulcatus | Russia: Revda district, Sverdlovsk region | Kovalev et al. (2012) |
| Ekb125-2008 | GU444261 | 3A | 2008 | I.persulcatus | Russia: Ural, Sverdlovsk region, Sysert' district | Kovalev et al. (2009) |
| Ekb125-2010 | JX315820 | 3A | 2010 | I.persulcatus | Russia: Asbest district, Sverdlovsk region | Kovalev et al. (2012) |
| Ekb13-2007 | GU444251 | 3A | 2007 | I.persulcatus | Russia: Ural, Sverdlovsk region, Nyzhnyi Tagil district | Kovalev et al. (2009) |
| Ekb13-23-2006 | GU444201 | 3A | 2006 | I.persulcatus | Russia: Ural, Sverdlovsk region, Polevskoi district | Kovalev et al. (2009) |
| Ekb13-23-2006 | JX315759 | 3A | 2006 | I.persulcatus | Russia: Polevskoi, Sverdlovsk region | Kovalev et al. (2012) |
| Ekb135-2012 | KC886325 | 3A | 2012 | I.persulcatus | Russia: Sverdlovsk region, Asbest | Kovalev et al. (2013) |
| Ekb1365-1978 | GU444150 | 3A | 1978 | I.persulcatus | Russia: Ural, Sverdlovsk region, Nizhnye Sergi district | Kovalev et al. (2009) |
| Ekb13-8-2006 | GU444200 | 3A | 2006 | I.persulcatus | Russia: Ural, Sverdlovsk region, Sysert' district | Kovalev et al. (2009) |
| Ekb14480-2011 | JX315905 | 3A | 2011 | I.persulcatus | Russia: Sysert' district, Sverdlovsk region | Kovalev et al. (2012) |
| Ekb1449-1969 | GU444151 | 3A | 1969 | I.persulcatus | Russia: Ural, Sverdlovsk region, Pyshma district | Kovalev et al. (2009) |
| Ekb1466-2012 | KC886326 | 3A | 2012 | I.persulcatus | Russia: Sverdlovsk region | Kovalev et al. (2013) |
| Ekb15023-2012 | KC886327 | 3A | 2012 | I.persulcatus | Russia: Sverdlovsk region | Kovalev et al. (2013) |
| Ekb1524-1975 | GU444126 | 3A | 1975 | I.persulcatus | Russia: Ural, Sverdlovsk region, Pervoural'sk district | Kovalev et al. (2009) |
| Ekb158-2010 | JX315825 | 3A | 2010 | I.persulcatus | Russia: Sysert' district, Sverdlovsk region | Kovalev et al. (2012) |
| Ekb159-2010 | JX315826 | 3A | 2010 | I.persulcatus | Russia: Sysert' district, Sverdlovsk region | Kovalev et al. (2012) |
| Ekb15941-2011 | JX315906 | 3A | 2011 | I.persulcatus | Russia: Kamensk, Sverdlovsk region | Kovalev et al. (2012) |
| Ekb1627-1969 | GU444155 | 3A | 1969 | I.persulcatus | Russia: Ural, Sverdlovsk region, Shalya | Kovalev et al. (2009) |
| Ekb1653-1969 | GU444130 | 3A | 1969 | I.persulcatus | Russia: Ural, Sverdlovsk region, Talitsa district | Kovalev et al. (2009) |
| Ekb1656-4-1969 | JX315723 | 3A | 1969 | I.persulcatus | Russia: Talitsa, Sverdlovsk region | Kovalev et al. (2012) |
| Ekb1687-5-1969 | JX315724 | 3A | 1969 | I.persulcatus | Russia: Pyshma district, Sverdlovsk region | Kovalev et al. (2012) |
| Ekb173-2011 | JX315927 | 3A | 2011 | I.persulcatus | Russia: Arti district, Sverdlovsk region | Kovalev et al. (2012) |
| Ekb175-2011 | JX315928 | 3A | 2011 | I.persulcatus | Russia: Arti district, Sverdlovsk region | Kovalev et al. (2012) |
| Ekb17605-2011 | JX315940 | 3A | 2011 | I.persulcatus | Russia: Sysert' district, Sverdlovsk region | Kovalev et al. (2012) |
| Ekb176-1-2011 | JX315945 | 3A | 2011 | I.persulcatus | Russia: Sukhoi Log district, Sverdlovsk region | Kovalev et al. (2012) |
| Ekb176-2-2011 | JX315946 | 3A | 2011 | I.persulcatus | Russia: Sukhoi Log, Sverdlovsk region | Kovalev et al. (2012) |
| Ekb17663-2011 | JX315931 | 3A | 2011 | I.persulcatus | Russia: Polevskoi, Sverdlovsk region | Kovalev et al. (2012) |
| Ekb17-7-2006 | GU444202 | 3A | 2006 | I.persulcatus | Russia: Ural, Sverdlovsk region, Sysert' district | Kovalev et al. (2009) |
| Ekb177-2011 | JX315947 | 3A | 2011 | I.persulcatus | Russia: Sukhoi Log district, Sverdlovsk region | Kovalev et al. (2012) |
| Ekb177-2012 | KC886329 | 3A | 2012 | I.persulcatus | Russia: Sverdlovsk region, Beryozovskyi district | Kovalev et al. (2013) |
| Ekb178-2011 | JX315929 | 3A | 2011 | I.persulcatus | Russia: Bogdanovich district, Sverdlovsk region | Kovalev et al. (2012) |
| Ekb182-2008 | GU444282 | 3A | 2008 | Human blood | Russia: Ural, Sverdlovsk region, Alapaevsk | Kovalev et al. (2009) |
| Ekb183-2007 | GU444241 | 3A | 2007 | I.persulcatus | Russia: Ural, Sverdlovsk region, Pervoural'sk district | Kovalev et al. (2009) |
| Ekb184-2009 | JX315967 | 3A | 2009 | I.persulcatus | Russia: Yekaterinburg, Sverdlovsk region | Kovalev et al. (2012) |
| Ekb1842-2006 | GU444214 | 3A | 2006 | I.persulcatus | Russia: Ural, Sverdlovsk region, Beryozovsk | Kovalev et al. (2009) |
| Ekb1874-2006 | GU444215 | 3A | 2006 | I.persulcatus | Russia: Ural, Sverdlovsk region, Yekaterinburg district | Kovalev et al. (2009) |
| Ekb1900-2010 | JX315828 | 3A | 2010 | I.persulcatus | Russia: Yekaterinburg, Sverdlovsk region | Kovalev et al. (2012) |
| Ekb1928-2010 | JX315829 | 3A | 2010 | I.persulcatus | Russia: Sverdlovsk region | Kovalev et al. (2012) |
| Ekb193-2007 | GU444226 | 3A | 2007 | I.persulcatus | Russia: Ural, Sverdlovsk region, Bogdanovich district | Kovalev et al. (2009) |
| Ekb1935-2010 | JX315830 | 3A | 2010 | I.persulcatus | Russia: Sverdlovsk region | Kovalev et al. (2012) |
| Ekb1987-2007 | GU444221 | 3A | 2007 | I.persulcatus | Russia: Ural, Sverdlovsk region, Karpinsk district | Kovalev et al. (2009) |
| Ekb200505-2005 | GU444186 | 3A | 2005 | I.persulcatus | Russia: Ural, Sverdlovsk region, Sysert' district | Kovalev et al. (2009) |
| Ekb2012-2007 | GU444252 | 3A | 2007 | I.persulcatus | Russia: Ural, Sverdlovsk region, Revda district | Kovalev et al. (2009) |
| Ekb2016-2009 | JX315969 | 3A | 2009 | I.persulcatus | Russia: Sverdlovsk region + | Kovalev et al. (2012) |
| Ekb2017-1969 | JX315725 | 3A | 1969 | Human blood | Russia: Tugulym, Sverdlovsk region | Kovalev et al. (2012) |
| Ekb205-2009 | JX315962 | 3A | 2009 | I.persulcatus | Russia: Yekaterinburg, Sverdlovsk region | Kovalev et al. (2012) |
| Ekb21011-2009 | JX315973 | 3A | 2009 | I.persulcatus | Russia: Sverdlovsk region + | Kovalev et al. (2012) |
| Ekb21012-2009 | JX315974 | 3A | 2009 | I.persulcatus | Russia: Sverdlovsk region + | Kovalev et al. (2012) |
| Ekb2122-2005 | GU444167 | 3A | 2005 | I.persulcatus | Russia: Ural, Sverdlovsk region | Kovalev et al. (2009) |
| Ekb213-2012 | KC886330 | 3A | 2012 | I.persulcatus | Russia: Sverdlovsk region, Sysert' district | Kovalev et al. (2013) |
| Ekb2231-1969 | GU444131 | 3A | 1969 | Human blood | Russia: Ural, Sverdlovsk region, Shalya district | Kovalev et al. (2009) |
| Ekb2338-2009 | JX315978 | 3A | 2009 | I.persulcatus | Russia: Sverdlovsk region + | Kovalev et al. (2012) |
| Ekb237-2011 | JX315911 | 3A | 2011 | I.persulcatus | Russia: Serov district, Sverdlovsk region | Kovalev et al. (2012) |
| Ekb2439-2009 | JX315968 | 3A | 2009 | I.persulcatus | Russia: Sverdlovsk region + | Kovalev et al. (2012) |
| Ekb2500-2005 | GU444169 | 3A | 2005 | I.persulcatus | Russia: Ural, Sverdlovsk region, Yekaterinburg district | Kovalev et al. (2009) |
| Ekb2517-2005 | GU444170 | 3A | 2005 | I.persulcatus | Russia: Ural, Sverdlovsk region | Kovalev et al. (2009) |
| Ekb2519-2005 | GU444171 | 3A | 2005 | I.persulcatus | Russia: Ural, Sverdlovsk region, Sysert' district | Kovalev et al. (2009) |
| Ekb2593-2005 | GU444172 | 3A | 2005 | I.persulcatus | Russia: Ural, Sverdlovsk region, Yekaterinburg district | Kovalev et al. (2009) |
| Ekb2681-2005 | GU444173 | 3A | 2005 | I.persulcatus | Russia: Ural, Sverdlovsk region, Yekaterinburg district | Kovalev et al. (2009) |
| Ekb270-2009 | JX315971 | 3A | 2009 | I.persulcatus | Russia: Sysert', Sverdlovsk region | Kovalev et al. (2012) |
| Ekb27-1-2007 | JX315766 | 3A | 2007 | I.persulcatus | Russia: Polevskoi district, Sverdlovsk region | Kovalev et al. (2012) |
| Ekb276-2012 | KC886331 | 3A | 2012 | I.persulcatus | Russia: Sverdlovsk region, Krasnouralsk district | Kovalev et al. (2013) |
| Ekb2880-2005 | GU444174 | 3A | 2005 | I.persulcatus | Russia: Ural, Sverdlovsk region, Nev'yansk district | Kovalev et al. (2009) |
| Ekb302-2008 | GU444281 | 3A | 2008 | I.persulcatus | Russia: Ural, Sverdlovsk region, Kushva district | Kovalev et al. (2009) |
| Ekb3039-2010 | JX315845 | 3A | 2010 | I.persulcatus | Russia: Sverdlovsk region | Kovalev et al. (2012) |
| Ekb3040-2010 | JX315846 | 3A | 2010 | I.persulcatus | Russia: Sverdlovsk region | Kovalev et al. (2012) |
| Ekb304-2009 | JX315970 | 3A | 2009 | I.persulcatus | Russia: Serov, Sverdlovsk region | Kovalev et al. (2012) |
| Ekb307-2009 | JX315961 | 3A | 2009 | I.persulcatus | Russia: Serov, Sverdlovsk region | Kovalev et al. (2012) |
| Ekb308-2009 | JX315998 | 3A | 2009 | I.persulcatus | Russia: Serov, Sverdlovsk region | Kovalev et al. (2012) |
| Ekb312-2005 | GU444164 | 3A | 2005 | I.persulcatus | Russia: Ural, Sverdlovsk region, Yekaterinburg district | Kovalev et al. (2009) |
| Ekb312-2009 | JX315965 | 3A | 2009 | I.persulcatus | Russia: Beryozovskyi, Sverdlovsk region | Kovalev et al. (2012) |
| Ekb314-2010 | JX315850 | 3A | 2010 | I.persulcatus | Russia: Severouralsk, Sverdlovsk region | Kovalev et al. (2012) |
| Ekb3174-2009 | JX315963 | 3A | 2009 | I.persulcatus | Russia: Sverdlovsk region | Kovalev et al. (2012) |
| Ekb322-2008 | GU444278 | 3A | 2008 | I.persulcatus | Russia: Ural, Sverdlovsk region, Asbest district | Kovalev et al. (2009) |
| Ekb322-2011 | JX315941 | 3A | 2011 | I.persulcatus | Russia: Sysert' district, Sverdlovsk region | Kovalev et al. (2012) |
| Ekb33-2007 | GU444232 | 3A | 2007 | I.persulcatus | Russia: Ural, Sverdlovsk region, Polevskoi district | Kovalev et al. (2009) |
| Ekb346-2006 | GU444203 | 3A | 2006 | I.persulcatus | Russia: Ural, Sverdlovsk region, Rezh district | Kovalev et al. (2009) |
| Ekb346-2009 | JX315997 | 3A | 2009 | I.persulcatus | Russia: Revda, Sverdlovsk region | Kovalev et al. (2012) |
| Ekb347-1978 | GU444157 | 3A | 1978 | I.persulcatus | Russia: Ural, Sverdlovsk region, Baikalovo district | Kovalev et al. (2009) |
| Ekb3495-1984 | GU444128 | 3A | 1984 | I.persulcatus | Russia: Ural, Sverdlovsk region, Nyzhye Sergi district | Kovalev et al. (2009) |
| Ekb357-2005 | GU339055 | 3A | 2005 | I.persulcatus | Russia: Ural, Sverdlovsk region, Sysert | Kovalev et al. (2009) |
| Ekb3590-1978 | GU444133 | 3A | 1978 | Human blood | Russia: Ural, Sverdlovsk region, Yekaterinburg district | Kovalev et al. (2009) |
| Ekb360-2012 | KC886334 | 3A | 2012 | I.persulcatus | Russia: Sverdlovsk region, Serov district | Kovalev et al. (2013) |
| Ekb361-2012 | KC886335 | 3A | 2012 | I.persulcatus | Russia: Sverdlovsk region, Serov district | Kovalev et al. (2013) |
| Ekb370-2010 | JX315852 | 3A | 2010 | I.persulcatus | Russia: Verkhnyaya Salda, Sverdlovsk region | Kovalev et al. (2012) |
| Ekb371-2010 | JX315853 | 3A | 2010 | I.persulcatus | Russia: Verkhnyaya Salda, Sverdlovsk region | Kovalev et al. (2012) |
| Ekb3718-1977 | GU444134 | 3A | 1977 | I.persulcatus | Russia: Ural, Sverdlovsk region, Serov district | Kovalev et al. (2009) |
| Ekb37-1969 | GU444145 | 3A | 1969 | I.persulcatus | Russia: Ural, Sverdlovsk region, Nizhnyi Tagil district | Kovalev et al. (2009) |
| Ekb3745-1968 | JX315719 | 3A | 1968 | Human blood | Russia: Nizhnyi Tagil, Sverdlovsk region | Kovalev et al. (2012) |
| Ekb382-2009 | JX315999 | 3A | 2009 | I.persulcatus | Russia: Serov, Sverdlovsk region | Kovalev et al. (2012) |
| Ekb4083-1978 | GU444135 | 3A | 1978 | I.persulcatus | Russia: Ural, Sverdlovsk region, Baikalovo district | Kovalev et al. (2009) |
| Ekb41-00 | GU444124 | 3A | ? | Unknown | Russia: Ural, Sverdlovsk region | Kovalev et al. (2009) |
| Ekb410-2011 | JX315938 | 3A | 2011 | I.persulcatus | Russia: Karpinsk district, Sverdlovsk region | Kovalev et al. (2012) |
| Ekb41-2007 | JX315768 | 3A | 2007 | I.persulcatus | Russia: Polevskoi district, Sverdlovsk region | Kovalev et al. (2012) |
| Ekb42-2008 | GU444257 | 3A | 2008 | I.persulcatus | Russia: Ural, Sverdlovsk region, Polevskoi | Kovalev et al. (2009) |
| Ekb4299-2011 | JX315954 | 3A | 2011 | I.persulcatus | Russia: Sverdlovsk region | Kovalev et al. (2012) |
| Ekb43-2010 | JX315875 | 3A | 2010 | I.persulcatus | Russia: Yekaterinburg, Sverdlovsk region | Kovalev et al. (2012) |
| Ekb4337-2011 | JX315930 | 3A | 2011 | I.persulcatus | Russia: Sverdlovsk region | Kovalev et al. (2012) |
| Ekb4397-2011 | JX315917 | 3A | 2011 | I.persulcatus | Russia: Sverdlovsk region | Kovalev et al. (2012) |
| Ekb443-2011 | JX315919 | 3A | 2011 | I.persulcatus | Russia: Beryozovskyi district, Sverdlovsk region | Kovalev et al. (2012) |
| Ekb4458-2005 | GU444176 | 3A | 2005 | I.persulcatus | Russia: Ural, Sverdlovsk region, Beloyarsk district | Kovalev et al. (2009) |
| Ekb4469-2005 | GU444178 | 3A | 2005 | I.persulcatus | Russia: Ural, Sverdlovsk region | Kovalev et al. (2009) |
| Ekb4471-2011 | JX315932 | 3A | 2011 | I.persulcatus | Russia: Sverdlovsk region | Kovalev et al. (2012) |
| Ekb4630-1978 | GU444125 | 3A | 1978 | Human blood | Russia: Ural, Sverdlovsk region, Yekaterinburg district | Kovalev et al. (2009) |
| Ekb47-2012 | KC886336 | 3A | 2012 | I.persulcatus | Russia: Sverdlovsk region, Yekaterinburg | Kovalev et al. (2013) |
| Ekb4801-2011 | JX315901 | 3A | 2011 | I.persulcatus | Russia: Sverdlovsk region | Kovalev et al. (2012) |
| Ekb493-2011 | JX315950 | 3A | 2011 | I.persulcatus | Russia: Sverdlovsk region | Kovalev et al. (2012) |
| Ekb4981-2011 | JX315918 | 3A | 2011 | I.persulcatus | Russia: Sverdlovsk region | Kovalev et al. (2012) |
| Ekb5045-2005 | GU444179 | 3A | 2005 | I.persulcatus | Russia: Ural, Sverdlovsk region, Ber'yozovsk district | Kovalev et al. (2009) |
| Ekb51-1968 | JX315720 | 3A | 1968 | I.persulcatus | Russia: Nizhnyi Tagil, Sverdlovsk region | Kovalev et al. (2012) |
| Ekb51-2012 | KC886337 | 3A | 2012 | I.persulcatus | Russia: Sverdlovsk region, Yekaterinburg | Kovalev et al. (2013) |
| Ekb51-2-1968 | JX315721 | 3A | 1968 | I.persulcatus | Russia: Nizhnyi Tagil, Sverdlovsk region | Kovalev et al. (2012) |
| Ekb5191-2005 | GU444180 | 3A | 2005 | I.persulcatus | Russia: Ural, Sverdlovsk region, Lyalya | Kovalev et al. (2009) |
| Ekb52-2011 | JX315949 | 3A | 2011 | I.persulcatus | Russia: Yekaterinburg, Sverdlovsk region | Kovalev et al. (2012) |
| Ekb53-2012 | KC886339 | 3A | 2012 | I.persulcatus | Russia: Sverdlovsk region, Yekaterinburg | Kovalev et al. (2013) |
| Ekb5357-2005 | GU444182 | 3A | 2005 | I.persulcatus | Russia: Ural, Sverdlovsk region, Ber'yozovsk district | Kovalev et al. (2009) |
| Ekb54-1-ENIIVI | GU444123 | 3A | ? | Unknown | Russia: Ural, Sverdlovsk region | Kovalev et al. (2009) |
| Ekb54-2012 | KC886340 | 3A | 2012 | I.persulcatus | Russia: Sverdlovsk region, Yekaterinburg | Kovalev et al. (2013) |
| Ekb54-2-ENIIVI | GU444122 | 3A | ? | Unknown | Russia: Ural, Sverdlovsk region | Kovalev et al. (2009) |
| Ekb5465-2005 | GU444188 | 3A | 2005 | I.persulcatus | Russia: Ural, Sverdlovsk region, Beloyarsk district | Kovalev et al. (2009) |
| Ekb55-2008 | GU444258 | 3A | 2008 | I.persulcatus | Russia: Ural, Sverdlovsk region | Kovalev et al. (2009) |
| Ekb5571-2005 | GU444189 | 3A | 2005 | I.persulcatus | Russia: Ural, Sverdlovsk region,Yekaterinburg district | Kovalev et al. (2009) |
| Ekb56-2012 | KC886341 | 3A | 2012 | I.persulcatus | Russia: Sverdlovsk region, Yekaterinburg | Kovalev et al. (2013) |
| Ekb5632-2011 | JX315914 | 3A | 2011 | I.persulcatus | Russia: Sverdlovsk region | Kovalev et al. (2012) |
| Ekb58-2010 | JX315876 | 3A | 2010 | I.persulcatus | Russia: Kamensk, Sverdlovsk region | Kovalev et al. (2012) |
| Ekb611-2012 | KC886343 | 3A | 2012 | I.persulcatus | Russia: Sverdlovsk region, Nizhnyi Tagil district | Kovalev et al. (2013) |
| Ekb613-2010 | JX315878 | 3A | 2010 | I.persulcatus | Russia: Nizhnyi Tagil, Sverdlovsk region | Kovalev et al. (2012) |
| Ekb623-2012 | KC886344 | 3A | 2012 | I.persulcatus | Russia: Sverdlovsk region, Nizhnyi Tagil district | Kovalev et al. (2013) |
| Ekb629-2012 | KC886345 | 3A | 2012 | I.persulcatus | Russia: Sverdlovsk region, Nizhnyi Tagil district | Kovalev et al. (2013) |
| Ekb631-2012 | KC886346 | 3A | 2012 | I.persulcatus | Russia: Sverdlovsk region, Nizhnyi Tagil district | Kovalev et al. (2013) |
| Ekb698-2007 | GU444228 | 3A | 2007 | I.persulcatus | Russia: Ural, Sverdlovsk region, Sysert' district | Kovalev et al. (2009) |
| Ekb7014-2009 | JX315964 | 3A | 2009 | Human brain | Russia: Nizhnyi Tagil, Sverdlovsk region | Kovalev et al. (2012) |
| Ekb704-2006 | JX315758 | 3A | 2006 | I.persulcatus | Russia: Beryozovsk district, Sverdlovsk region | Kovalev et al. (2012) |
| Ekb7-2007 | GU444245 | 3A | 2007 | I.persulcatus | Russia: Ural, Sverdlovsk region, Nyzhnyi Tagil | Kovalev et al. (2009) |
| Ekb78-2007 | GU444239 | 3A | 2007 | I.persulcatus | Russia: Ural, Sverdlovsk region, Polevskoi district | Kovalev et al. (2009) |
| Ekb785-2006 | GU444205 | 3A | 2006 | I.persulcatus | Russia: Ural, Sverdlovsk region, Beryozovsk district | Kovalev et al. (2009) |
| Ekb80-1969 | GU444154 | 3A | 1969 | I.persulcatus | Russia: Ural, Sverdlovsk region, Nizhnyi Tagil district | Kovalev et al. (2009) |
| Ekb8-2007 | GU444246 | 3A | 2007 | I.persulcatus | Russia: Ural, Sverdlovsk region, Nyzhnyi Tagil district | Kovalev et al. (2009) |
| Ekb862-2005 | GU444165 | 3A | 2005 | I.persulcatus | Russia: Ural, Sverdlovsk region, Ber'yozovsk district | Kovalev et al. (2009) |
| Ekb862-2010 | JX315884 | 3A | 2010 | I.persulcatus | Russia: Sysert' District, Sverdlovsk region | Kovalev et al. (2012) |
| Ekb867-2007 | GU444229 | 3A | 2007 | I.persulcatus | Russia: Ural, Sverdlovsk region, Yekaterinburg district | Kovalev et al. (2009) |
| Ekb885-2005 | GU444166 | 3A | 2005 | I.persulcatus | Russia: Ural, Sverdlovsk region, Nizhnie Sergi district | Kovalev et al. (2009) |
| Ekb888-2006 | GU444208 | 3A | 2006 | I.persulcatus | Russia: Ural, Sverdlovsk region, Rezh district | Kovalev et al. (2009) |
| Ekb89-2011 | JX315939 | 3A | 2011 | I.persulcatus | Russia: Kamensk, Sverdlovsk region | Kovalev et al. (2012) |
| Ekb9130118-2012 | KC886348 | 3A | 2012 | I.persulcatus | Russia: Sverdlovsk region, Kamensk-Ural'sk district | Kovalev et al. (2013) |
| Ekb9300466-2012 | KC886350 | 3A | 2012 | I.persulcatus | Russia: Sverdlovsk region, Yekaterinburg | Kovalev et al. (2013) |
| Ekb93-2010 | JX315886 | 3A | 2010 | I.persulcatus | Russia: Yekaterinburg, Sverdlovsk region | Kovalev et al. (2012) |
| Ekb95-2012 | KC886351 | 3A | 2012 | I.persulcatus | Russia: Sverdlovsk region, Polevskoi district | Kovalev et al. (2013) |
| Ekb96-2012 | KC886352 | 3A | 2012 | I.persulcatus | Russia: Sverdlovsk region, Polevskoi district | Kovalev et al. (2013) |
| EkbEgoshin-2009 | JX315966 | 3A | 2009 | Human brain | Russia: Rezh, Sverdlovsk region | Kovalev et al. (2012) |
| EkbZhigalina-2009 | JX315995 | 3A | 2009 | Human brain | Russia: Tavda, Sverdlovsk region | Kovalev et al. (2012) |
| IR99-2f7 | AB049352 | 3A | 1999 | I. persulcatus | Irkutsk, Russia | Hayasaka et al. (2001) |
| IR99-2m7 | AB049351 | 3A | 1999 | I. persulcatus | Irkutsk, Russia | Hayasaka et al. (2001) |
| Kemerovo-134-08 | GQ845433 | 3A | 2008 | I. persulcatus | Russia, West Siberia, Kemerovo region, Prokopyevsk, Chistugash | Karan et al. (2009) |
| Kemerovo-167 | GQ845431 | 3A | 2008 | I. persulcatus | Russia, West Siberia, Kemerovo region, Prokopyevsk, Chistugash | Karan et al. (2009) |
| Krg4023-2008 | JX315788 | 3A | 2008 | I.persulcatus | Russia, Kurgan region | Kovalev et al. (2012) |
| Kurgan-16-09 | GQ845426 | 3A | 2009 | I. persulcatus | Ural, Kurgan region, Kataysk | Karan et al. (2009) |
| Kurgan-279-07 | FJ214149 | 3A | 2007 | I. persulcatus | Kurgan, Ural, Russia | Karan et al. (2008) |
| Kurgan-280-07 | FJ214152 | 3A | 2007 | I. persulcatus | Kurgan, Ural, Russia | Karan et al. (2008) |
| Kurgan-316-07 | FJ214151 | 3A | 2007 | I. persulcatus | Kurgan, Ural, Russia | Karan et al. (2008) |
| Kurgan-371-07 | FJ214150 | 3A | 2007 | I. persulcatus | Kurgan, Ural, Russia | Karan et al. (2008) |
| Kurgan-373-09 | GQ845417 | 3A | 2009 | I. persulcatus | Ural, Kurgan region | Karan et al. (2009) |
| Omsk4009-2010 | JX315861 | 3A | 2010 | I.persulcatus | Russia, Omsk region | Kovalev et al. (2012) |
| Omsk4036-2010 | JX315865 | 3A | 2010 | I.persulcatus | Russia, Omsk region | Kovalev et al. (2012) |
| Omsk4044-2008 | JX315797 | 3A | 2008 | I.persulcatus | Russia, Omsk region | Kovalev et al. (2012) |
| Omsk4048-2008 | JX315798 | 3A | 2008 | I.persulcatus | Russia, Omsk region | Kovalev et al. (2012) |
| Omsk4049-2008 | JX315801 | 3A | 2008 | I.persulcatus | Russia, Omsk region | Kovalev et al. (2012) |
| Omsk4051-2008 | JX315799 | 3A | 2008 | I.persulcatus | Russia, Omsk region | Kovalev et al. (2012) |
| Omsk4052-2008 | JX315803 | 3A | 2008 | I.persulcatus | Russia, Omsk region | Kovalev et al. (2012) |
| Omsk4056-2010 | JX315868 | 3A | 2010 | I.persulcatus | Russia, Omsk region | Kovalev et al. (2012) |
| Omsk4066-2010 | JX315869 | 3A | 2010 | I.persulcatus | Russia, Omsk region | Kovalev et al. (2012) |
| Omsk4075-2010 | JX315873 | 3A | 2010 | I.persulcatus | Russia, Omsk region | Kovalev et al. (2012) |
| Perm7584-2011 | JX315903 | 3A | 2011 | I.persulcatus | Russia, Perm' region | Kovalev et al. (2012) |
| Prm72884-2011 | JX315910 | 3A | 2011 | I.persulcatus | Russia, Perm' region | Kovalev et al. (2012) |
| Prm72913-2011 | JX315935 | 3A | 2011 | I.persulcatus | Russia, Perm' region | Kovalev et al. (2012) |
| Prm73275-2011 | JX315937 | 3A | 2011 | I.persulcatus | Russia, Perm' region | Kovalev et al. (2012) |
| Prm74051-2011 | JX315921 | 3A | 2011 | I.persulcatus | Russia, Perm' region | Kovalev et al. (2012) |
| Prm74305-2011 | JX315955 | 3A | 2011 | I.persulcatus | Russia, Perm' region | Kovalev et al. (2012) |
| Prm74373-2011 | JX315915 | 3A | 2011 | I.persulcatus | Russia, Perm' region | Kovalev et al. (2012) |
| Prm74500-2011 | JX315936 | 3A | 2011 | I.persulcatus | Russia, Perm' region | Kovalev et al. (2012) |
| Prm74592-2011 | JX315951 | 3A | 2011 | I.persulcatus | Russia, Perm' region | Kovalev et al. (2012) |
| Prm74880-2011 | JX315912 | 3A | 2011 | I.persulcatus | Russia, Perm' region | Kovalev et al. (2012) |
| Prm75610-2011 | JX315913 | 3A | 2011 | I.persulcatus | Russia, Perm' region | Kovalev et al. (2012) |
| Tmn252-2005 | GU444162 | 3A | 2005 | I.persulcatus | Russia, Tyumen region | Kovalev et al. (2009) |
| Tmn3001-2007 | JX315773 | 3A | 2007 | I.persulcatus | Russia, Tyumen region | Kovalev et al. (2012) |
| Tmn3002-2010 | JX315833 | 3A | 2010 | I.persulcatus | Russia, Tyumen region | Kovalev et al. (2012) |
| Tmn3011-2007 | JX315780 | 3A | 2007 | I.persulcatus | Russia, Tyumen region | Kovalev et al. (2012) |
| Tmn3018-2009 | JX315972 | 3A | 2009 | ? | Russia, Tyumen region | Kovalev et al. (2012) |
| Tmn3022-2010 | JX315835 | 3A | 2010 | I.persulcatus | Russia, Tyumen region | Kovalev et al. (2012) |
| Tmn3023-2010 | JX315836 | 3A | 2010 | I.persulcatus | Russia, Tyumen region | Kovalev et al. (2012) |
| Tmn3025-2008 | GU444274 | 3A | 2008 | I.persulcatus | Russia, Tyumen region | Kovalev et al. (2009) |
| Tmn3025-2010 | JX315838 | 3A | 2010 | I.persulcatus | Russia, Tyumen region | Kovalev et al. (2012) |
| Tmn3026-2010 | JX315839 | 3A | 2010 | I.persulcatus | Russia, Tyumen region | Kovalev et al. (2012) |
| Tmn3027-2010 | JX315840 | 3A | 2010 | I.persulcatus | Russia, Tyumen region | Kovalev et al. (2012) |
| Tmn3031-2008 | GU444275 | 3A | 2008 | I.persulcatus | Russia, Tyumen region | Kovalev et al. (2009) |
| Tmn3032-2010 | JX315841 | 3A | 2010 | I.persulcatus | Russia, Tyumen region | Kovalev et al. (2012) |
| Tmn3033-2010 | JX315842 | 3A | 2010 | I.persulcatus | Russia, Tyumen region | Kovalev et al. (2012) |
| Tmn3038-2008 | GU444276 | 3A | 2008 | I.persulcatus | Russia, Tyumen region | Kovalev et al. (2009) |
| Tmn3038-2010 | JX315844 | 3A | 2010 | I.persulcatus | Russia, Tyumen region | Kovalev et al. (2012) |
| Tmn3042-2009 | JX316000 | 3A | 2009 | I.persulcatus | Russia, Tyumen region | Kovalev et al. (2012) |
| Tmn3042-2010 | JX315848 | 3A | 2010 | I.persulcatus | Russia, Tyumen region | Kovalev et al. (2012) |
| Tmn3044-2010 | JX315849 | 3A | 2010 | I.persulcatus | Russia, Tyumen region | Kovalev et al. (2012) |
| Tmn3-2007 | GU444218 | 3A | 2007 | I.persulcatus | Russia, Tyumen region | Kovalev et al. (2009) |
| Tmn3282-2008 | GU444283 | 3A | 2008 | I.persulcatus | Russia, Tyumen region | Kovalev et al. (2009) |
| Tmn3313-2008 | GU444285 | 3A | 2008 | I.persulcatus | Russia, Tyumen region | Kovalev et al. (2009) |
| Tmn3501-2008 | JX315787 | 3A | 2008 | I.persulcatus | Russia, Tyumen region | Kovalev et al. (2012) |
| Tmn3504-2008 | JX315790 | 3A | 2008 | I.persulcatus | Russia, Tyumen region | Kovalev et al. (2012) |
| Tmn3505-2008 | JX315791 | 3A | 2008 | I.persulcatus | Russia, Tyumen region | Kovalev et al. (2012) |
| Tmn3506-2008 | JX315792 | 3A | 2008 | I.persulcatus | Russia, Tyumen region | Kovalev et al. (2012) |
| Tmn3510-2008 | JX315793 | 3A | 2008 | I.persulcatus | Russia, Tyumen region | Kovalev et al. (2012) |
| Tmn3511-2008 | JX315794 | 3A | 2008 | I.persulcatus | Russia, Tyumen region | Kovalev et al. (2012) |
| Tmn3512-2008 | JX315795 | 3A | 2008 | I.persulcatus | Russia, Tyumen region | Kovalev et al. (2012) |
| Tmn4003-2008 | JX315802 | 3A | 2008 | I.persulcatus | Russia, Tyumen region | Kovalev et al. (2012) |
| Tmn4004-2008 | JX315804 | 3A | 2008 | I.persulcatus | Russia, Tyumen region | Kovalev et al. (2012) |
| Tmn4006-2008 | JX315805 | 3A | 2008 | I.persulcatus | Russia, Tyumen region | Kovalev et al. (2012) |
| Tmn4008-2008 | JX315806 | 3A | 2008 | I.persulcatus | Russia, Tyumen region | Kovalev et al. (2012) |
| Tmn4009-2008 | JX315807 | 3A | 2008 | I.persulcatus | Russia, Tyumen region | Kovalev et al. (2012) |
| Tmn4010-2008 | JX315800 | 3A | 2008 | I.persulcatus | Russia, Tyumen region | Kovalev et al. (2012) |
| Tmn4025-2008 | JX315789 | 3A | 2008 | I.persulcatus | Russia, Tyumen region | Kovalev et al. (2012) |
| Tmn5-3-2007 | GU444242 | 3A | 2007 | I.persulcatus | Russia, Tyumen region | Kovalev et al. (2009) |
| Zausaev | AF527415 | 3A | 1985 | Human brain | Novosibirsk, Russia | Gritsun et al. (1993) |
| 1057 | EU443271 | 3A^2^ | 1995 | I. persulcatus | Academgorodok, Novosibirsk, West Siberia, Russia | Tkachev et al. (2008) |
| 1446 | EU443280 | 3A^2^ | 1998 | I. persulcatus | Academgorodok, Novosibirsk, West Siberia, Russia | Tkachev et al. (2008) |
| 1467 | AY753582 | 3A^2^ | ? | Tick | Novosibirsk region, West Siberia, Russia | Tkachev et al. (2004) |
| 1487 | JN936399 | 3A^2^ | 1999 | I. persulcatus | Novosibirsk region, Western Siberia, Russia | Tkachev et al. (2011) |
| 1658 | EF469763 | 3A^2^ | 2000 | I. persulcatus | Academgorodok, Novosibirsk, West Siberia, Russia | Tkachev et al. (2007) |
| 1746 | JN936410 | 3A^2^ | 2001 | I. persulcatus | Novosibirsk region, Western Siberia, Russia | Tkachev et al. (2011) |
| 1762 | JN936412 | 3A^2^ | 2001 | I. persulcatus | Novosibirsk region, Western Siberia, Russia | Tkachev et al. (2011) |
| 1763 | JN936413 | 3A^2^ | 2001 | I. persulcatus | Novosibirsk region, Western Siberia, Russia | Tkachev et al. (2011) |
| 1937 | EF467846 | 3A^2^ | 1985 | lab mouse brain | Academgorodok, Novosibirsk, West Siberia, Russia | Tkachev et al. (2007) |
| 2452 | GQ423570 | 3A^2^ | 2008 | I. persulcatus | South-Western Siberia, Novosibirsk, Russia | Morozova et al. (2009) |
| 2614 | GU060546 | 3A^2^ | 2009 | mouse brain infected with suspension from I.persulcatus | Russia, South-Western Siberia, Novosibirsk region | Morozova et al. (2009) |
| 348 | EF470574 | 3A^2^ | 1988 | I. persulcatus | Academgorodok, Novosibirsk, West Siberia, Russia | Tkachev et al. (2007) |
| 662 | EF470577 | 3A^2^ | 1992 | lab mouse brain | Academgorodok, Novosibirsk, West Siberia, Russia | Tkachev et al. (2008) |
| 755 | EF469738 | 3A^2^ | 1992 | I. persulcatus | Academgorodok, Novosibirsk, West Siberia, Russia | Tkachev et al. (2007) |
| 902 | EU443261 | 3A^2^ | 1994 | lab mouse brain | Academgorodok, Novosibirsk, West Siberia, Russia | Tkachev et al. (2008) |
| 92M | HM133640 | 3A^2^ | 2004 | ? | Mongolia | Khasnatinov, 2010 |
| Kemerovo-67-08 | GQ845432 | 3A^2^ | 2008 | I. persulcatus | Russia, West Siberia, Kemerovo region, Prokopyevsk, Chistugash | Karan et al. (2009) |
| Siberian-KY09 | HM641235 | 3A^2^ | 2009 | I. persulcatus | Kyrgyzstan | Briggs et al. (2010) |
| Z 22 | EF566816 | 3A^2^ | 2005 | I. persulcatus | Kemerovo, Siberia, Russia | Zoeller et al. (2007) |
| Ekb1055-2006 | JX315741 | 3B | 2006 | I.persulcatus | Russia: Tavatui, Nevyansk district, Sverdlovsk region | Kovalev et al. (2012) |
| Ekb1146-2006 | JX315742 | 3B | 2006 | I.persulcatus | Russia: Yekaterinburg, Sverdlovsk region | Kovalev et al. (2012) |
| Ekb125-2007 | GU444253 | 3B | 2007 | Human blood | Russia: Ural, Sverdlovsk region | Kovalev et al. (2009) |
| Ekb1270-2006 | JX315743 | 3B | 2006 | I.persulcatus | Russia: Yekaterinburg, Sverdlovsk region | Kovalev et al. (2012) |
| Ekb14-1975 | GU444139 | 3B | 1975 | I.persulcatus | Russia: Ural, Sverdlovsk region, Baikalovo district | Kovalev et al. (2009) |
| Ekb1729-2006 | JX315749 | 3B | 2006 | I.persulcatus | Russia: Dvurechensk, Sysert' district | Kovalev et al. (2012) |
| Ekb1735-2010 | JX315827 | 3B | 2010 | I.persulcatus | Russia: Yekaterinburg, Sverdlovsk region | Kovalev et al. (2012) |
| Ekb1924-2007 | JX315771 | 3B | 2007 | I.persulcatus | Russia: Beloyarsk district, Sverdlovsk region | Kovalev et al. (2012) |
| Ekb2036-2006 | JX315750 | 3B | 2006 | I.persulcatus | Russia: Yekaterinburg, Sverdlovsk region | Kovalev et al. (2012) |
| Ekb22-1975 | GU444141 | 3B | 1975 | I.persulcatus | Russia: Ural, Sverdlovsk region, Baikalovo district | Kovalev et al. (2009) |
| Ekb260503-2005 | JX315730 | 3B | 2005 | I.persulcatus | Russia: Sysert', Sverdlovsk region | Kovalev et al. (2012) |
| Ekb26-1975 | GU444142 | 3B | 1975 | I.persulcatus | Russia: Ural, Sverdlovsk region, Baikalovo district | Kovalev et al. (2009) |
| Ekb2886-2005 | JX315731 | 3B | 2005 | I.persulcatus | Russia: Yekaterinburg, Sverdlovsk region | Kovalev et al. (2012) |
| Ekb2971-2005 | JX315732 | 3B | 2005 | I.persulcatus | Russia: Yekaterinburg, Sverdlovsk region | Kovalev et al. (2012) |
| Ekb300505-2005 | JX315733 | 3B | 2005 | I.persulcatus | Russia: Yekaterinburg, Sverdlovsk region | Kovalev et al. (2012) |
| Ekb300517-2006 | JX315751 | 3B | 2006 | I.persulcatus | Russia: Verkh-Neivinsk, Sverdlovsk region | Kovalev et al. (2012) |
| Ekb326-2012 | KC886333 | 3B | 2012 | I.persulcatus | Russia: Sverdlovsk region, Verkhnyaya Pyshma | Kovalev et al. (2013) |
| Ekb33-1975 | GU444143 | 3B | 1975 | I.persulcatus | Russia: Ural, Sverdlovsk region, Baikalovo district | Kovalev et al. (2009) |
| Ekb4012-2005 | JX315734 | 3B | 2005 | I.persulcatus | Russia: Kolyutkino, Beloyarsk district, Sverdlovsk region | Kovalev et al. (2012) |
| Ekb4108-2005 | JX315735 | 3B | 2005 | I.persulcatus | Russia: Sysert', Sverdlovsk region | Kovalev et al. (2012) |
| Ekb42-1975 | GU444148 | 3B | 1975 | I.persulcatus | Russia: Ural, Sverdlovsk region, Baikalovo district | Kovalev et al. (2009) |
| Ekb440-2006 | JX315752 | 3B | 2006 | I.persulcatus | Russia: Vekhnyaya Sysert', Sverdlovsk region | Kovalev et al. (2012) |
| Ekb4486-2005 | JX315736 | 3B | 2005 | I.persulcatus | Russia: Sysert' district, Sverdlovsk region | Kovalev et al. (2012) |
| Ekb451-2005 | JX315737 | 3B | 2005 | I.persulcatus | Russia: Yekaterinburg, Sverdlovsk region | Kovalev et al. (2012) |
| Ekb5489-2005 | JX315738 | 3B | 2005 | I.persulcatus | Russia: Beryozovskyi, Sverdlovsk region | Kovalev et al. (2012) |
| Ekb554-2006 | JX315753 | 3B | 2006 | I.persulcatus | Russia: Sverdlovsk region + | Kovalev et al. (2012) |
| Ekb604-2006 | JX315754 | 3B | 2006 | I.persulcatus | Russia: Yekaterinburg, Sverdlovsk region | Kovalev et al. (2012) |
| Ekb60617-2005 | JX315739 | 3B | 2005 | I.persulcatus | Russia: Sysert', Sverdlovsk region | Kovalev et al. (2012) |
| Ekb621-2010 | JX315880 | 3B | 2010 | I.persulcatus | Russia: Nizhnyi Tagyl, Sverdlovsk region | Kovalev et al. (2012) |
| Ekb643-2006 | JX315756 | 3B | 2006 | I.persulcatus | Russia: Yekaterinburg, Sverdlovsk region | Kovalev et al. (2012) |
| Ekb7-1975 | GU444137 | 3B | 1975 | I.persulcatus | Russia: Ural, Sverdlovsk region, Baikalovo district | Kovalev et al. (2009) |
| Ekb847-2010 | JX315883 | 3B | 2010 | Human brain | Russia: Nizhnyi Tagyl, Sverdlovsk region | Kovalev et al. (2012) |
| Ekb905-2006 | JX315761 | 3B | 2006 | I.persulcatus | Russia: Yekaterinburg, Sverdlovsk region | Kovalev et al. (2012) |
| Ekb9-1975 | GU444138 | 3B | 1975 | I.persulcatus | Russia: Ural, Sverdlovsk region, Baikalovo district | Kovalev et al. (2009) |
| Omsk4026-2009 | JX315979 | 3B | 2009 | I.persulcatus | Russia, Omsk region | Kovalev et al. (2012) |
| Tmn4017-2008 | JX315781 | 3B | 2008 | I.persulcatus | Russia, Tyumen region | Kovalev et al. (2012) |
| 745 | EF470578 | 3C | 1992 | I. persulcatus | Academgorodok, Novosibirsk, West Siberia, Russia | Tkachev et al. (2007) |
| Ekb1032-2008 | GU444263 | 3C | 2008 | I.persulcatus | Russia: Ural, Sverdlovsk region, Verkhotur'e district | Kovalev et al. (2009) |
| Ekb1038-2008 | GU444264 | 3C | 2008 | I.persulcatus | Russia: Ural, Sverdlovsk region, Verkhotur'e district | Kovalev et al. (2009) |
| Ekb1039-2008 | GU444265 | 3C | 2008 | I.persulcatus | Russia: Ural, Sverdlovsk region, Verkhotur'e district | Kovalev et al. (2009) |
| Ekb1040-2008 | GU444266 | 3C | 2008 | I.persulcatus | Russia: Ural, Sverdlovsk region, Verkhotur'e district | Kovalev et al. (2009) |
| Ekb20-1975 | GU444140 | 3C | 1975 | I.persulcatus | Russia: Ural, Sverdlovsk region, Baikalovo district | Kovalev et al. (2009) |
| Ekb34-1975 | GU444144 | 3C | 1975 | I.persulcatus | Russia: Ural, Sverdlovsk region, Baikalovo district | Kovalev et al. (2009) |
| Ekb40-1975 | GU444146 | 3C | 1975 | I.persulcatus | Russia: Ural, Sverdlovsk region, Baikalovo district | Kovalev et al. (2009) |
| Ekb41-1975 | GU444147 | 3C | 1975 | I.persulcatus | Russia: Ural, Sverdlovsk region, Baikalovo district | Kovalev et al. (2009) |
| Ekb432-1977 | GU444158 | 3C | 1977 | I.persulcatus | Russia: Ural, Sverdlovsk region, Baikalovo district | Kovalev et al. (2009) |
| Ekb4591-2005 | GU444192 | 3C | 2005 | I.persulcatus | Russia: Ural, Sverdlovsk region, Yekaterinburg district | Kovalev et al. (2009) |
| Ekb532-1972 | GU444159 | 3C | 1972 | Human blood | Russia: Ural, Sverdlovsk region | Kovalev et al. (2009) |
| Krg1088-2009 | JX315977 | 3C | 2009 | I.persulcatus | Russia, Kurgan region | Kovalev et al. (2012) |
| Z 6 | EF566817 | 3C^2^ | 2005 | I. persulcatus | Kemerovo, Siberia, Russia | Zoeller et al. (2007) |
| Ekb4-2009 | JX315986 | 3C^3^ | 2009 | I.persulcatus | Russia: Pyshma district, Sverdlovsk region | Kovalev et al. (2012) |
| Tmn3519-2008 | JX315786 | 3C^3^ | 2008 | I.persulcatus | Russia, Tyumen region | Kovalev et al. (2012) |
| 1528 | EF469758 | 3D | 1999 | lab.mouse brain | Novosibirsk region, West Siberia, Russia | Tkachev et al. (2007) |
| Ekaterinburg-153-09 | GQ845418 | 3D | 2009 | ? | Ural, Sverdlovsk region, Sisert, Kashino, Russia | Karan et al. (2009) |
| Ekaterinburg-27-11-06 | FJ214123 | 3D | 2006 | I. persulcatus | Ekaterinburg, Ural, Russia | Karan et al. (2008) |
| Ekb131-2009 | JX315980 | 3D | 2009 | I.persulcatus | Russia: Kamyshlov, Sverdlovsk region | Kovalev et al. (2012) |
| Ekb140-2010 | JX315821 | 3D | 2010 | I.persulcatus | Russia: Kamyshlov district, Sverdlovsk region | Kovalev et al. (2012) |
| Ekb143-2010 | JX315822 | 3D | 2010 | I.persulcatus | Russia: Kamyshlov district, Sverdlovsk region | Kovalev et al. (2012) |
| Ekb146-2010 | JX315823 | 3D | 2010 | I.persulcatus | Russia: Kamyshlov district, Sverdlovsk region | Kovalev et al. (2012) |
| Ekb1656-7-1969 | GU444153 | 3D | 1969 | I.persulcatus | Russia: Ural, Sverdlovsk region, Talitsa district | Kovalev et al. (2009) |
| Ekb1956-2007 | GU444220 | 3D | 2007 | I.persulcatus | Russia: Ural, Sverdlovsk region, Beloyarsk district | Kovalev et al. (2009) |
| Ekb2172-2005 | GU444168 | 3D | 2005 | I.persulcatus | Russia: Ural, Sverdlovsk region, Sysert' district | Kovalev et al. (2009) |
| Ekb263-2009 | JX315985 | 3D | 2009 | I.persulcatus | Russia: Asbest, Sverdlovsk region | Kovalev et al. (2012) |
| Ekb335-2009 | JX315982 | 3D | 2009 | I.persulcatus | Russia: Kamyshlov, Sverdlovsk region | Kovalev et al. (2012) |
| Ekb373-2010 | JX315854 | 3D | 2010 | I.persulcatus | Russia: Kamyshlov district, Sverdlovsk region | Kovalev et al. (2012) |
| Ekb40-2009 | JX315983 | 3D | 2009 | I.persulcatus | Russia: Kamyshlov, Sverdlovsk region | Kovalev et al. (2012) |
| Ekb5-1-2006 | GU444194 | 3D | 2006 | I.persulcatus | Russia: Ural, Sverdlovsk region, Sysert' district | Kovalev et al. (2009) |
| Ekb5263-2011 | JX315890 | 3D | 2011 | I.persulcatus | Russia: Sverdlovsk region | Kovalev et al. (2012) |
| Ekb5444-2005 | GU444183 | 3D | 2005 | I.persulcatus | Russia: Ural, Sverdlovsk region, Sysert' district | Kovalev et al. (2009) |
| Ekb788-2006 | GU444206 | 3D | 2006 | I.persulcatus | Russia: Ural, Sverdlovsk region, Beloyarsk district | Kovalev et al. (2009) |
| Ekb9-2-2006 | GU444195 | 3D | 2006 | I.persulcatus | Russia: Ural, Sverdlovsk region, Kamyshlov district | Kovalev et al. (2009) |
| Ekb9300115-2012 | KC886349 | 3D | 2012 | I.persulcatus | Russia: Sverdlovsk region, Yekaterinburg | Kovalev et al. (2013) |
| Est54 | DQ393773 | 3D | 2000 | I. persulcatus | Estonia | Golovljova et al. (2004) |
| Karelia-94 | HM051173 | 3D | 2006 | ? | Karelia, Russia | Jaaskelainen et al. (2010) |
| Kurgan-264-07 | FJ214130 | 3D | 2007 | I. persulcatus | Kurgan, Ural, Russia | Karan et al. (2008) |
| Kurgan-269-07 | FJ214129 | 3D | 2007 | I. persulcatus | Kurgan, Ural, Russia | Karan et al. (2008) |
| Kurgan-272-07 | FJ214128 | 3D | 2007 | I. persulcatus | Kurgan, Ural, Russia | Karan et al. (2008) |
| Kurgan-273-07 | FJ214131 | 3D | 2007 | I. persulcatus | Kurgan, Ural, Russia | Karan et al. (2008) |
| Latvia-1-96 | AJ415565 | 3D | 2001 | Human blood | Latvia | Lundkvist et al. (2001) |
| Latvia-1-96 | GU183382 | 3D | 1996 | Human TBE case | Latvia | Uzcategui et al. (2012) |
| Omsk4006-2010 | JX315860 | 3D | 2010 | Dermacentor sp. | Russia, Omsk region | Kovalev et al. (2012) |
| Omsk4016-2010 | JX315862 | 3D | 2010 | I.persulcatus | Russia, Omsk region | Kovalev et al. (2012) |
| Omsk4019-2010 | JX315864 | 3D | 2010 | I.persulcatus | Russia, Omsk region | Kovalev et al. (2012) |
| Omsk4037-2010 | JX315866 | 3D | 2010 | I.persulcatus | Russia, Omsk region | Kovalev et al. (2012) |
| Omsk4047-2008 | JX315783 | 3D | 2008 | I.persulcatus | Russia, Omsk region | Kovalev et al. (2012) |
| Omsk4069-2010 | JX315870 | 3D | 2010 | I.persulcatus | Russia, Omsk region | Kovalev et al. (2012) |
| Omsk4071-2008 | JX315785 | 3D | 2008 | D.reticulatus | Russia, Omsk region | Kovalev et al. (2012) |
| Omsk4078-2010 | JX315874 | 3D | 2010 | I.persulcatus | Russia, Omsk region | Kovalev et al. (2012) |
| Prm73904-2011 | JX315895 | 3D | 2011 | I.persulcatus | Russia, Perm' region | Kovalev et al. (2012) |
| TBEV-Estonia54 | GU183384 | 3D | 2000 | I. persulcatus | Estonia | Uzcategui et al. (2012) |
| Tmn3024-2010 | JX315837 | 3D | 2010 | I.persulcatus | Russia, Tyumen region | Kovalev et al. (2012) |
| Tmn3258-2008 | GU444286 | 3D | 2008 | I.persulcatus | Russia, Tyumen region | Kovalev et al. (2009) |
| Tmn9-2-2007 | GU444244 | 3D | 2007 | I.persulcatus | Russia, Tyumen region | Kovalev et al. (2009) |
| Vologda-166-08 | GQ845435 | 3D | 2008 | I. persulcatus | Russia, European part, North-West, Vologda | Karan et al. (2009) |
| Vologda-173-08 | GQ845436 | 3D | 2008 | I. persulcatus | Russia, European part, North-West, Vologda | Karan et al. (2009) |
| Vologda-3-75 | FJ214143 | 3D | 1975 | I. persulcatus | Vologda, northwestern region, Russia | Karan et al. (2008) |
| Vologda-509-75 | FJ214142 | 3D | 1975 | I. persulcatus | Vologda, northwestern region, Russia | Karan et al. (2008) |
| Vologda-911-74 | FJ214138 | 3D | 1974 | Human blood | Vologda, northwestern region, Russia | Karan et al. (2008) |
| Vologda-Krash-08 | GQ845438 | 3D | 2008 | brain tissue of deceased patient | Russia, European part, North-West, Vologda | Karan et al. (2009) |
| Yaroslavl-115-01 | FJ214145 | 3D | 2001 | I. persulcatus | Yaroslavl, Central region, Russia | Karan et al. (2008) |
| Yaroslavl-140-98 | FJ214146 | 3D | 1998 | I. persulcatus | Yaroslavl, Central region, Russia | Karan et al. (2008) |
| Yaroslavl-Aver-08 | GQ845440 | 3D | 2008 | brain tissue of deceased patient | Russia, European part, Yaroslavl | Karan et al. (2009) |
| Yaroslavl-Bel-08 | GQ845439 | 3D | 2008 | brain tissue of deceased patient | Russia, European part, Yaroslavl | Karan et al. (2009) |
| Ekb1148-2-2006 | GU444210 | 3E | 2006 | I.persulcatus | Russia: Ural, Sverdlovsk region, Sysert' district | Kovalev et al. (2009) |
| Ekb361-2008 | GU444279 | 3E | 2008 | I.persulcatus | Russia: Ural, Sverdlovsk region, Sysert' district | Kovalev et al. (2009) |
| Ekb362-2008 | GU444280 | 3E | 2008 | I.persulcatus | Russia: Ural, Sverdlovsk region, Sysert' district | Kovalev et al. (2009) |
| Ekb365-2008 | GU444284 | 3E | 2008 | I.persulcatus | Russia: Ural, Sverdlovsk region, Tavda district | Kovalev et al. (2009) |
| EkbKK-1968 | JX315722 | 3E | 1968 | Cow blood | Russia: Nizhnyi Tagil, Sverdlovsk region | Kovalev et al. (2012) |
| 127-10 | KC417475 | 3F | 2010 | I. persulcatus | Russia: Irkutsk region | Adelshin et al. (2012) |
| 542-10 | KC417476 | 3F | 2010 | I. persulcatus | Russia: Irkutsk region | Adelshin et al. (2012) |
| Bsh2001-2009 | JX315958 | 3F | 2009 | I.persulcatus | Russia, Bashkiria region | Kovalev et al. (2012) |
| Ekaterinburg-5-09 | GQ845424 | 3F | 2009 | I. persulcatus | Ural, Sverdlovsk region, Ekaterinburg, Sisert | Karan et al. (2009) |
| Ekaterinburg-992-09 | GQ845419 | 3F | 2009 | I. persulcatus | Ural, Sverdlovsk region, Ekaterinburg, Koltsovo | Karan et al. (2009) |
| Ekb102-2012 | KC886324 | 3F | 2012 | I.persulcatus | Russia: Sverdlovsk region, Polevskoi district | Kovalev et al. (2013) |
| Ekb11a-2007 | JX315762 | 3F | 2007 | I.persulcatus | Russia: Pyshma district, Sverdlovsk region | Kovalev et al. (2012) |
| Ekb122-2010 | JX315819 | 3F | 2010 | I.persulcatus | Russia: Asbest district, Sverdlovsk region | Kovalev et al. (2012) |
| Ekb13578-2011 | JX315897 | 3F | 2011 | I.persulcatus | Russia: Beryozovsk, Sverdlovsk region | Kovalev et al. (2012) |
| Ekb14474-2011 | JX315916 | 3F | 2011 | I.persulcatus | Russia: Pervouralsk district, Sverdlovsk region | Kovalev et al. (2012) |
| Ekb16-2007 | GU444222 | 3F | 2007 | I.persulcatus | Russia: Ural, Sverdlovsk region, Polevskoi district | Kovalev et al. (2009) |
| Ekb1906-2007 | GU444219 | 3F | 2007 | I.persulcatus | Russia: Ural, Sverdlovsk region, Beryozovsk district | Kovalev et al. (2009) |
| Ekb19-2007 | GU444230 | 3F | 2007 | I.persulcatus | Russia: Ural, Sverdlovsk region, Polevskoi district | Kovalev et al. (2009) |
| Ekb23-2007 | JX315763 | 3F | 2007 | I.persulcatus | Russia: Polevskoi district, Sverdlovsk region | Kovalev et al. (2012) |
| Ekb25125-2009 | JX315960 | 3F | 2009 | Human blood | Russia: Sverdlovsk region + | Kovalev et al. (2012) |
| Ekb254-2005 | GU444163 | 3F | 2005 | I.persulcatus | Russia: Ural, Sverdlovsk region | Kovalev et al. (2009) |
| Ekb26-2007 | GU444231 | 3F | 2007 | I.persulcatus | Russia: Ural, Sverdlovsk region, Polevskoi district | Kovalev et al. (2009) |
| Ekb275-2005 | GU444190 | 3F | 2005 | I.persulcatus | Russia: Ural, Sverdlovsk region, Zarech'nyi | Kovalev et al. (2009) |
| Ekb32-2008 | GU444254 | 3F | 2008 | I.persulcatus | Russia: Ural, Sverdlovsk region, Polevskoi district | Kovalev et al. (2009) |
| Ekb34-2008 | GU444255 | 3F | 2008 | I.persulcatus | Russia: Ural, Sverdlovsk region, Polevskoi district | Kovalev et al. (2009) |
| Ekb35-2008 | GU444256 | 3F | 2008 | I.persulcatus | Russia: Ural, Sverdlovsk region, Polevskoi district | Kovalev et al. (2009) |
| Ekb405-2011 | JX315956 | 3F | 2011 | I.persulcatus | Russia: Sysert' district, Sverdlovsk region | Kovalev et al. (2012) |
| Ekb432-2011 | JX315923 | 3F | 2011 | I.persulcatus | Russia: Asbest district, Sverdlovsk region | Kovalev et al. (2012) |
| Ekb44-2007 | GU444235 | 3F | 2007 | I.persulcatus | Russia: Ural, Sverdlovsk region, Polevskoi district | Kovalev et al. (2009) |
| Ekb51-2007 | JX315764 | 3F | 2007 | I.persulcatus | Russia: Polevskoi district, Sverdlovsk region | Kovalev et al. (2012) |
| Ekb5275-2005 | GU444181 | 3F | 2005 | I.persulcatus | Russia: Ural, Sverdlovsk region, Sysert' district | Kovalev et al. (2009) |
| Ekb54-2007 | GU444236 | 3F | 2007 | I.persulcatus | Russia: Ural, Sverdlovsk region, Polevskoi district | Kovalev et al. (2009) |
| Ekb55-2007 | GU444237 | 3F | 2007 | I.persulcatus | Russia: Ural, Sverdlovsk region, Polevskoi district | Kovalev et al. (2009) |
| Ekb67-2011 | JX315926 | 3F | 2011 | I.persulcatus | Russia: Asbest district, Sverdlovsk region | Kovalev et al. (2012) |
| Ekb68-2011 | JX315924 | 3F | 2011 | I.persulcatus | Russia: Asbest district, Sverdlovsk region | Kovalev et al. (2012) |
| Ekb77-2007 | GU444238 | 3F | 2007 | I.persulcatus | Russia: Ural, Sverdlovsk region, Polevskoi district | Kovalev et al. (2009) |
| Ekb81-2007 | JX315765 | 3F | 2007 | I.persulcatus | Russia: Polevskoi district, Sverdlovsk region | Kovalev et al. (2012) |
| Ekb83-2011 | JX315925 | 3F | 2011 | I.persulcatus | Russia: Kamensk, Sverdlovsk region | Kovalev et al. (2012) |
| Ekb91-I-2007 | GU444225 | 3F | 2007 | Human blood | Russia: Ural, Sverdlovsk region, Kamyshlov district | Kovalev et al. (2009) |
| Ekb92-2007 | GU444240 | 3F | 2007 | I.persulcatus | Russia: Ural, Sverdlovsk region, Kamensk-Ural'sk district | Kovalev et al. (2009) |
| Lesopark | GU121966 | 3F | 1986 | ? | Russia, West Siberia | Kozlovskaya et al. (2009) |
| Omsk4014-2009 | JX315959 | 3F | 2009 | D.reticulatus | Russia, Omsk region | Kovalev et al. (2012) |
| Prm73856-2011 | JX315957 | 3F | 2011 | I.persulcatus | Russia, Perm' region | Kovalev et al. (2012) |
| Prm7522-2011 | JX315898 | 3F | 2011 | I.persulcatus | Russia, Perm' region | Kovalev et al. (2012) |
| Prm7523-2011 | JX315899 | 3F | 2011 | I.persulcatus | Russia, Perm' region | Kovalev et al. (2012) |
| Tmn8-2007 | GU444243 | 3F | 2007 | I.persulcatus | Russia, Tyumen region | Kovalev et al. (2009) |
| Kemerovo-49-08 | GQ845430 | 3F^2^ | 2008 | I. persulcatus | Russia, West Siberia, Kemerovo region, Prokopyevsk, Chistugash | Karan et al. (2009) |
| Kokkola-102 | DQ451295 | 3G | 2006 | I. persulcatus | Finland | Jaaskelainen et al. (2005) |
| Kokkola-118 | DQ451296 | 3G | 2006 | I. persulcatus | Finland | Jaaskelainen et al. (2005) |
| Kokkola-39 | DQ451290 | 3G | 2006 | I. persulcatus | Finland | Jaaskelainen et al. (2005) |
| Kokkola-79 | DQ451291 | 3G | 2006 | I. persulcatus | Finland | Jaaskelainen et al. (2005) |
| Kokkola-8 | DQ451286 | 3G | 2006 | I. persulcatus | Finland | Jaaskelainen et al. (2005) |
| Kokkola-81 | DQ451292 | 3G | 2006 | I. persulcatus | Finland | Jaaskelainen et al. (2005) |
| Kokkola-86 | DQ451294 | 3G | 2006 | I. persulcatus | Finland | Jaaskelainen et al. (2005) |
| Kokkola-9 | DQ451287 | 3G | 2006 | I. persulcatus | Finland | Jaaskelainen et al. (2005) |
| Vologda-208-08 | GQ845437 | 3G | 2008 | I. persulcatus | Russia, European part, North-West, Vologda | Karan et al. (2009) |
| 1128 | EU443274 | 3H | 1995 | lab mouse brain | Academgorodok, Novosibirsk, West Siberia, Russia | Tkachev et al. (2008) |
| 942 | EF469744 | 3H | 1994 | lab mouse brain | Academgorodok, Novosibirsk, West Siberia, Russia | Tkachev et al. (2007) |
| Aina | AF091006 | 3H | 1963 | Human blood | Irkutsk, Russia | Ecker et al. (1999) |
| Aina | JN003206 | 3H | 1963 | Homo sapiens | Russia | Kulakova et al. (2011) |
| Cht-22 | JN003208 | 3H | 2002 | Homo sapiens | Russia | Romanova et al. (2011) |
| Cht-653 | JN003207 | 3H | 1995 | Homo sapiens | Russia | Romanova et al. (2011) |
| Irkutsk-112-79 | FJ214156 | 3H | 1979 | I. persulcatus | Irkutsk, East Siberia, Russia | Karan et al. (2008) |
| Irkutsk-12 | JN003209 | 3H | 2010 | Homo sapiens | Russia | Kulakova et al. (2011) |
| Vasilchenko | L40361 | 3H | ? |  |  | Gritsun et al. (2000) |
| Vasilchenko | M97369 | 3H | 1969 | Human blood | Novosibirsk, Russia | Gritsun et al. (1993) |
| Z 12 | EF566814 | 3H | 2005 | I. persulcatus | Kemerovo, Siberia, Russia | Zoeller et al. (2007) |
| Z 14 | EF566815 | 3H | 2005 | I. persulcatus | Kemerovo, Siberia, Russia | Zoeller et al. (2007) |
| Z 7 | EF566818 | 3H | 2005 | I. persulcatus | Kemerovo, Siberia, Russia | Zoeller et al. (2007) |
| Zabaikalye 11-99 | KC414090 | 3H | 1999 | Human brain | Russia: Zabaikalye | Sidorova et al. (2012) |
| Ekb11869-2011 | JX315942 | 3I | 2011 | I.persulcatus | Russia: Yekaterinburg, Sverdlovsk region | Kovalev et al. (2012) |
| Ekb314-2009 | JX315992 | 3I | 2009 | I.persulcatus | Russia: Beryozovskyi, Sverdlovsk region | Kovalev et al. (2012) |
| Ekb315-2009 | JX315993 | 3I | 2009 | I.persulcatus | Russia: Beryozovskyi, Sverdlovsk region | Kovalev et al. (2012) |
| Ekb316-2009 | JX315994 | 3I | 2009 | I.persulcatus | Russia: Beryozovskyi, Sverdlovsk region | Kovalev et al. (2012) |
| Ekb394-2010 | JX315855 | 3I | 2010 | I.persulcatus | Russia: Beryozovsk district, Sverdlovsk region | Kovalev et al. (2012) |
| Ekb396-2010 | JX315856 | 3I | 2010 | I.persulcatus | Russia: Beryozovsk district, Sverdlovsk region | Kovalev et al. (2012) |
| Ekb398-2010 | JX315857 | 3I | 2010 | I.persulcatus | Russia: Beryozovsk district, Sverdlovsk region | Kovalev et al. (2012) |
| Ekb400-2010 | JX315858 | 3I | 2010 | I.persulcatus | Russia: Beryozovsk district, Sverdlovsk region | Kovalev et al. (2012) |
| Ekb462-2011 | JX315944 | 3I | 2011 | I.persulcatus | Russia: Beryozovsk district, Sverdlovsk region | Kovalev et al. (2012) |
| Ekb491-2011 | JX315943 | 3I | 2011 | I.persulcatus | Russia: Sysert' district, Sverdlovsk region | Kovalev et al. (2012) |
| Ekb669-2006 | GU444204 | 3I | 2006 | I.persulcatus | Russia: Ural, Sverdlovsk region, Beryozovsk district | Kovalev et al. (2009) |
| IR99-1m1 | AB049348 | 3J | 1999 | I .persulcatus | Irkutsk, Russia | Hayasaka et al. (2001) |
| Kemerovo-K34-67 | FJ214134 | 3J | 1967 | Human blood | Kemerovo, West Siberia, Russia | Karan et al. (2008) |
| Z 10 | EF566813 | 3J | 2005 | I. persulcatus | Kemerovo, Siberia, Russia | Zoeller et al. (2007) |
| 1130 | EU443275 | 3K | 1995 | I. persulcatus | Academgorodok, Novosibirsk, West Siberia, Russia | Tkachev et al. (2008) |
| 1145 | JN936391 | 3K | 1995 | I. persulcatus | Novosibirsk region, Western Siberia, Russia | Tkachev et al. (2011) |
| 980 | JN936386 | 3K | 1994 | I. persulcatus | Novosibirsk region, Western Siberia, Russia | Tkachev et al. (2011) |
| Ekaterinburg-14-5-06 | FJ214125 | 3K | 2006 | I. persulcatus | Ekaterinburg, Ural, Russia | Karan et al. (2008) |
| Ekb914-2011 | JX315909 | 3K | 2011 | Human brain | Russia: Yekaterinburg, Sverdlovsk region | Kovalev et al. (2012) |
| Ekb93-2011 | JX315902 | 3K | 2011 | I.persulcatus | Russia: Kamensk, Sverdlovsk region | Kovalev et al. (2012) |
| 1668 | JN936403 | 3L^2^ | ? | I. persulcatus | Novosibirsk region, Western Siberia, Russia | Tkachev et al. (2011) |
| 1675 | EF469764 | 3L^2^ | 2001 | I. persulcatus | Russia: West Siberia, Novosibirsk, Academgorodok | Tkachev et al. (2007) |
| 1676 | JN936404 | 3L^2^ | 2001 | I. persulcatus | Novosibirsk region, Western Siberia, Russia | Tkachev et al. (2011) |
| 1690 | JN936405 | 3L^2^ | 2001 | I. persulcatus | Novosibirsk region, Western Siberia, Russia | Tkachev et al. (2011) |
| 1693 | JN936406 | 3L^2^ | 2001 | I. persulcatus | Novosibirsk region, Western Siberia, Russia | Tkachev et al. (2011) |
| 1699 | EU443268 | 3L^2^ | 2001 | I. persulcatus | Academgorodok, Novosibirsk, West Siberia, Russia | Tkachev et al. (2008) |
| 1700 | EU443284 | 3L^2^ | 2001 | I. persulcatus | Academgorodok, Novosibirsk, West Siberia, Russia | Tkachev et al. (2008) |
| Ekb1632-2006 | GU444211 | 3L | 2006 | I.persulcatus | Russia: Ural, Sverdlovsk region, Yekaterinburg district | Kovalev et al. (2009) |
| 24 | GU143822 | 3M | 2009 | I. persulcatus | Russia: Ural, Chelyabinsk region | Morozova et al. (1999) |
| Ekb52-2012 | KC886338 | 3M | 2012 | I.persulcatus | Russia: Sverdlovsk region, Yekaterinburg | Kovalev et al. (2013) |
| Tmn3163-2009 | JX315975 | 3M | 2009 | I.persulcatus | Russia, Tyumen region | Kovalev et al. (2012) |
| Tmn3249-2008 | GU444277 | 3M | 2008 | I.persulcatus | Russia, Tyumen region | Kovalev et al. (2009) |
| 766 | EF469739 | 3N | 1992 | I. persulcatus | Academgorodok, Novosibirsk, West Siberia, Russia | Tkachev et al. (2007) |
| Ekb4954-1984 | GU444129 | 3N | 1984 | Human brain | Russia: Ural, Sverdlovsk region, Beloyarsk -district | Kovalev et al. (2009) |
| IR99-lm4 | AB049349 | 3N | 1999 | I. persulcatus | Irkutsk, Russia | Hayasaka et al. (2001) |
| Ekb150-2010 | JX315824 | 3O | 2010 | I.persulcatus | Russia: Kamyshlov district, Sverdlovsk region | Kovalev et al. (2012) |
| Ekb334-2009 | JX315984 | 3O | 2009 | I.persulcatus | Russia: Kamyshlov, Sverdlovsk region | Kovalev et al. (2012) |
| Ekb90-I-2007 | GU444224 | 3O | 2007 | Human blood | Russia: Ural, Sverdlovsk region, Kamyshlov district | Kovalev et al. (2009) |
| Ekb13457-2011 | JX315891 | 3P | 2011 | I.persulcatus | Russia: Yekaterinburg, Sverdlovsk region | Kovalev et al. (2012) |
| Ekb14142-2011 | JX315892 | 3P | 2011 | I.persulcatus | Russia: Sverdlovsk region | Kovalev et al. (2012) |
| Ekb79-2011 | JX315893 | 3P | 2011 | I.persulcatus | Russia: Kamensk, Sverdlovsk region | Kovalev et al. (2012) |
| Karelia-108 | HM051174 | 3P | 2006 | ? | Karelia, Russia | Jaaskelainen et al. (2010) |
| SPb10-2008 | JX315782 | 3P | 2008 | I.persulcatus | Russia, Leningrad region | Kovalev et al. (2012) |
| Vologda-14-06 | FJ214140 | 3P | 2006 | I. persulcatus | Vologda, northwestern region, Russia | Karan et al. (2008) |
| Vologda-15-06 | FJ214141 | 3P | 2006 | I. persulcatus | Vologda, northwestern region, Russia | Karan et al. (2008) |
| Vologda-4-06 | FJ214139 | 3P | 2006 | I. persulcatus | Vologda, northwestern region, Russia | Karan et al. (2008) |
| Ekb1182-2007 | JX315770 | 3Q | 2007 | I.persulcatus | Russia: Nizhnyi Tagil, Sverdlovsk region | Kovalev et al. (2012) |
| Ekb1605-2006 | JX315744 | 3Q | 2006 | I.persulcatus | Russia: Serov district, Sverdlovsk region | Kovalev et al. (2012) |
| Ekb725-2006 | JX315747 | 3Q | 2006 | I.persulcatus | Russia: Yekaterinburg, Sverdlovsk region | Kovalev et al. (2012) |
| Ekb752-2005 | JX315727 | 3Q | 2005 | I.persulcatus | Russia: Kashino, Sysert' district, Sverdlovsk region | Kovalev et al. (2012) |
| Ekb753-2006 | JX315748 | 3Q | 2006 | I.persulcatus | Russia: Asbest, Sverdlovsk region | Kovalev et al. (2012) |
| 617-90 | EU878283 | 3U | 1990 | I. persulcatus | Buryat Republic, Eastern Siberia, Russia | Tkachev et al. (2008)) |
| 711-84 | EU878281 | 3U | 1984 | Clethrionomys rufocanus | Buryat Republic, Eastern Siberia, Russia | Tkachev et al. (2008) |
| 740-84 | EU878282 | 3U | 1984 | Clethrionomys rufocanus | Buryat Republic, Eastern Siberia, Russia | Tkachev et al. (2008) |
| 886-84 | EF469662 | 3U | 1984 | C. rufocanus (wild rodents) | Irkutsk region, Russia | Karan et al. (1999) |
| 1017 | JN936387 | 3Unique | 1994 | I. persulcatus | Novosibirsk region, Western Siberia, Russia | Tkachev et al. (2011) |
| 1047 | JN936388 | 3Unique | 1995 | I. persulcatus | Novosibirsk region, Western Siberia, Russia | Tkachev et al. (2011) |
| 1052 | JN936389 | 3Unique | 1995 | I. persulcatus | Novosibirsk region, Western Siberia, Russia | Tkachev et al. (2011) |
| 1143 | JN936390 | 3Unique | 1995 | I. persulcatus | Novosibirsk region, Western Siberia, Russia | Tkachev et al. (2011) |
| 116-10 | KC417474 | 3Unique | 2010 | I. persulcatus | Russia: Irkutsk region | Adelshin et al. (2012) |
| 1212 | JN936393 | 3Unique | 1996 | I. persulcatus | Novosibirsk region, Western Siberia, Russia | Tkachev et al. (2011) |
| 1248 | JN936394 | 3Unique | 1996 | I. persulcatus | Novosibirsk region, Western Siberia, Russia | Tkachev et al. (2011) |
| 1252 | DQ394879 | 3Unique | 1982 | I. persulcatus | Moshkovo outskirts, Novosibirsk region, Western Siberia, Russia | Tkachev et al. (2006) |
| 1259 | EU443277 | 3Unique | 1996 | mouse brain | Novosibirsk region, Western Siberia, Russia | Tkachev et al. (2011) |
| 1284 | EU443278 | 3Unique | 1996 | I. persulcatus | Academgorodok, Novosibirsk, West Siberia, Russia | Tkachev et al. (2008) |
| 1346 | EU443279 | 3Unique | 1997 | I. persulcatus | Academgorodok, Novosibirsk, West Siberia, Russia | Tkachev et al. (2008) |
| 1395 | JN936396 | 3Unique | 1997 | I. persulcatus | Novosibirsk region, Western Siberia, Russia | Tkachev et al. (2011) |
| 1430 | JN936397 | 3Unique | 1998 | I. persulcatus | Novosibirsk region, Western Siberia, Russia | Tkachev et al. (2011) |
| 1440 | JN936398 | 3Unique | 1998 | I. persulcatus | Novosibirsk region, Western Siberia, Russia | Tkachev et al. (2011) |
| 1441 | EU443269 | 3Unique | 1984 | I. persulcatus | Academgorodok, Novosibirsk, West Siberia, Russia | Tkachev et al. (2008) |
| 1527 | JN936400 | 3Unique | 1999 | I. persulcatus | Novosibirsk region, Western Siberia, Russia | Tkachev et al. (2011) |
| 1551 | JN936401 | 3Unique | 1999 | I. persulcatus | Novosibirsk region, Western Siberia, Russia | Tkachev et al. (2011) |
| 1579 | JN936402 | 3Unique | 1999 | I. persulcatus | Novosibirsk region, Western Siberia, Russia | Tkachev et al. (2011) |
| 16 | GU143821 | 3Unique | 2009 | I. persulcatus | Russia, Ural, Chelyabinsk region | Morozova et al. (2009) |
| 2033 | JN936379 | 3Unique | 1985 | I. persulcatus | Novosibirsk region, Western Siberia, Russia | Tkachev et al. (2011) |
| 2045 | JN936380 | 3Unique | 1985 | I. persulcatus | Novosibirsk region, Western Siberia, Russia | Tkachev et al. (2011) |
| 212 | JN936381 | 3Unique | 1988 | I. persulcatus | Novosibirsk region, Western Siberia, Russia | Tkachev et al. (2011) |
| 228 | DQ385498 | 3Unique | 1981 | I. persulcatus | Western Siberia, Russia | Tkachev et al. (2006) |
| 228 | EF467840 | 3Unique | 1981 | I. persulcatus | Academgorodok, Novosibirsk, West Siberia, Russia | Tkachev et al. (2008) |
| 240 | JN936383 | 3Unique | 1988 | I. persulcatus | Novosibirsk region, Western Siberia, Russia | Tkachev et al. (2011) |
| 2401 | GQ423568 | 3Unique | 2007 | I. persulcatus | South-Western Siberia, Novosibirsk, Russia | Morozova et al. (2009) |
| 323 | EU443263 | 3Unique | 1988 | I. persulcatus | Academgorodok, Novosibirsk, West Siberia, Russia | Tkachev et al. (2008) |
| 350 | JN936384 | 3Unique | 1988 | I. persulcatus | Novosibirsk region, Western Siberia, Russia | Tkachev et al. (2011) |
| 499 | DQ394877 | 3Unique | 1982 | I. persulcatus | Western Siberia, Russia | Tkachev et al. (2006) |
| 668 | EU443260 | 3Unique | 1992 | I. persulcatus | Academgorodok, Novosibirsk, West Siberia, Russia | Tkachev et al. (2008) |
| 691 | DQ394878 | 3Unique | 1982 | I. persulcatus | Western Siberia, Russia | Tkachev et al. (2006) |
| 691-1 | EU443264 | 3Unique | 1982 | I. persulcatus | Academgorodok, Novosibirsk, West Siberia, Russia | Tkachev et al. (2008) |
| 919 | EF469740 | 3Unique | 1994 | I. persulcatus | Academgorodok, Novosibirsk, West Siberia, Russia | Tkachev et al. (2007) |
| Altai731-2011 | JX315887 | 3Unique | 2011 | I.persulcatus | Russia, Altai region | Kovalev et al. (2012) |
| Bashkiria-351-09 | GQ845423 | 3Unique | 2009 | I. persulcatus | Russia, Bashkiria | Karan et al. (2009) |
| Chelyabinsk-357-09 | GQ845422 | 3Unique | 2009 | I. persulcatus | Ural, Chelyabinsk region | Karan et al. (2009) |
| EK328 | DQ486861 | 3Unique | 1972 | I. persulcatus | Estonia | Romanova et al. (2007) |
| Ekb10-2007 | GU444248 | 3Unique | 2007 | I.persulcatus | Russia: Ural, Sverdlovsk region, Nyzhnyi Tagil district | Kovalev et al. (2009) |
| Ekb1068-2008 | GU444267 | 3Unique | 2008 | I.persulcatus | Russia: Ural, Sverdlovsk region, Baikalovo district | Kovalev et al. (2009) |
| Ekb1070-2008 | GU444268 | 3Unique | 2008 | I.persulcatus | Russia: Ural, Sverdlovsk region, Baikalovo district | Kovalev et al. (2009) |
| Ekb11283-2011 | JX315889 | 3Unique | 2011 | I.persulcatus | Russia: Polevskoi, Sverdlovsk region | Kovalev et al. (2012) |
| Ekb1148-2006 | GU444209 | 3Unique | 2006 | I.persulcatus | Russia: Ural, Sverdlovsk region, Sysert' district | Kovalev et al. (2009) |
| Ekb1193-2010 | JX315815 | 3Unique | 2010 | I.persulcatus | Russia: Beloyarsk district, Sverdlovsk region | Kovalev et al. (2012) |
| Ekb12209-2011 | JX315888 | 3Unique | 2011 | I.persulcatus | Russia: Beloyarsk district, Sverdlovsk region | Kovalev et al. (2012) |
| Ekb12265-2011 | JX315896 | 3Unique | 2011 | I.persulcatus | Russia: Beloyarsk, Sverdlovsk region | Kovalev et al. (2012) |
| Ekb12-9-2006 | GU444196 | 3Unique | 2006 | I.persulcatus | Russia: Ural, Sverdlovsk region, Sysert' district | Kovalev et al. (2009) |
| Ekb13581-2011 | JX315900 | 3Unique | 2011 | I.persulcatus | Russia: Beloyarsk district, Sverdlovsk region | Kovalev et al. (2012) |
| Ekb143-I-2007 | JX315777 | 3Unique | 2007 | Human blood | Russia: Sverdlovsk region | Kovalev et al. (2012) |
| Ekb170604-2005 | GU444185 | 3Unique | 2005 | I.persulcatus | Russia: Ural, Sverdlovsk region, Sysert' district | Kovalev et al. (2009) |
| Ekb1715-2006 | GU444212 | 3Unique | 2006 | I.persulcatus | Russia: Ural, Sverdlovsk region, Yekaterinburg district | Kovalev et al. (2009) |
| Ekb1715-2-2006 | GU444213 | 3Unique | 2006 | I.persulcatus | Russia: Ural, Sverdlovsk region, Yekaterinburg district | Kovalev et al. (2009) |
| Ekb17-2007 | GU444223 | 3Unique | 2007 | I.persulcatus | Russia: Ural, Sverdlovsk region, Polevskoi district district | Kovalev et al. (2009) |
| Ekb174-2012 | KC886328 | 3Unique | 2012 | I.persulcatus | Russia: Sverdlovsk region, Beryozovskyi district | Kovalev et al. (2013) |
| Ekb1766-1977 | GU444152 | 3Unique | 1977 | I.persulcatus | Russia: Ural, Sverdlovsk region, Nizhnye Sergi district | Kovalev et al. (2009) |
| Ekb17698-2011 | JX315953 | 3Unique | 2011 | I.persulcatus | Russia: Sverdlovsk region | Kovalev et al. (2012) |
| Ekb1852-2011 | JX315894 | 3Unique | 2011 | I.persulcatus | Russia: Nizhnyi Tagil, Sverdlovsk region | Kovalev et al. (2012) |
| Ekb1867-2006 | JX315745 | 3Unique | 2006 | I.persulcatus | Russia: Yekaterinburg, Sverdlovsk region | Kovalev et al. (2012) |
| Ekb190507-2005 | JX315726 | 3Unique | 2005 | I.persulcatus | Russia: Yekaterinburg, Sverdlovsk region | Kovalev et al. (2012) |
| Ekb197-I-2007 | JX315772 | 3Unique | 2007 | Human blood | Russia: Kamyshlov, Sverdlovsk region | Kovalev et al. (2012) |
| Ekb200505-2-2005 | GU444187 | 3Unique | 2005 | I.persulcatus | Russia: Ural, Sverdlovsk region, Sysert' district | Kovalev et al. (2009) |
| Ekb200605-2005 | JX315729 | 3Unique | 2005 | I.persulcatus | Russia: Kosmakovo, Sysert' district, Sverdlovsk region | Kovalev et al. (2012) |
| Ekb2008-2006 | GU444216 | 3Unique | 2006 | I.persulcatus | Russia: Ural, Sverdlovsk region, Polevskoi district | Kovalev et al. (2009) |
| Ekb206-2010 | JX315831 | 3Unique | 2010 | I.persulcatus | Russia: Irbit district, Sverdlovsk region | Kovalev et al. (2012) |
| Ekb207-2010 | JX315832 | 3Unique | 2010 | I.persulcatus | Russia: Irbit district, Sverdlovsk region | Kovalev et al. (2012) |
| Ekb2075-2006 | JX315746 | 3Unique | 2006 | I.persulcatus | Russia: Revda, Sverdlovsk region | Kovalev et al. (2012) |
| Ekb208-2009 | JX315991 | 3Unique | 2009 | I.persulcatus | Russia: Yekaterinburg, Sverdlovsk region | Kovalev et al. (2012) |
| Ekb2490-1969 | GU444132 | 3Unique | 1969 | Human blood | Russia: Ural, Sverdlovsk region, Nizhnyi Tagil district | Kovalev et al. (2009) |
| Ekb24974-2009 | JX315996 | 3Unique | 2009 | Human blood | Russia: Sverdlovsk region + | Kovalev et al. (2012) |
| Ekb256-2007 | GU444227 | 3Unique | 2007 | I.persulcatus | Russia: Ural, Sverdlovsk region, Nizhnyi Tagil district | Kovalev et al. (2009) |
| Ekb27-2-2007 | JX315767 | 3Unique | 2007 | I.persulcatus | Russia: Polevskoi district, Sverdlovsk region | Kovalev et al. (2012) |
| Ekb2761-2005 | GU444193 | 3Unique | 2005 | I.persulcatus | Russia: Ural, Sverdlovsk region, Verkhnyaya Pyshma | Kovalev et al. (2009) |
| Ekb279-2012 | KC886332 | 3Unique | 2012 | I.persulcatus | Russia: Sverdlovsk region | Kovalev et al. (2013) |
| Ekb3102-1986 | GU444127 | 3Unique | 1986 | I.persulcatus | Russia: Ural, Sverdlovsk region, Beloyarsk | Kovalev et al. (2009) |
| Ekb320-2009 | JX315981 | 3Unique | 2009 | I.persulcatus | Russia: Kamyshlov, Sverdlovsk region | Kovalev et al. (2012) |
| Ekb324-1975 | GU444149 | 3Unique | 1975 | I.persulcatus | Russia: Ural, Sverdlovsk region, Baikalovo district | Kovalev et al. (2009) |
| Ekb324-2-1975 | GU444156 | 3Unique | 1975 | I.persulcatus | Russia: Ural, Sverdlovsk region, Baikalovo district | Kovalev et al. (2009) |
| Ekb327-2010 | JX315851 | 3Unique | 2010 | I.persulcatus | Russia: Bogdanovich, Sverdlovsk region | Kovalev et al. (2012) |
| Ekb35-2007 | GU444233 | 3Unique | 2007 | I.persulcatus | Russia: Ural, Sverdlovsk region, Polevskoi district | Kovalev et al. (2009) |
| Ekb370-2011 | JX315920 | 3Unique | 2011 | I.persulcatus | Russia: Talitsa district, Sverdlovsk region | Kovalev et al. (2012) |
| Ekb39-2007 | GU444234 | 3Unique | 2007 | I.persulcatus | Russia: Ural, Sverdlovsk region, Polevskoi district | Kovalev et al. (2009) |
| Ekb431-2011 | JX315922 | 3Unique | 2011 | I.persulcatus | Russia: Asbest district, Sverdlovsk region | Kovalev et al. (2012) |
| Ekb4338-1978 | GU444136 | 3Unique | 1978 | I.persulcatus | Russia: Ural, Sverdlovsk region, Nizhnye Sergi district | Kovalev et al. (2009) |
| Ekb4467-2005 | GU444177 | 3Unique | 2005 | I.persulcatus | Russia: Ural, Sverdlovsk region, Bogdanovich district | Kovalev et al. (2009) |
| Ekb447-2011 | JX315952 | 3Unique | 2011 | I.persulcatus | Russia: Beryozovsk district, Sverdlovsk region | Kovalev et al. (2012) |
| Ekb467-2005 | GU444191 | 3Unique | 2005 | I.persulcatus | Russia: Ural, Sverdlovsk region, Novouralsk district | Kovalev et al. (2009) |
| Ekb606-2012 | KC886342 | 3Unique | 2012 | I.persulcatus | Russia: Sverdlovsk region, Nizhnyi Tagil district | Kovalev et al. (2013) |
| Ekb612-2010 | JX315877 | 3Unique | 2010 | I.persulcatus | Russia: Hizhnyi Tagil, Sverdlovsk region | Kovalev et al. (2012) |
| Ekb620-2010 | JX315879 | 3Unique | 2010 | I.persulcatus | Russia: Niznyi Tagyl, Sverdlovsk region | Kovalev et al. (2012) |
| Ekb622-2010 | JX315881 | 3Unique | 2010 | I.persulcatus | Russia: Berkhnyaya Salda, Sverdlovsk region | Kovalev et al. (2012) |
| Ekb637-2006 | JX315755 | 3Unique | 2006 | I.persulcatus | Russia: Yekaterinburg, Sverdlovsk region | Kovalev et al. (2012) |
| Ekb643-1978 | GU444160 | 3Unique | 1978 | I.persulcatus | Russia: Ural, Sverdlovsk region, Baikalovo district | Kovalev et al. (2009) |
| Ekb650-2006 | JX315757 | 3Unique | 2006 | I.persulcatus | Russia: Yekaterinburg, Sverdlovsk region | Kovalev et al. (2012) |
| Ekb663-2012 | KC886347 | 3Unique | 2012 | I.persulcatus | Russia: Sverdlovsk region, Nizhnyi Tagil district | Kovalev et al. (2013) |
| Ekb733-2006 | JX315760 | 3Unique | 2006 | I.persulcatus | Russia: Yekaterinburg, Sverdlovsk region | Kovalev et al. (2012) |
| Ekb746-2005 | JX315740 | 3Unique | 2005 | I.persulcatus | Russia: Bogdanovich, Sverdlovsk region | Kovalev et al. (2012) |
| Ekb77-2005 | GU444161 | 3Unique | 2005 | I.persulcatus | Russia: Ural, Sverdlovsk region, Yekaterinburg district | Kovalev et al. (2009) |
| Ekb790-2007 | JX315769 | 3Unique | 2007 | I.persulcatus | Russia: Yekaterinburg, Sverdlovsk region | Kovalev et al. (2012) |
| Ekb825-2010 | JX315882 | 3Unique | 2010 | Human brain | Russia: Artyomovskyi, Sverdlovsk region | Kovalev et al. (2012) |
| Ekb837-2006 | GU444207 | 3Unique | 2006 | I.persulcatus | Russia: Ural, Sverdlovsk region, Yekaterinburg district | Kovalev et al. (2009) |
| Ekb875-2010 | JX315885 | 3Unique | 2010 | Human brain | Russia: Severouralsk, Sverdlovsk region | Kovalev et al. (2012) |
| Ekb9-2007 | GU444247 | 3Unique | 2007 | I.persulcatus | Russia: Ural, Sverdlovsk region, Nyzhnyi Tagil district | Kovalev et al. (2009) |
| Ekb952-2005 | JX315728 | 3Unique | 2005 | I.persulcatus | Russia: Yekaterinburg, Sverdlovsk region | Kovalev et al. (2012) |
| Ekb-Gorlin-2007 | GU444217 | 3Unique | 2007 | Human brain | Russia: Ural, Sverdlovsk region | Kovalev et al. (2009) |
| Est3535 | DQ393774 | 3Unique | 2001 | I. persulcatus | Estonia | Golovljova et al. (2004) |
| IR99-2f13 | AB049353 | 3Unique | 1999 | I .persulcatus | Irkutsk, Russia | Hayasaka et al. (2001) |
| IR99-2m3 | AB049350 | 3Unique | 1999 | I. persulcatus | Irkutsk, Russia | Hayasaka et al. (2001) |
| Kemerovo-8-11-05 | FJ214135 | 3Unique | 2005 | I. persulcatus | Kemerovo, West Siberia, Russia | Karan et al. (2008) |
| Kokkola-25 | DQ451288 | 3Unique | 2006 | I. persulcatus | Finland | Jaaskelainen et al. (2005) |
| Kokkola-26 | DQ451289 | 3Unique | 2006 | I. persulcatus | Finland | Jaaskelainen et al. (2005) |
| Kokkola-84 | DQ451293 | 3Unique | 2006 | I. persulcatus | Finland | Jaaskelainen et al. (2005) |
| Kolarovo-2008 | FJ968751 | 3Unique | 2008 | Ixodes pavlovskyi | Kolarovo, Tomsk region, Russia | Chausov et al. (2009) |
| M2 | JQ693480 | 3Unique | 2011 | ixodid tick | Moscow region, Russia | Morozov et al. (2012) |
| MucAr_M14/10_Mongolia | JF274481 | 3Unique | 2010 | I. persulcatus | Mongolia, Bulgan Aimak | Frey et al. (2011) |
| MucArM14-10 | JQ429588 | 3Unique | 2010 | I. persulcatus | Mongolia | Frey et al. (2012) |
| Omsk4005-2010 | JX315859 | 3Unique | 2010 | I.persulcatus | Russia, Omsk region | Kovalev et al. (2012) |
| Omsk4018-2010 | JX315863 | 3Unique | 2010 | I.persulcatus | Russia, Omsk region | Kovalev et al. (2012) |
| Omsk4044-2010 | JX315867 | 3Unique | 2010 | I.persulcatus | Russia, Omsk region | Kovalev et al. (2012) |
| Omsk4050-2008 | JX315784 | 3Unique | 2008 | I.persulcatus | Russia, Omsk region | Kovalev et al. (2012) |
| Omsk4069-2-2010 | JX315871 | 3Unique | 2010 | I.persulcatus | Russia, Omsk region | Kovalev et al. (2012) |
| Omsk4074-2010 | JX315872 | 3Unique | 2010 | I.persulcatus | Russia, Omsk region | Kovalev et al. (2012) |
| Prm71826-2011 | JX315933 | 3Unique | 2011 | I.persulcatus | Russia, Perm' region | Kovalev et al. (2012) |
| Prm72318-2011 | JX315908 | 3Unique | 2011 | I.persulcatus | Russia, Perm' region | Kovalev et al. (2012) |
| Prm73547-2011 | JX315934 | 3Unique | 2011 | I.persulcatus | Russia, Perm' region | Kovalev et al. (2012) |
| Tmn3017-2010 | JX315834 | 3Unique | 2010 | I.persulcatus | Russia, Tyumen region | Kovalev et al. (2012) |
| Tmn3037-2010 | JX315843 | 3Unique | 2010 | I.persulcatus | Russia, Tyumen region | Kovalev et al. (2012) |
| Tmn3041-2010 | JX315847 | 3Unique | 2010 | I.persulcatus | Russia, Tyumen region | Kovalev et al. (2012) |
| Tmn4024-2008 | JX315796 | 3Unique | 2008 | I.persulcatus | Russia, Tyumen region | Kovalev et al. (2012) |
| Tmn5014-2007 | JX315774 | 3Unique | 2007 | I.persulcatus | Russia, Tyumen region | Kovalev et al. (2012) |
| Tmn5015-2007 | JX315775 | 3Unique | 2007 | I.persulcatus | Russia, Tyumen region | Kovalev et al. (2012) |
| Tmn5016-2007 | JX315779 | 3Unique | 2007 | I.persulcatus | Russia, Tyumen region | Kovalev et al. (2012) |
| Tmn5018-2007 | JX315778 | 3Unique | 2007 | I.persulcatus | Russia, Tyumen region | Kovalev et al. (2012) |
| Vasilchenko | AF069066 | 3Unique | 1969 | Human blood | Novosibirsk, Russia | Gritsun et al. (1998) |
| Vasilchenko | AF069066 | 3Unique | 1969 | Human blood | Novosibirsk, Russia | Gritsun et al. (1998) |
| Volkhov-2-43 | FJ214148 | 3Unique | 1943 | I. ricinus | Volkhov, northwestern region, Russia | Karan et al. (2008) |
| Vologda-157-08 | GQ845434 | 3Unique | 2008 | I. persulcatus | Russia, European part, North-West, Vologda | Karan et al. (2009) |
| Vologda-227-07 | FJ214153 | 3Unique | 2007 | I. persulcatus | Vologda, northwestern region, Russia | Karan et al. (2008) |
| Vologda-365-75 | FJ214136 | 3Unique | 1975 | I. persulcatus | Vologda, northwestern region, Russia | Karan et al. (2008) |
| Ya 10/89 | GU125719 | 3Unique | 1989 | ? | ? | Kozlovskaya et al. (2009) |
| Yar 114 | EU444078 | 3Unique | 2001 | I. persulcatus | Yaroslavl region, Russia | Khasnatinov (2008) |
| Yar 46-2 | EU444079 | 3Unique | 2001 | TBE patient | Yaroslavl region, Russia | Khasnatinov (2008) |
| Yar 48 | EU444080 | 3Unique | 2000 | I. persulcatus | Yaroslavl region, Russia | Khasnatinov (2008) |
| Yar 71 | EU444077 | 3Unique | 1999 | I. persulcatus | Yaroslavl region, Russia | Khasnatinov (2008) |
| Yaroslavl-2-80 | FJ214144 | 3Unique | 1980 | I. persulcatus | Yaroslavl, Central region, Russia | Karan et al. (2008) |
| 2530 | GU060547 | 3Unique | 2009 | I.persulcatus | Russia, South-Western Siberia, Novosibirsk region | Morozova et al. (2009) |
| 2730 | JN993573 | 3Unique | 2011 | I. pavlovskyi | Russia: South-Western Siberia, Novosibirsk | Morozov et al. (2011) |
| Vologda-658-75 | FJ214137 | 3Unique | 1975 | Human blood | Vologda, northwestern region, Russia | Karan et al. (2008) |

* - The sequence is available upon request
